# A mechanistic explanation of the transition to simple multicellularity in fungi

**DOI:** 10.1038/s41467-020-16072-4

**Published:** 2020-05-22

**Authors:** Luke L. M. Heaton, Nick S. Jones, Mark D. Fricker

**Affiliations:** 1 Department of Plant Sciences, University of Oxford, South Parks Road, Oxford, OX1 3RB UK; 2 Department of Mathematics, Imperial College London, Imperial College Road, London, SW7 2AZ UK

**Keywords:** Computational models, Evolutionary theory, Fungi

## Abstract

Development of multicellularity was one of the major transitions in evolution and occurred independently multiple times in algae, plants, animals, and fungi. However recent comparative genome analyses suggest that fungi followed a different route to other eukaryotic lineages. To understand the driving forces behind the transition from unicellular fungi to hyphal forms of growth, we develop a comparative model of osmotrophic resource acquisition. This predicts that whenever the local resource is immobile, hard-to-digest, and nutrient poor, hyphal osmotrophs outcompete motile or autolytic unicellular osmotrophs. This hyphal advantage arises because transporting nutrients via a contiguous cytoplasm enables continued exploitation of remaining resources after local depletion of essential nutrients, and more efficient use of costly exoenzymes. The model provides a mechanistic explanation for the origins of multicellular hyphal organisms, and explains why fungi, rather than unicellular bacteria, evolved to dominate decay of recalcitrant, nutrient poor substrates such as leaf litter or wood.

## Abstract (web summary)

Multicellularity is one of the major transitions in evolution. Here, authors use a model to show that compared to unicellular bacteria, multicellular fungi can more rapidly colonise immobile, nutrient poor resources because exoenzymes provide greater or longer lasting benefits to mycelial organisms.

## Introduction

There is a well-established trend over evolutionary time for organisms to increase in size and complexity^1,2^, with multicellular lineages emerging independently from unicellular ancestors multiple times in algae, plants, animals, and fungi^3–8^. Plausible mechanistic explanations for the transition to multicellularity have focussed on the potential benefits arising from the division of labour, particularly germ-line and soma^9^, predator escape^10^, enhanced stress resistance^11^, or circumventing diffusion limits for nutrient acquisition or distribution^12–14^. In most eukaryotic lineages, the transition to multicellularity is thought to involve co-option and expansion of gene families for adhesion, signalling, and cell–cell transport^12,15^, whilst division of labour and differentiation are typically associated with innovation and expansion of new transcription factor families^12,15^. However, recent comparative genome analyses of fungal lineages do not show a marked increase in gene duplications or the typical proliferation of kinases, receptors, or adhesion protein families expected within this general framework^8,16,17^. Rather, these studies highlight the importance of co-option and modification of existing gene families, particularly those governing endomembrane complexity, cytoskeletal transport, cell-wall biogenesis, septal pore gating, and osmotrophy^8,17,18^.

The evolution of multicellular fungi was a critical event in the emergence of terrestrial ecosystems^19–22^. Extant fungi now account for the second largest fraction of biomass in terrestrial ecosystems after plants^23^, and they are critically involved in soil formation, wood decomposition, and nutrient cycling^24–26^. Genetic evidence indicates that all fungi are descended from a motile aquatic ancestor^27^, with subsequent loss of flagella and the development of multicellular hyphal growth linked to adaptation to land environments^8,20–22,28^. Whilst the genomic basis underpinning the transition from a unicellular to a multicellular hyphal state, and subsequent development of complex multi-cellular fruiting bodies is beginning to emerge^8,16,17,29^, the drivers behind these changes and mechanistic explanations of their success are currently lacking.

Many fungi are osmotrophic^30^ and acquire carbon (C), nitrogen (N), and phosphate (P) from the environment by uptake of low-molecular-weight compounds directly, through solubilisation of mineral phosphate^31^, or following extracellular digestion using exoenzymes to breakdown high-molecular-weight carbohydrates, organic phosphates, protein, or other recalcitrant polymers such as pectins, chitin, and lignin^20,24,26^. Indeed, genetic evidence suggests that the production of exoenzymes to digest plant or algal cell walls is an ancestral trait, found in unicellular chytrids^28^ that predate the earliest forms of hyphal multicellularity, or adaptation to terrestrial environments^20,21^. Terrestrial mycelial fungi grow by apical extension and branching of hyphae, with coenocytic cell compartments in primitive species, or partitioned by septa in basidiomycetes and ascomycetes, where cytoplasmic continuity is maintained through septal pores^32–34^. The polarised growth and fractal-like branching of mycelial fungi constitute an efficient space-searching strategy^35^, and large interconnected mycelial networks can span soil gaps and patches of nutrients^24,32,36^. These distinctive features of fungal growth are evolutionarily adaptive, but they do not explain why fungi and, to a lesser extent, hyphal actinomycetes, rather than unicellular bacteria, evolved to dominate the decay of hard to digest, nutrient-poor substrates.

The amount of C, N, and P available depends on the resource environment^37^, and many environments have a C:N:P ratio that is very different from the optimal ratio for growth. Typical internal C:N and N:P molar ratios for bacteria and fungi themselves also span a wide range from 5-200:1 and 4-20:1, respectively^38–40^. Growth also requires additional acquisition of C to fuel respiration, and carbon use efficiencies (CUE) of 0.5 or less are typical^41,42^. Cells can survive if they are only supplied with a source of energy (C), but they cannot grow and divide unless they also have a supply of N, P, and other micro-nutrients. It follows that nutrient supply has a critical impact on growth rates. Colonisation of land presents additional challenges as net movement of soluble nutrients is reduced in terrestrial compared to aquatic environments, as the soil pore tortuosity significantly increases the path length for diffusion. Furthermore, the requirement to breakdown C-rich polymers produces even greater spatial inhomogeneities in nutrient concentration as these do not diffuse at all.

Here, we develop a modelling framework to compare the predicted growth of hyphal organisms in comparison with immobile, autolytic, or motile unicellular osmotrophs in a wide range of resource environments which differ in the C:N:P ratio and the recalcitrance of the substrate. We show that hyphal osmotrophs outperform unicellular osmotrophs whenever the local resource is immobile, hard-to-digest, and nutrient-poor. This hyphal advantage arises because transporting nutrients via a contiguous cytoplasm enables continued exploitation of remaining resources after local depletion of essential nutrients, so in hyphal organisms, each exoenzyme is of benefit for longer, providing greater total benefit for the same cost. The model provides a mechanistic explanation for the origins of multicellular hyphal organisms, and explains why fungi, rather than unicellular bacteria, evolved to dominate decay of recalcitrant, nutrient-poor substrates such as leaf litter or wood. The model also suggests that multicellular fungi were pre-adapted to form mycorrhizal associations.

## Results

### Nutrient availability limits colony growth

**Fig. 1:**
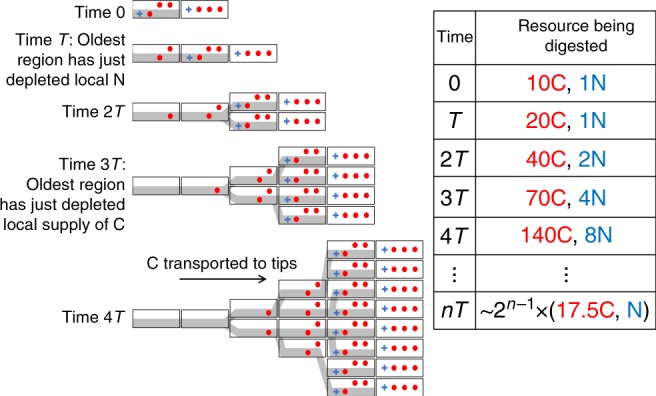
Exponential hyphal growth enables an efficient biological Ponzi scheme for resource acquisition. Each block represents a unit volume, containing external resource (white compartment) comprised of 30 units of C (three red circles) and one unit of N (one blue cross). Hyphae (grey region) consume these resources as they grow, and can also translocate resources throughout the colony to fuel new growth. If the time taken to exhaust the local supply of N and C is $T$ and $mT$ respectively (illustrated here for $m = 3$), and over time $T$ the colony can grow by a factor of 2, the fraction of the colony that has exhausted the local supply of N and C reaches a constant $2^{ - 1}$ and $2^{ - m}$, respectively, after an initial period of length $T$ and $mT$, respectively. If unicellular organisms and hyphal organisms take up N in the same way and need the same amount of carbon for each unit volume of growth, each growing cell in a unicellular colony would need to take up 17.5 C for each N instead of 10 C for each N, and would therefore need to synthesise a correspondingly larger number of C digesting exoenzymes.

Due to the relatively low level of mixing in terrestrial habitats, we note that there are two distinct ways in which the growth of a terrestrial osmotroph may be nutrient-limited. The supply rate can be limiting (too little nutrient obtained for each unit time), or the total local supply may be limiting (only a small amount of nutrient is locally available, however rapidly it is extracted). The contiguous cytoplasm of fungi^32,34^ or hyphal actinomycetes^43,44^ enables internal transport, and here we argue that internal transport is evolutionarily adaptive if the growth of the colony is limited by the exhaustion of local nutrient supplies. In particular, we note that when a hyphal organism encounters enough resource to grow exponentially, it can sustain a digestive strategy whereby all nutrients in the substrate are digested, yet over each window of time, the resource acquired by the whole colony has a much better C:N ratio than the substrate (see Fig. 1). This beneficial mismatch in C:N ratios is akin to a biological Ponzi scheme, which can only be sustained as long as the colony has an exponentially expanding feeding surface, with newly grown regions prioritising the acquisition of growth-limiting nutrients.

The advantage conferred by a hyphal morphology can be clarified by a simple toy model comparing an established colony of unicellular organisms and an established fungal colony (Fig. 1). Each colony grows through an initially homogeneous environment, consuming the local supply of C and N at a constant rate until the local supply is exhausted. If the substrate has a molar C:N ratio 100:1, but cells only require 10 C atoms for every N atom to match internal demand and respiration, 90% of the carbon in the substrate will be undigested by the time the local supply of N has been exhausted. Once the local supply of N is exhausted unicellular organisms can no longer grow, even though they contain the synthetic machinery needed for growth, and are surrounded by exoenzymes that continue to release a supply of reduced C. In contrast, the N depleted region in a fungal colony can continue to access C, digesting it completely and supplying C to the growing margin by transport through the cytoplasmic continuity of the interconnected network.

If the local supply of N becomes exhausted after time *T*, then at time $t$, the volume of territory with an exhausted supply of N is the volume grown at time $t - T$. If the growth is exponential with a specific growth rate $\mu$, it follows that the fraction of territory with an exhausted supply of N is $\frac{{e^{\mu (t - T)}}}{{e^{\mu t}}} = e^{ - \mu T}$. In the extreme case where the time taken to exhaust the local supply of N is equal to the doubling time of the colony ($T = \frac{{\mathrm{log}(2)}}{\mu }$), half the colony will have exhausted the local supply of N. If, where both kinds of resource are available, a fungus takes up 10 C atoms for every N atom, the time taken to exhaust the local supply of C will be $10T$, only a fraction $e^{ - 10\mathrm{log}(2)} = 0.1\%$ of the colony will have exhausted the local supply of C, and the fungal colony will receive almost half its C from the N depleted region. If the fungus digests N at the same rate as unicells, it only has to digest C at half the rate to receive the same overall amount of C and N as the growing part of a colony of unicells, so the fungus requires a much smaller investment in C digesting exoenzymes. Whether the fungal colony receives more carbon for each unit volume, or invests less heavily in C digesting exoenzymes, it obtains an advantage over unicellular competitors, provided that the additional costs of internal transport are covered.

### Nutrient supply and demand depend on strategy for growth

**Fig. 2:**
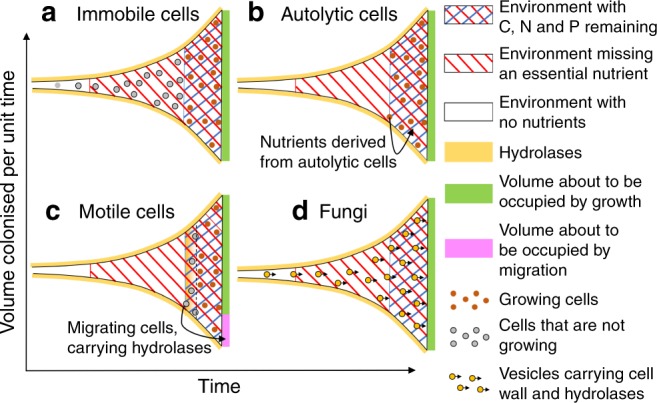
Local exhaustion of essential nutrients constrains the growth rate of colonies of osmotrophs. **a** The reduction in growth rate following local nutrient exhaustion is greatest for immobile cells, that cannot contribute to colony growth once any essential nutrient is locally exhausted. **b** The effect is partially mitigated for autolytic cells, which recycle nutrients from dead cells. **c** Motile cells swim to new locations once nutrients are exhausted, leaving exoenzymes behind. **d** Fungal colonies remain connected and can therefore translocate resources internally. All colonies are shown growing exponentially, and their growth strategies result in different zones of cell behaviour and nutrient depletion, as shown in the figure legend.

To quantify the scale of the advantage conferred by a hyphal morphology in various resource environments, we compare the growth of four categories of osmotrophic organism: immobile cells that occupy new territory solely by growth and division (Fig. 2a); autolytic cells that recycle material from redundant cells once nutrients are exhausted, with the most likely beneficiaries being neighbouring kin^45,46^ (Fig. 2b); motile cells that are able to migrate to find a new resource (Fig. 2c); and hyphal organisms that grow into new territory but remain connected through the hyphal network (Fig. 2d).

Our focus is the competition to capture resource, so we model the early stages of colonisation, before crowding effects limit growth. As a simplifying assumption, we suppose that all organisms, and all generations, capture the same quantity of resource for each unit volume of growth. Thus we assume that each category of model organism grows exponentially on encountering a new resource (Fig. 2). Growth under crowded conditions where organisms overlap makes the model more complex but does not change the overall conclusions (Supplementary Note 8).

The rate of substrate digestion depends on the quantity of exoenzymes that have been secreted, and we assume that for each key resource (C, N, P) the local rate of digestion is proportional to the mass of exoenzymes released for each unit volume of organism. Rates of digestion are also inversely proportional to the recalcitrance of the substrate, $\tau$, which we define as the time taken for each type of exoenzyme to supply the organism with a mass of nutrients (C, N, or P) equal to the total mass required to synthesise the exoenzyme in question, including the C required for respiration (see Supplementary Table 1 for a description of all model parameters). The time scale $\tau$ depends on abiotic factors such as temperature and pH, as well as the efficiency of the exoenzymes, the carbon cost of protein synthesis, and the physical accessibility and chemical composition of the substrate. For the sake of simplicity, we assume that all exoenzymes generate a constant supply of C, N, or P until the local resource is exhausted, at which point the local supply rate is set to zero. We believe this is a reasonable approximation for processive enzymes that break down polymers. However, as N-rich polymers tend to be embedded in a C-rich matrix, a certain amount of C digestion may be required to access the other resources. We consider the consequences of such physical shielding, set by the parameter $\delta$, in Supplementary Note 9.

Immobile cells grow until they have exhausted the local supply of either C, N, or P, at which point they are no longer able to make any contribution to the growth of the colony (Fig. 2a). Data suggests that when nutritionally stressed cells recycle their nutrient contents through autolysis, the growth rate of neighbouring cells is increased^47^. In our model, we assume that when autolytic cells (Fig. 2b) exhaust the local resource, they release a fraction $\varepsilon$ of the cell’s contents, making that resource freely available to the remaining cells^45,46^, although any resource used to synthesise the exoenzymes cannot be recouped. The fraction $\varepsilon$ is set to 50% here, but this parameter is tunable, and even 100% recovery does not affect the model conclusions (see Supplementary Fig. 1). Motile cells are modelled as switching between two states (Fig. 2c). In state 1 they grow like other cells, but they stop growing when they exhaust the local supply of P, or when there is just enough C and N remaining to synthesise the exoenzymes they will need when they migrate to a new location. In state 2 motile cells synthesise and store those additional exoenzymes, then migrate to a new location, releasing the stored exoenzymes and returning to state 1. For fungi (Fig. 2d), exoenzymes that are initially secreted at the hyphal tip continue to release nutrients as the colony grows. These nutrients can be taken up and transported through the mycelium to the growing margin until they are locally exhausted, allowing complete exploitation of the available resource, even if it comprises a mismatched C:N:P ratio.

**Fig. 3:**
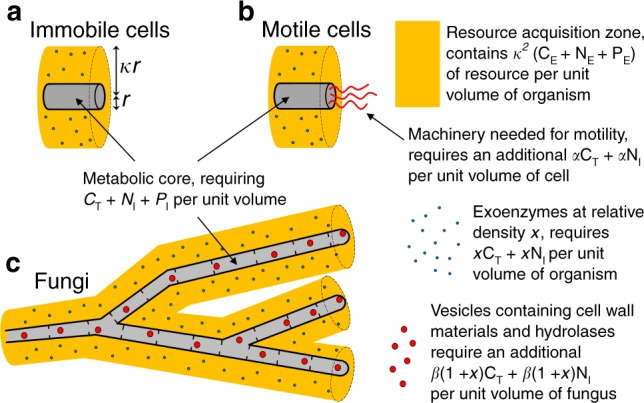
Nutrient supply and demand depends on the type of organism, and the density of exoenzymes. C_E_, N_E_, and P_E_ denote the density of the relevant nutrients in the environment (yellow), and we assume that cylindrical cells and hyphae obtain resource over a distance $\kappa$ times greater than the cell radius (**a**). The metabolic core of all organisms requires C_T_, N_I_, and P_I_ for each unit volume of growth (grey). The total mass of C and N required for exoenzymes (blue dots) is equal to *x*C_T_ + *x*N_I_ for each unit volume of organism, where in each case the relative density $x$ is chosen to maximise growth rates. Motile cells (**b**) and fungi (**c**) require additional resource for each unit volume (red shapes), to enable motility and internal transport, respectively. These additional costs are represented by the parameters $\alpha = 0.02$^48^ and $\beta = 0.1$, respectively.

The total amount of C, N, and P in the environment are set by the model parameters C_E_, N_E_, and P_E_ (see Fig. 3) which are varied across a wide range of values spanning the different resource environments found in nature^24,38^. The density of C, N, and P within immobile cells (C_I_, N_I_, P_I_) are also specified as model parameters (Supplementary Note 1). Since organisms lose C through respiration, growing a unit volume of immobile cell requires a total mass of carbon *C*_T_ > *C*_I_, and this total carbon cost of growing a unit volume is specified by including the CUE as a model parameter.

Synthesising motile apparatus requires additional C and N, set to be 2% of the cost of synthesising a cell^48^, and represented by the parameter $\alpha$ (see Fig. 3b). Fungi also incur additional costs as exoenzymes and other materials needed at the tips need to be transported, and, while those materials are in transit, they are not yet helping liberate new food resource. The additional costs associated with internal transport are unknown, but are represented by a tunable parameter $\beta = 0.1$, i.e., 10% of the total C and N budget of the cell (see Fig. 3c). The fraction $\beta$ can be visualised as the fraction of C and N that is contained in exoenzymes or cell wall material moving within transport vesicles, although in modelling terms, the transported material could equally well be precursors moving cytoplasmically. The critical point is that they are transported over distance. Note that our choice of $\beta$ is such that the cost of internal transport is at least 5 times higher than cost of motility, which ‘penalises’ early evolutionary development of a hyphal morphology in comparison with other strategies. We present results for a range of values for $\beta$ in Supplementary Figs. 2 and 3.

We assume all categories of organism can obtain low molecular weight molecules following digestion by diffusion over an external length scale $\kappa$ times greater than the cell radius (see Fig. 3a). The mass of C, N, and P digesting exoenzymes for each unit volume of organism are treated as variables, and in any given case we can calculate the demand for C, N, and P for each unit volume of growth, and the supply rate of C, N, and P for each unit volume of the colony. Thus for each category of organism, and each density of exoenzymes, we can calculate the maximum growth rate, given the mass required for each unit volume of growth, and given that C, N, and P cannot be used more rapidly that they are digested. The maximum growth rate is also constrained by the rates of transcription and translation, so we assume that for any category of organism, the total rate of resource use for each unit volume cannot exceed $\lambda$ = 0.3 g ml^−1^ h^−1^, as this ensures that the smallest doubling time for any organism in any resource environment is 51 min, which is below the typical shortest doubling time for either prokaryotic or eukaryotic cells expected in the wild^49–51^.

In our model, the volume colonised for each unit time will increase in proportion to the size of the colony, and we define the apparent growth rate of a colony as the volume of resource that is colonised for each unit time, for each unit volume that has already been colonised. As our model specifies all the relevant quantities, for any given density of exoenzymes, we can calculate the apparent growth rate of the colony, and thereby find the optimal density of exoenzymes and the maximal rate of colonisation for any given category of organism. The full set of equations describing maximal colony growth for each category of organism are given in Supplementary Notes 2–5.

### Maximal colonisation rates differ between types of organism

**Fig. 4:**
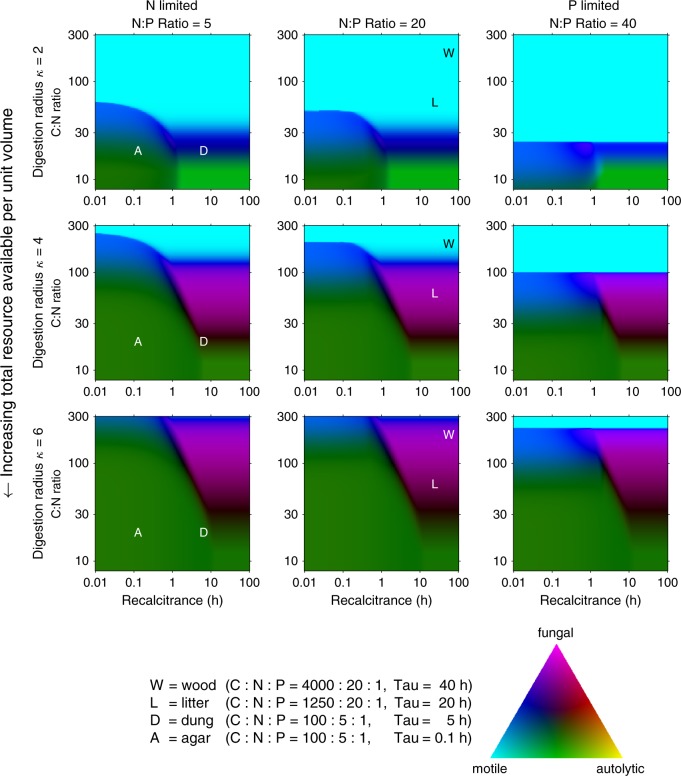
Relative growth rates of osmotrophs depend on resource availability and substrate recalcitrance. Each panel indicates relative performance of each category of organism as the C:N:P ratio and recalcitrance, $\tau$, are varied over a wide but physiologically relevant range, shown on log scales. Results are shown after the fastest-growing colony has increased in size by a factor of 1000, and the colour in each pixel is proportional to the increase in size of a colony of fungi (magenta), motile cells (cyan), and autolytic cells (yellow), according to the inset triangle. Rows correspond to the increasing amount of resource availability as $\kappa$ is varied from 2, 4 to 6 cell radii (see Fig. 2). Columns correspond to varying N:P ratios set to 5:1, 20:1, and 40:1. The substrate has a dry mass of 0.5 g ml^−1^ in all cases.

**Fig. 5:**
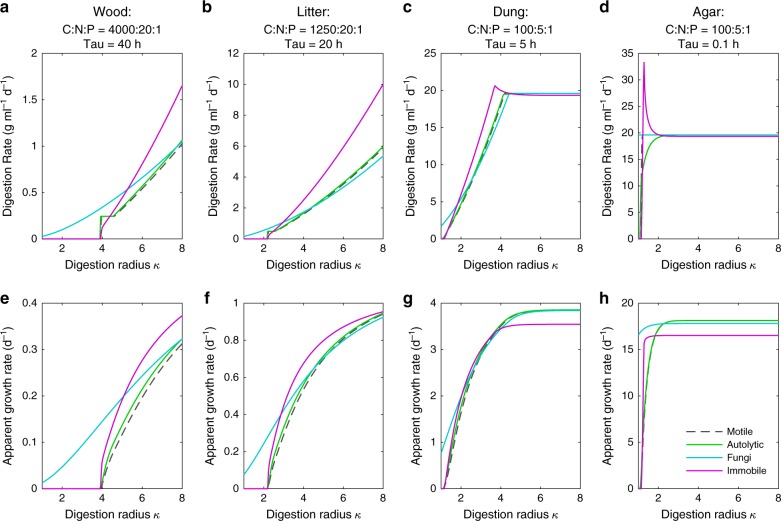
Resource availability determines the optimal rate of digestion and relative rate of colonisation. Panels (**a**–**d**) show the optimal rates of digestion for fungi, motile, autolytic, and immobile cells, and panels (**e**–**h**) show the corresponding rates of colonisation for each unit volume colonised, for varying total amounts of resource availability set by the radius of digestion $\kappa$, on wood (**a**, **e**), leaf litter (**b**, **f**), fresh dung (**c**, **g**), or malt agar (**d**, **h**). Each type of substrate is modelled by assuming an appropriate C:N:P ratio and recalcitrance value $\tau$. In each case, the total dry mass for each unit volume of the substrate is 0.5 g ml^−1^, and growing the metabolic core of an organism requires a total of 0.33 g ml^−1^, at a C:N:P ratio of 168:14:1. These figures account for both the material contained in the organism and the C lost in respiration set by the CUE (see Supplementary Note 6). Note that when the environment contains enough resource for fungi to grow, fungi almost always have the highest rate of digestion (**a**–**d**). Also note that across the different media, growth rates vary by nearly 2 orders of magnitude according to the *y*-axis units (**e**–**h**). C:N:P ratios were taken from the literature^39,41^, and recalcitrance values were set to match growth rates that are typically observed on each type of substrate.

We compare the relative performance of each class of organism for a wide range of environmental C:N:P ratios and levels of substrate recalcitrance, $\tau$. In general, the absolute growth rate of all classes of organism declines as the recalcitrance of the substrate increases, or N or P becomes limiting (Supplementary Fig. 1). However, the relative growth rate, expressed as a ratio, emphasises which organism will perform best in each substrate regime (Fig. 4). The colour coding is presented on a cyan–yellow–magenta scale, and the hue indicates the environments where motile, autolytic, and fungal organisms dominate, respectively, according to the inset colour triangle. We find that for a specific, but broad, set of substrates, fungi can colonise resource significantly faster than motile or autolytic cells (magenta to purple regions in Fig. 4). There is a significant, physiologically relevant region of parameter space where fungi outperform motile and autolytic cells. In general, large C:N ratios, large C:P ratios and highly recalcitrant substrates strongly favour hyphal morphology (Fig. 4), and for C:N:P ratios typical of wood or leaf litter, the apparent growth rate of a fungal colony is significantly higher than the growth rate of any other kind of colony, provided there is sufficient total resource available (Fig. 5e, f). The dominance of fungi in these substrate niches is maintained even if unicells are allowed extremely favourable treatment by relaxing the cost to synthesising motile apparatus ($\alpha = 0$), or autolysis is allowed to recoup 100% of the cost of synthesising cells (i.e., $\varepsilon = 1$, Supplementary Fig. 2). Furthermore, fungi still compete effectively over a reasonable substrate range even when the cost of transport ($\beta$) is increased to 20% of the total (Supplementary Fig. 3). Indeed, in some environments, fungi can grow more than twice as fast as colonies of unicells when 50% of resources are allocated to transport (Supplementary Fig. 4). However, when a unit volume captures less resource than is needed to grow a unit volume (low values of $\kappa$, Fig. 5), capturing resource by growth alone is a losing proposition. Under those conditions, motile cells are predicted to dominate (cyan regions in Fig. 4), as they are able to move to multiple sites to access the nutrients needed for duplication, provided that the resource is sufficiently water saturated to enable cell migration^52^.

When the substrate is easy to digest, but the local supply of resource is only just large enough to enable duplication, motile cells and fungi grow equally well, as both strategies can offset their costs and efficiently exploit the substrate, whilst autolytic or immobile cells grow much more slowly (blue regions in Fig. 4). However, when the local supply of resource is large enough to enable multiple cell divisions, colony growth is only slightly limited by the exhaustion of local supplies. Under those circumstances, all organisms can grow close to their maximal rate, but immobile or autolytic cells grow marginally more rapidly as they are not burdened by the additional costs imposed on motile or hyphal organisms (green regions in Fig. 4).

Maximal rates of colonisation, and the optimal densities of exoenzymes, vary significantly over different resource environments, with different optima for each category of organism (Fig. 5). In general, there is a minimum amount of total resource required for each type of organism to grow, so the digestion radius $\kappa$ must be above some minimum (Fig. 5), where that minimum depends on the C:N:P ratio and density of the available resource. However, motile cells do not have a minimal value of $\kappa$, as they can acquire sufficient resources to duplicate by migrating to multiple sites. Above this threshold, growth rates increase with resource availability until the maximum growth rate possible for a given recalcitrance is reached (Supplementary Fig. 1). The digestion rate and rate of colonisation for each environment and type of organism also depend on the amount of exoenzymes secreted. Increasing the density of exoenzymes increases the digestion and supply rate of resource, which enables individual cells to grow more rapidly. However, when growth is limited by C or N, further increasing the density of exoenzymes will reduce the number of daughter cells that can be synthesised using the fixed amount of local resource, and this reduces the overall rate of colonisation (Supplementary Note 2). Hence the optimal density of exoenzymes, and optimal rate of digestion, is generally not the density that enables individual cells to grow as rapidly as possible. Because of their ability to transport nutrients, hyphal organisms reap a greater total benefit from exoenzymes that digest non-rate limiting resource. Hence our model predicts that in most environments, the optimal density of exoenzymes, and optimal rate of digestion, will be higher for hyphal organisms than for colonies of any unicellular organism (Fig. 5). Nevertheless, as the quality and quantity of available resource increases, the additional costs of internal transport reduce the hyphal advantage, and in rich media, such as malt agar, unicellular organisms are predicted to grow faster (Fig. 5d, h).

In the early stages of colonisation, none of the cells will have exhausted the local supply of resources. However, we can also consider the case where the resources available to daughter cells are diminished due to overlap with parental cells. In this case, there is a more complex time-dependent decline in the apparent growth rate for each class of organisms, depending on the total supply of resource available (Supplementary Fig. 5). However, as the fraction of the colony that has exhausted some, but not all, essential nutrients is the part that hyphal architecture is able to exploit more effectively, the apparent growth rate of fungi declines to a lesser extent and the hyphal advantage is increased (Supplementary Note 8).

The final challenge we consider is the consequence of reduced substrate accessibility, set by the model parameter $\delta$, whereby a certain amount of C has to be digested to access N embedded with the wall polymers (Supplementary Note 9). Imposing a constraint on the ratio between the rate of C and N digestion results in fungi generating a greater proportion of excess carbon, compared to other organisms (Supplementary Fig. 7). As we have not modelled any of the potential benefits of obtaining excess C, reduced substrate accessibility inhibits fungal growth more than other classes of organism. Nevertheless, we find that fungi continue to dominate in a similar region of parameter space under this accessibility condition (Supplementary Fig. 8).

## Discussion

Our model suggests that when there is a small, exhaustible local supply of some essential nutrient, and a larger supply of other nutrients, it is evolutionarily adaptive for cells to grow as an interconnected network with a common cytoplasmic pool. As growing hyphal organisms contain an expanding volume of cytoplasm, they can only maintain a constant nuclear density by containing multiple nuclei. Hence a transition to hyphal morphologies also entails a transition to either coenocytic, or fully multicellular, modes of life. A mismatch in resource quality versus internal demand is common in many natural environments^40,41^, but fungi are particularly efficient at exploiting recalcitrant high C:N or N:P resources^24,25,42^ (Figs. 4 and 5). In general, the exoenzymes of unicellular organisms can contribute to growth for at most time $T$ (that is, the time to exhaust the most growth-limiting resource), but by forming an interconnected network that enables the transport of assimilated materials, multicellular fungi effectively extend the useful lifespan of any exoenzyme that digests a non-growth-limiting resource.

Hyphal colonies are distinctively able to exploit the region where some but not all nutrients are depleted, and the scale of the benefit associated with a hyphal morphology depends on the relative size of this region (see Fig. 2d). This fraction of the colony will be relatively small if either nutrients in the substrate are plentiful, allowing multiple cell divisions before any part of the colony is nutrient-depleted, or if all nutrients are locally exhausted over a similar time-scale. We also note that if the substrate is easy to digest, and the cost of synthesising exoenzymes is a trivial fraction of the total cost of growth, organisms obtain little benefit from extending the effective life-span of exoenzymes. Thus we argue that a hyphal morphology is evolutionarily adaptive because it enables relatively rapid growth on recalcitrant substrates that require a significant investment in exoenzymes, in which there is a mismatch in nutrient ratios such that some essential nutrient becomes locally exhausted after a small number of cell divisions, while other nutrients remain abundant. This is reflected in the increased investment in exoenzymes (Fig. 5a, b) and relative growth (Fig. 5e, f) for fungi in recalcitrant resources, such as wood and leaf litter, but poor performance in more nutrient-rich substrates, where the additional costs of transport outweigh the benefits (Fig. 5d, h). This explains why in soil, low-quality resources favour fungi, while high-quality resources favour bacteria^42^. Changes in nutrient quality and availability over the course of decomposition may also explain commonly observed temporal shifts in fungal:bacterial ratios^41^. Our argument may also explain why some algae that need to search for substrate resources have evolved a siphonous body plan, with multiple nuclei in a common cytoplasmic pool, and a capacity for indeterminate growth in size^53^. We also note that in liquid niches, where the supply of nutrients to an individual cell does not come from a local, exhaustible, microscopic patch, several fungal lineages, such as yeasts, have streamlined their genomes^54^ and reverted to a primarily unicellular lifestyle.

The advantage of a hyphal morphology is remarkably robust to changes in model parameters, most notably reducing the costs associated with motility ($\alpha$, Supplementary Fig. 2), increasing the benefit for autolytic cells ($\varepsilon$, Supplementary Fig. 2), increasing the costs of transport ($\beta$, Supplementary Figs. 3 and 4), imposing time-varying restrictions on resource availability due to crowding (Supplementary Figs. 5 and 6), or increasing the requirement to digest C to render N accessible ($\delta$, Supplementary Figs. 7 and 8).

To obtain the hyphal advantage, organisms must be able to transport nutrients across the colony. Both fungi and actinomycetes can transport materials by diffusion and growth-induced mass-flow^55,56^, but as fungi are eukaryotic, they can also use internal vesicles, the cytoskeleton and motor proteins, which enables greater control over the internal distribution of macromolecules^33,34^. Expansion in these gene families is associated with the development of multicellularity in fungi^8^. Thus fungi are able to use C to fuel growth even when the source of C is located millimetres or even metres from the source of other essential nutrients. Our analysis strongly suggests that whenever hyphal colonies emerge, the mature parts will be a source of non-rate limiting resource (C), while the growing margin obtains growth-limiting resource (N or P). This metabolic division of labour may have pre-adapted fungi to develop the mycorrhizal associations found in 85% of extant plant species, whereby N and P acquired by the fungus are traded for C fixed by the plant^57^. This symbiosis is ancient and can be traced back to the earliest land colonisers^19,58^, and our model clarifies why filamentous fungi were ideally placed to become nutrient foragers in a symbiotic partnership, once both parties evolved the ability to exchange sugars for P and N. Such exchanges require long-distance bidirectional transport^59^, which does not yet have a firm, mechanical basis. Nevertheless, bidirectional transport has been observed at both the colony level^60,61^ and in individual hyphae of both mycorrhizal^59^ and saprotrophic fungi^62^. Bidirectional movement by diffusion can also occur at a sub-cellular level within vacuolar networks over short distances^63^.

Although we have not explicitly considered heterogeneous environments containing multiple substrates with differing levels of recalcitrance^36^, our observations also explain why fungi break down lignin. For unicellular organisms, sources of C can only fuel growth and division for as long as it takes to exhaust the local supply of other essential nutrients. If cells can obtain the C they require by consuming the more readily digestible components of wood, there is no benefit from evolving mechanisms for the breakdown of the most recalcitrant forms of carbon, as the local N will already be exhausted. Conversely, when fungi release enzymes that break down lignin, the growing parts of the colony remain connected to the resulting slow but steady supply of C, and this supply reduces the need to synthesise other C digesting exoenzymes.

Our model compares the rate of colonisation of competing osmotrophs, not fitness directly, which is challenging to define for fungi^64^. Nevertheless, all organisms require a source of C to power metabolism, and we follow Van Valen^65,66^ in treating competition to capture resource (energy) as a key driver of evolutionary dynamics. In this framework the expansive energy available for growth and reproduction is a good approximation for fitness^65–67^, and circumvents the problem of needing to define and count individuals and their reproductive output central to other fitness measures, which is both conceptually and practically challenging for indeterminate or colonial organisms^64,67^. Furthermore, the impact of competition for other resources can also be accommodated in the extent that they constrain control of trophic energy^65,66^. It is argued that natural selection locally maximises the amount of expansive energy for the unit under consideration at a given time scale^65,66^. Previously we have shown that when the time to reproduce via spores is large compared to the doubling time of a fungal colony, the total energy available for reproduction is maximised when growth rates are (almost) as large as possible^68^. It follows that when osmotrophs compete for the same resource, the competitor that can grow most rapidly initially is likely to be the most fit in terms of control of C supply.

Overall, we suggest that the emergence of hyphal organisms is best explained by changes in the available opportunities for metabolic activity^69^, instead of viewing hyphal morphology as an evolutionary transition in individuality^3,4^. The absence of a major transition in individuality is not surprising, as clonal hyphal organisms, in a similar manner to siphonous coenocytic organisms^53^, have minimal cell–cell conflict and essentially bypass the alignment of fitness stage^16^, whilst the benefits of long-distance transport of nutrients through the connected cytoplasm directly leads to export-of-fitness at the level of the mycelial network^53,70^.

## Methods

### Overview of the modelling framework

The model has two interacting components, a common resource environment and a representation of each class of organism. The resource environment is defined by five parameters that can be independently varied. The density of C, N, and P in the environment are given by C_E_, N_E_, and P_E_ in g ml^−1^. In practice, these are set by the C:N and N:P ratio of the resource, to allow easy comparison with literature values, and the total resource density in g ml^−1^. To accommodate different levels of substrate digestibility, each substrate also has a level of recalcitrance, $\tau$ (h), which represents the time required for an exoenzyme to supply a mass of C, N, or P equal to the total mass required to synthesise the exoenzyme in question. Finally, the amount of resource available to each unit volume of organism is determined by the relative digestion radius $\kappa$. Full details of the parameters and set of equations used in the model are given in the Supplementary Note 1.

We assume that the metabolic core of each class of organism has the same density of C, N, and P, which remain fixed in the simulations shown here (C_I_ = 0.165 g ml^−1^, N_I_ = 0.032 g ml^−1^, and P_I_ = 0.005 g ml^−1^). All organisms also have a fixed carbon use efficiency (CUE) of 0.5, defined as the ratio between the C contained in an organism and its exoenzymes, compared to the total C consumed, including the C lost in respiration. The final common parameter is the maximum rate of resource use, $\lambda$, set at 0.3 g ml^−1^ h^−1^ for all organisms, which constrains the maximal mass of resource any cell can use for each unit time and volume to a reasonable biological limit.

Each class of organism, apart from immobile unicells, has one additional parameter that is specific to their characteristic mode of capturing resource. Thus, autolytic cells have a recycling efficiency $\varepsilon = 0.5$ which sets the fraction of C, N, and P that is recouped by autolysis. Motile cells include the cost of motility $\alpha = 0.02$, which is the mass of C and N required to synthesise motile apparatus, relative to core demand for C and N. Fungal osmotrophs include a transport cost $\beta = 0.1$, defined as the mass of C and N being transported (and therefore not active), relative to the total C and N in exoenzymes and the fungal core.

To optimise the rate of growth in any given resource environment, immobile cells can only modulate the relative mass of exoenzymes secreted by each unit volume of organism, represented by the variable $x$. For any given set of model parameters, and any given value of $x$, we can calculate both the mass of resource required to grow a unit volume of organism, and the supply rate of resource for each unit volume of organism. We can therefore calculate the maximal specific growth rate $\eta$ for individual growing immobile cells (Supplementary Note 2). However, if only a fraction of the cells in the colony have access to the nutrients needed for growth, the apparent growth rate of the whole colony $\mu$ will be significantly smaller than the specific growth rate of individual growing cells $\eta$. For any given set of model parameters, we can computationally identify the value of $x$ that maximises the apparent growth rate $\mu$ (Supplementary Note 3).

We follow the same approach for colonies of autolytic cells (Supplementary Note 4), motile cells (Supplementary Note 5), and fungal hyphae (Supplementary Note 6), to identify which class of organism can most rapidly colonise any given resource. The value of the organism-specific parameters can be varied, and we discuss the most appropriate values in Supplementary Note 7.

Our main focus is the initial competition to capture resource, so we model the early stages of colonisation when colonies undergo exponential growth, before crowding effects limit growth. Nevertheless, we consider the impact of crowding in Supplementary Note 8, when apparent growth rates become time-varying.

We also consider the impact of resource accessibility (Supplementary Note 9), by imposing an additional environmental parameter $\delta$. The resource accessibility, $\delta$, is the minimum fraction of available C that must be digested in order to access the available N, to reflect that N may be embedded within C-rich polymers.

Finally, we comment on our original assumption that all organisms can be modelled with the same C_I_, N_I_, and P_I_, and CUE in Supplementary Note 10.

The consequence of varying any of these parameters can be explored using the MATLAB GUI interface or MATLAB scripts that are provided in Supplementary Software 1.

### Reporting summary

Further information on research design is available in the Nature Research Reporting Summary linked to this article.

## Supplementary information

Supplementary Information

Reporting Summary

Peer Review

Description of Additional Supplementary Files

Supplementary Software 1

## Data Availability

The authors confirm that all relevant data are included and fully referenced in the paper and the Supplementary Information files.

## Code availability

All programs were written in MATLAB (2019b, The MathWorks, Natick, MA) and are provided as a MATLAB app or a Windows standalone executable program, along with an instruction manual, from the Supplementary Software 1 as a ZIP file or available for download from https://github.com/markfricker/Fungal_multicellularity.
