# Supplementary material for: A mechanistic explanation of the transition to simple multicellularity in fungi
Source: Nat Commun. 2020 May 22;11:2594. doi: 10.1038/s41467-020-16072-4 (PMC7244713; doi:10.1038/s41467-020-16072-4)
Supplement: Supplementary file 1 — Supplementary Information [file 41467_2020_16072_MOESM1_ESM.pdf]

A Mechanistic Explanation of the Transition to Simple Multicellularity in Fungi
Supplementary Information

Heaton et al.

Contents

1 Parameter values for internal C:N:P ratio, dry weight and carbon use efficiency 2

2 Specific growth rate of immobile cells 4

3 Apparent growth rate of colonies of immobile cells 6

4 Apparent growth rate of colonies of autolytic cells 9

5 Apparent growth rate of colonies of motile cells 11

6 Apparent growth rate of hyphal colonies 13

7 Appropriate parameter values 15

8 Temporal variation in apparent growth rate due to crowding 20

9 Constraints on the relative rate of C and N digestion due to substrate accessibility 23

10 Varying demand for C, N and P 26

Supplementary Note 1 Parameter values for internal C:N:P ratio, dry weight and carbon use efficiency

The parameters $\mathrm{C_I}$, $\mathrm{N_I}$ and $\mathrm{P_I}$ represent the total mass of C, N and P contained within a unit volume of organism, excluding any resource contained in exoenzymes, vesicles or motile machinery. The parameter $\mathrm{C_T}$ represents the total mass of C required for each unit volume of growth, including C that is lost through respiration due to any kind of metabolic activity (Supplementary Table 1). In reality, the amount of C, N and P required for each unit volume of growth will depend on numerous factors (see Supplementary Note 10), but for the sake of simplicity we have used the same values for all organisms under all growth conditions.

Typically, half the carbon used by an organism is allocated to synthetic machinery and exoenzymes, while the other half is lost through respiration [1], so $\mathrm{C_T} \approx 2\mathrm{C_I}$. However, carbon use efficiency (CUE) can be much lower than 50% when the available carbon is hard to digest [2]. The C:N:P ratios of terrestrial microbes vary by an order of magnitude [3, 4, 5, 6], but an internal molar C:N ratio of 6:1 and an N:P ratio of 14:1 is fairly typical. Thus, including the respiratory C requirement increases the overall C:N ratio that must be acquired for growth to 12:1.

The dry weight for each unit volume of fungal and bacterial cells also varies across environments and species, but values are typically in the range $0.1 - 0.34$ g ml^{-1} [7, 8]. If the dry mass density of cells is 0.2 g ml^{-1}, half the carbon initially acquired is lost in respiration, the overall C:N ratio of cells is 6:1 and the N:P ratio is 14:1, it follows that immobile cells require $\mathrm{C_T} = 0.33$ g ml^{-1}, $\mathrm{N_I} = 0.032$ g ml^{-1} and $\mathrm{P_I} = 0.005$ g ml^{-1}, where $\mathrm{C_T}$, $\mathrm{N_I}$ and $\mathrm{P_I}$ denote the mass of C, N and P required for each unit volume of growth (Supplementary Table 1). These are the values we have used to generate the figures in the Main Text and Supplementary Information. However, we note that changing the parameters $\mathrm{C_T}$, $\mathrm{N_I}$ and $\mathrm{P_I}$ produces qualitatively similar results, as growth rates depend on the ratio between $\mathrm{C_T}$ and $\kappa^2\mathrm{C_E}$ (and likewise for N and P), not absolute values.

Exoenzymes break down the local resource into low molecular weight, soluble molecules that can be taken up and used to fuel growth [9]. In our model it is convenient to let x denote a variable: the mass of C and N required to synthesise exoenzymes, relative to the mass of C and N required to synthesise the cell itself, both including the additional C respired to produce the energy required. As we are interested in identifying the maximal growth rate for each category of organism, we can assume that for all unicellular organisms, C, N and P digesting exoenzymes are released in proportion to the demand for C, N and

Supplementary Table 1: Definition and values of modelling parameters

Tunable Environmental Parameters	Symbol and Units	Interpretation
Supply of C, N and P	C_E, N_E, P_E	Grams of C, N and P for each millilitre of substrate, set by the C:N and N:P ratio of the resource, and the resource density in g ml^{-1}
Recalcitrance	τ hours	Time required for an exoenzyme to supply a mass of C, N or P equal to the total mass required to synthesise the exoenzyme in question
Relative digestion radius	κ	Cylindrical cells and hyphae digest resource over a distance κ times the cell radius
Resource accessibility	δ	Ratio of C that has to be digested to release each N, to reflect that N is embedded within C-rich polymers

Tuneable Organism Parameters	Symbol and Units	Interpretation
Core demand for C, N and P	$C_I = 0.165$ g ml^{-1}<br>$N_I = 0.032$ g ml^{-1}<br>$P_I = 0.005$ g ml^{-1}	Core demand for C, N and P for each unit volume of any organism.
Carbon Use Efficiency	CUE $= 0.5$	Ratio of C_I for growth to total C required (C_T), including respiration
Motility cost	$\alpha = 0.02$	Mass of C and N required to synthesise motile apparatus, relative to core demand for C and N
Vesicle transport cost	$\beta = 0.1$	Mass of C and N in vesicles, relative to the total C and N in exoenzymes and the fungal core
Recycling efficiency	$\epsilon = 0.5$	Fraction of C, N and P that is recouped by autolysis
Maximal rate of resource use	$\lambda = 0.3$ g ml^{-1} h^{-1}	Maximal mass of resource any cell can use for each unit time and volume

Model Variables	Symbol and Units	Interpretation
Relative density of exoenzymes	x	Mass of C and N used for exoenzymes relative to the core demand for C and N for unicells
Relative density of C, N or P exoenzymes	x_C, x_N, x_P	Mass of C and N used for C, N or P digesting exoenzymes relative to the core demand for C and N for fungi

Functions of x	Symbol and Units	Interpretation
Time to exhaust local resource	T, h	Time until one of C, N or P is locally exhausted
Time to exhaust C, N or P	T_C, T_N, T_P, h	Time until relevant element is locally exhausted
Specific growth rate of growing cell	η, h^{-1}	Rate of synthesis of cell biomass, for each unit of cell biomass, in the growing cells
Apparent growth rate	μ, h^{-1}	Volumetric rate of colonisation, for each unit volume colonised
Total limiting supply	Ω	Maximum number of daughter cells and their exoenzymes that can be synthesised, given the local supply of resource
Rate of resource use	Γ, g ml^{-1} h^{-1}	Total mass of resource used for each unit time and volume, in the growing cells

P, so that no resource is wasted producing exoenzymes that generate resource in excess of the organism's requirements.

Supplementary Note 2 Specific growth rate of immobile cells

In the model, immobile cells represent the simplest osmotrophic scenario and can only modulate the amount of exoenzymes secreted to optimise growth in any given resource environment. Changing the relative density of exoenzymes x changes the mass of C and N required for each unit volume of cell, but because exoenzymes do not contain P, changing x does not change the amount of P required for each unit volume of cell. Thus we assume that growing an immobile cell and its exoenzymes requires a mass $(1 + x)(\mathrm{C_T} + \mathrm{N_I}) + \mathrm{P_I}$ for each unit volume (see Main Text Fig. 3a). We also assume that C, N or P digesting exoenzymes that have access to the relevant resource take time τ to digest a mass of C, N or P equal to the total mass required to synthesise the exoenzyme in question. Thus the parameter τ reflects the difficulty of digesting the substrate.

In our model, all of the resources required by a growing organism are accounted for by the quantities $\mathrm{C_T}$, $\mathrm{N_I}$ and $\mathrm{P_I}$, and for any given relative density of exoenzymes x, we let $\Gamma(x)$ denote the rate of resource use for each unit volume of growing cell. Hence where digestion of the local substrate is the sole source of resource, we assume that $\Gamma(x)$ is equal to the rate of resource digestion for each unit volume of growing cell. Thus we assume that all resource is 'used' as soon it is digested. However, for all organisms, the rate of biomass synthesis is limited by the kinetics of transcription and translation. Thus for any relative density of exoenzymes x, and any supply rate of resource, we assume that $\Gamma(x) \leq \lambda$, where λ is a parameter set to equal 0.3 g ml^{-1} hour^{-1}. This constraint ensures that the smallest doubling time for any cell under any circumstance is $\frac{\log(2)}{\lambda}(\mathrm{C_T} + \mathrm{N_I} + \mathrm{P_I}) = 51$ mins, well below the maximum doubling time for prokaryotes or eukaryotes expected in the wild [10, 11, 12].

Since $(\mathrm{C_T} + \mathrm{N_I})x$ denotes the mass of C and N required to synthesise the exoenzymes released by a unit volume of cell, it follows from the definition of τ that $\frac{(\mathrm{C_T}+\mathrm{N_I})x}{\tau}$ is the mass digested for each unit time, for each unit volume of cell. Thus for immobile cells, where all the resource used is obtained by digesting the local substrate, the rate of resource use

$$\Gamma_\iota(x) = \min\left[\frac{(\mathrm{C_T} + \mathrm{N_I})x}{\tau}, \quad \lambda\right], \tag{1}$$

where the subscript ι is used to indicate that we are referring to the case of immobile cells.

By definition, the specific growth rate of an organism, η, is the rate of synthesis of biomass for each unit of biomass. Since Γ_ι is the total mass used for each unit time for each unit volume of cell, and $\mathrm{C_T} + \mathrm{N_I} + \mathrm{P_I}$ is the dry mass density of immobile cells, $\frac{\Gamma_\iota}{\mathrm{C_T}+\mathrm{N_I}+\mathrm{P_I}}$ is the total mass used for each unit time for each unit mass. It might thus seem that the specific growth rate of an immobile cell $\eta_\iota = \frac{\Gamma_\iota}{\mathrm{C_T}+\mathrm{N_I}+\mathrm{P_I}}$. However, only a fraction $\frac{\mathrm{C_T}+\mathrm{N_I}+\mathrm{P_I}}{(\mathrm{C_T}+\mathrm{N_I})(1+x)+\mathrm{P_I}}$ of the total mass required is used to synthesise cell biomass, as opposed to exoenzymes, and it is only the synthesis of cell biomass that contributes to specific growth rate. Hence the specific growth rate of a growing immobile cell η_ι is given by Supplementary Equation 2

$$\eta_\iota(x) = \frac{\Gamma_\iota(x)}{(\mathrm{C_T}+\mathrm{N_I})(1+x)+\mathrm{P_I}} \tag{2}$$

$$= \min\left[\frac{x}{\tau(1+x+\frac{\mathrm{P_I}}{\mathrm{C_T}+\mathrm{N_I}})},\quad \frac{\lambda}{(\mathrm{C_T}+\mathrm{N_I})(1+x)+\mathrm{P_I}}\right]. \tag{3}$$

More generally, the specific growth rate of an organism will equal $\frac{\Gamma}{D}$, where Γ is the rate of resource use for each unit volume and D is the total mass used to grow a unit volume, including the mass used to synthesise exoenzymes. Also note that where $V(t)$ denotes the volume of an immobile cell at time t, the mass of the cell is $(\mathrm{C_T}+\mathrm{N_I}+\mathrm{P_I})V(t)$, the mass of the exoenzymes supplying the cell is $(\mathrm{C_T}+\mathrm{N_I})xV(t)$, and the rate of resource use by the cell is $\Gamma_\iota(x)V(t)$. Since resource is used to synthesise cell biomass and exoenzymes, it follows that the growth rate of an immobile cell is

$$\frac{dV}{dt} = \frac{\Gamma_\iota(x)V(t)}{(1+x)(\mathrm{C_T}+\mathrm{N_I})+\mathrm{P_I}}. \tag{4}$$

Hence the specific growth rate η is equal to the rate of growth for each unit volume of growing cell, as can be deduced from the assumption that daughter cells have the same density as parent cells.

In the absence of a maximal rate of resource use λ, the specific growth rate $\eta_\iota(x)$ is a monotonically increasing function of x. However, larger values of x require greater quantities of C and N for each unit volume, so increasing the amount of resource allocated to exoenzymes decreases the number of daughter cells that can be synthesised using the fixed amount of local resource. Also note that it cannot be optimal to allocate so much resource to exoenzymes that $x > \frac{\lambda\tau}{\mathrm{C_T}+\mathrm{N_I}}$, as in that case the exoenzymes would generate resource at a rate that exceeds the cell's synthetic capacity. As there is a cost to synthesising

exoenzymes, it follows that the maximal rate of colonisation for each unit volume colonised must arise for some value of $x \leq \frac{\lambda\tau}{\mathrm{C_T+N_I}}$.

Supplementary Note 3 Apparent growth rate of colonies of immobile cells

Our focus is the competition to capture resource, so we are interested in comparing the volume of resource captured by organisms for each unit time. The volume colonised for each unit time will increase in proportion to the size of the colony, and we define the apparent growth rate of a colony as the volume of resource that is colonised for each unit time, for each unit volume that has already been colonised. If all resource is colonised by growth, as opposed to migration, and cells persist even after they have exhausted the local supply of nutrients, the apparent growth rate μ is simply equal to the mean specific growth rate of the cells in the colony. Crucially, if only a fraction of the cells in the colony have access to the nutrients needed for growth, the apparent growth rate of the colony μ will be significantly smaller than the specific growth rate of individual growing cells η. Cells that have exhausted the local supply of resource cannot grow, and if a fraction f of the cells in the colony grow at rate η and the remaining cells do not grow at all, $\mu = f\eta$.

When the time taken to exhaust the local supply of resource is T, and the volume of colonised resource at time t is $V(t)$, a volume $V(t) - V(t-T)$ is able to support growth with a specific growth rate η. Hence we find that

$$\frac{dV}{dt} = [V(t) - V(t-T)]\eta, \quad \text{and} \tag{5}$$

$$\mu(t) = \frac{\frac{dV}{dt}}{V} = \left[1 - \frac{V(t-T)}{V(t)}\right]\eta. \tag{6}$$

If the ratio $\frac{V(t-T)}{V(t)}$ changes over time, the apparent growth rate of the colony changes over time, even if the specific growth rate of growing cells η remains constant (see Supplementary Note 7). However, when the colony grows exponentially, with volume $V(t) = V(0)e^{\mu t}$, the fraction of the colony that has exhausted the local supply of resource is a constant $\frac{V(t-T)}{V(t)} = \frac{e^{\mu(t-T)}}{e^{\mu t}} = e^{-\mu T}$. In that case a fraction $1 - e^{-\mu T}$ of the colony can grow at rate η, and a fraction $e^{-\mu T}$ cannot grow at all. Hence the apparent growth rate of a colony of immobile cells is

$$\mu_\iota(x) = \eta_\iota(x)\Big[1 - \exp\big(-\mu_\iota(x)T_\iota(x)\big)\Big], \tag{7}$$

where $T_\iota(x)$ denotes the time taken to exhaust local supply of resource, for any given x

and any given set of tunable environmental parameters C_E , N_E, P_E, the digestion radius κ and recalcitrance parameter τ (see Supplementary Table 1).

The time taken to exhaust the local supply of resource (T) depends on the rate of resource use, and the quantity of resource that is available for consumption. Exoenzymes are composed of C and N, and do not contain P, so for a colony of immobile unicellular organisms, we let the total limiting supply

$$\Omega_\iota(x) = \min\left[\frac{\kappa^2 C_E}{C_T(1+x)}, \quad \frac{\kappa^2 N_E}{N_I(1+x)}, \quad \frac{\kappa^2 P_E}{P_I}\right]. \tag{8}$$

Note that $\Omega_\iota(x)$ is equal to the maximum number of daughter cells and their associated exoenzymes that can be synthesised, for any given x and any given set of tunable environmental parameters C_E , N_E, P_E, κ and τ. Also note that the time taken to exhaust the limiting nutrient is equal to the time it takes a growing cell to grow Ω daughter cells. The time taken for a unit volume of growing cell to grow a unit volume is $\frac{1}{\eta}$, so for immobile cells, the time to exhaust the local supply of resource is

$$T_\iota(x) = \frac{\Omega_\iota(x)}{\eta_\iota(x)}. \tag{9}$$

The system of Supplementary Equations 2, 7, 8 and 9 can be solved computationally to yield μ, for any given x and any given set of tunable environmental parameters C_E , N_E, P_E, κ and τ (Supplementary Figure 1(a))

We also note that Supplementary Equations 7 and 9 imply that

$$\mu_\iota(x) = \eta_\iota(x)\Big[1 + \frac{W_0(-\Omega_\iota \exp(-\Omega_\iota))}{\Omega_\iota}\Big], \tag{10}$$

where W_0 is the principle branch of the Lambert W functional [13]. Thus the apparent growth rate of a colony of immobile cells is proportional to the specific growth rate of the individual growing cells η, and it also proportional to a monotonically increasing function of the total limiting supply Ω. All organisms have a low growth rate when τ is large, but immobile cells have a particularly low growth rate when the local resource is only sufficient to synthesise a small number of daughter cells and their exoenzymes (Ω_ι close to 1). For example, when Ω_ι is 1.1, 1.5 or 2, only 17%, 58% or 80% of the colony is able to grow, and the apparent growth rate of the colony is reduced by the same proportion (see Supplementary Figure 5(b)).

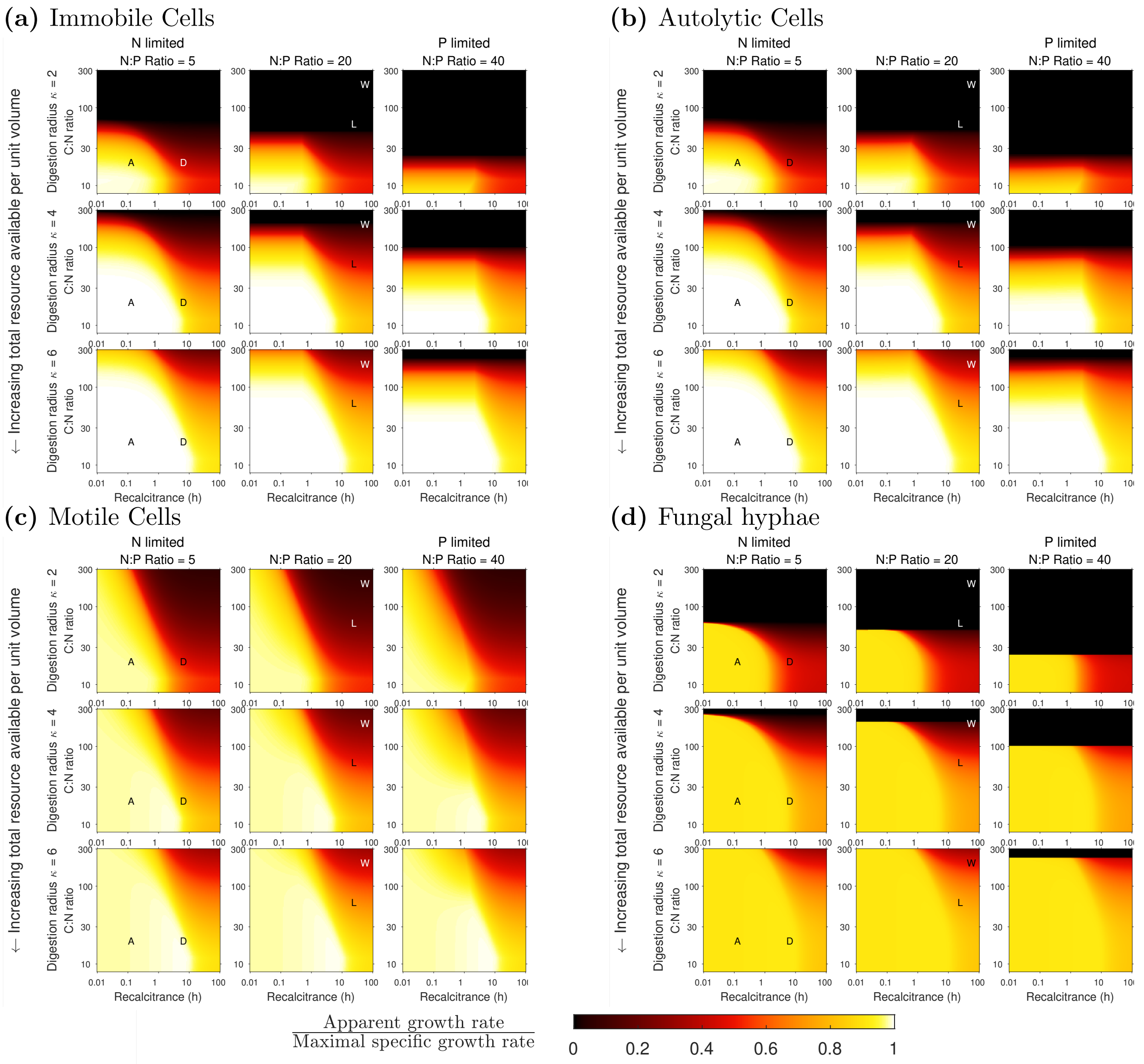


```
W = wood    (C:N:P = 4000:20:1, Tau =  40 h)
L = litter  (C:N:P = 1250:20:1, Tau =  20 h)
D = dung    (C:N:P = 100:5:1,   Tau =   5 h)
A = agar    (C:N:P = 100:5:1,   Tau = 0.1 h)
```

Supplementary Figure 1: Reduction in colony growth rate compared to the maximum specific growth rate as resources become limited. The scale of growth reduction due to the exhaustion of local resource depends on resource availability and the recalcitrance of substrate. For immobile (a), autolytic (b), motile (c) and fungal colonies (d), the colour of each pixel shows the ratio between the apparent growth rate of the colony, and the maximal specific growth rate of an individual, immobile cell. All growth rates depend on the recalcitrance of the substrate, but only the apparent growth rates of colonies depend on the total limiting supply of resource. Hence pixel colour can be interpreted as the extent to which growth rates are reduced due to the effect of resource becoming exhausted, plus the the additional costs of motile apparatus or internal transport. We assume the substrate has a dry mass of 0.5 g ml^{-1}, and it can be consumed over 2, 4 or 6 cell radii (rows), with an N:P ratio of 5:1, 20:1 or 40:1 (columns). Within each plot, the recalcitrance (x-axis) and C:N ratio (y-axis) are varied over a wide but physiologically relevant range, shown on log scales.

Supplementary Note 4 Apparent growth rate of colonies of autolytic cells

We assume that once an autolytic cell exhausts the local resource a fraction ϵ of the resource used to grow the cell becomes instantly and freely available to the remaining cells, though any resource used to synthesise exoenzymes cannot be recouped. This represents a simple model for cells undergoing autolysis or more controlled programmed cell death (PCD). Whilst these destructive processes lower the fitness of individual cells, there is increasing evidence that it provides an advantage to neighbouring cells via recycling resources, reducing stress responses or enhancing biofilm formation [14, 15, 16]. Furthermore, computer simulations show that recycling alone can confer a benefit for PCD [17]. It is noteworthy that in the context of degradation of immobile resources, it is likely that there is a higher probability that neighbours are related kin, which might support adaptation at a group level [18]. As resource can be obtained from autolysis as well as the digestion of local resource, the rate of resource use for each unit volume (Γ) is no longer described by Supplementary Equation 1. To determine the relationship between the relative density of exoenzymes x and the rate of resource use Γ in the case of autolytic cells, we note that if the time that elapses from initial growth until autolysis is T, the number of cells initiating autolysis at time t is equal to the number of cells that were newly grown at time $t - T$. It follows that if the volume that has been colonised at time t is $e^{\mu t}$, the volume of growing cells is $e^{\mu t}(1 - e^{-\mu T})$, the volumetric rate of growth is $\mu e^{\mu t}$ and the volume of biomass lost by autolysis for each unit time is $\mu e^{\mu(t-T)}$. As each unit volume that undergoes autolysis releases a total $\epsilon(\mathrm{C_T} + \mathrm{N_I} + \mathrm{P_I})$ of resource, the mass of resource obtained by autolysis for each unit volume of growing cell is

$$\frac{\epsilon(\mathrm{C_T} + \mathrm{N_I} + \mathrm{P_I})\mu e^{\mu(t-T)}}{e^{\mu t}(1 - e^{-\mu T})} = \frac{\epsilon\mu(\mathrm{C_T} + \mathrm{N_I} + \mathrm{P_I})}{e^{\mu T} - 1}. \tag{11}$$

The remaining resource that the colony requires must be obtained by digesting the local resource, and as specified earlier, exoenzymes supply resource at a rate $\frac{x(\mathrm{C_T}+\mathrm{N_I})}{\tau}$ for each unit volume of cell. We also assume that, as in the case of immobile cells, resource is used as soon as it is digested, and there is a maximum rate of resource use for each unit volume specified by λ. It follows that for autolytic cells, the rate of resource use for each unit volume

$$\Gamma_a(x) = \min\left[\frac{(\mathrm{C_T} + \mathrm{N_I})x}{\tau} + \frac{\epsilon\mu(\mathrm{C_T} + \mathrm{N_I} + \mathrm{P_I})}{e^{\mu T} - 1}, \quad \lambda\right], \tag{12}$$

where μ denotes the apparent growth rate of the colony and T denotes the lifespan of cells (that is, the time taken to exhaust the local supply of the most limiting nutrient).

As in the case of immobile cells, the specific growth rate η_a is related to Γ_a by Supplementary equation 13

$$\eta_a(x) = \frac{\Gamma_a(x)}{(\mathrm{C_T} + \mathrm{N_I})(1 + x) + \mathrm{P_I}}. \tag{13}$$

The time taken for a unit volume of cell to grow a unit volume is $\frac{1}{\eta_a}$, so it follows that the time to exhaust the local supply is

$$T_a(x) = \frac{\Omega(x)}{\eta_a(x)}, \tag{14}$$

where $\Omega(x)$ is specified by Supplementary Equation 8.

Only a fraction $1-e^{-\mu_a T_a}$ of the colonised volume contains growing cells, so the apparent growth rate of the colony μ_a satisfies the implicit equation

$$\mu_a(x) = \eta_a(x)\Big[1 - \exp\big(-\mu_a(x)T_a(x)\big)\Big]. \tag{15}$$

Supplementary Equation 15 can be seen as an equation system, built from Supplementary Equations 12, 13 and 14. For a given value of x, and any given set of tunable environmental parameters $\mathrm{C_E}$, $\mathrm{N_E}$, $\mathrm{P_E}$, κ and τ, this system of equations can be solved computationally, by estimating $\mu_a(x)$ and $\Gamma_a(x)$ then using Supplementary Equations 12 - 15 to refine that estimate iteratively (Supplementary Figure 1(b)). The apparent growth rate also depends on the fraction of resource that is recouped by autolysis, ϵ, but our results are qualitatively very similar if ϵ is varied. In particular, we note that there are environments where fungi and motile cells grow significantly faster than autolytic cells even in the extreme case of 'perfect' recycling $\epsilon = 1$. Also note that as before, it cannot be optimal to have a relative density of exoenzymes such that

$$\frac{x(\mathrm{C_T} + \mathrm{N_I})}{\tau} + \frac{\epsilon\mu(\mathrm{C_T} + \mathrm{N_I} + \mathrm{P_I})}{e^{\mu T} - 1} > \lambda, \tag{16}$$

as in that case the exoenzymes would generate resource at a rate that exceeds the cell's synthetic capacity, and there is a cost to synthesising exoenzymes.

Supplementary Note 5 Apparent growth rate of colonies of motile cells

Many unicellular organisms are motile and this allows them to continuously forage for resources. These are the only class of organisms that can survive when there is insufficient local resource to actually allow cell doubling, typical for low quality resources or where the resource volume is limited. By assumption, motile cells have the same core biosynthetic machinery as immobile cells, but there is an additional cost of C and N needed to synthesise the apparatus needed for motility. This additional cost is represented by the parameter α, which is equal to the mass of C and N required to synthesise and operate the motile apparatus, relative to the mass of C and N required to synthesise the other parts of the cell (see Main Text Fig. 3b). Thus growing a motile cell requires a mass $(1+\alpha+x)(\mathrm{C_T}+\mathrm{N_I})+\mathrm{P_I}$ for each unit volume, where as before, x is variable that represents the density of exoenzymes, and $x(\mathrm{C_T}+\mathrm{N_I})$ is the mass of C and N used for exoenzymes for each unit volume of cell.

As specified earlier, exoenzymes supply resource at a rate $\frac{x(\mathrm{C_T}+\mathrm{N_I})}{\tau}$ for each unit volume of cell, resource is used as soon as it is digested, and there is a maximum rate of resource use for each unit volume λ. It follows that in the case of motile cells the rate of resource use for each unit volume is

$$\Gamma_m(x) = \min\left[\frac{(\mathrm{C_T}+\mathrm{N_I})x}{\tau}, \quad \lambda\right]. \tag{17}$$

The total mass required for each unit volume is $(1+\alpha+x)(\mathrm{C_T}+\mathrm{N_I})+\mathrm{P_I}$, so it follows that the specific growth rate of motile cells is

$$\eta_m(x) = \frac{\Gamma_m(x)}{(1+\alpha+x)(\mathrm{C_T}+\mathrm{N_I})+\mathrm{P_I}}. \tag{18}$$

In the case of motile cells, we are interested in the number of daughter cells and their associated exoenzymes that can be synthesised, while still leaving sufficient C and N for the exoenzymes that will be needed as soon as the parent cell migrates, to allow for further resource acquisition. The exoenzymes that will be released in a new location require $\mathrm{C_T}x$ and $\mathrm{N_I}x$ of C and N respectively, so it follows that for motile cells, the limiting local supply

$$\Omega_m(x) = \min\left[\frac{\kappa^2\mathrm{C_E}-\mathrm{C_T}x}{\mathrm{C_T}(1+\alpha+x)}, \frac{\kappa^2\mathrm{N_E}-\mathrm{N_I}x}{\mathrm{N_I}(1+\alpha+x)}, \frac{\kappa^2\mathrm{P_E}}{\mathrm{P_I}}\right]. \tag{19}$$

The time taken for a unit volume to grow a unit volume is $\frac{1}{\eta_m}$, so the duration of the

growth phase of mobile cells is $\frac{\Omega_m}{\eta_m}$. By definition, exoenzymes supply a mass of resource equal to the mass required to synthesise themselves over a time-scale τ, so the time taken to synthesise the exoenzymes that will be needed after migration is τ. Cells migrate after they have completed the growth phase and synthesised the necessary replacement exoenzymes. Hence the number of cells migrating at time t is equal to the number of cells arriving in new territory at time $t - \frac{\Omega}{\eta} - \tau$. It follows that the apparent growth rate due to migration (rate of colonisation by migration for each unit volume colonised) is

$$\mu_m(x)\frac{\exp\left[\mu_m(x)(t - \frac{\Omega_m(x)}{\eta_m(x)} - \tau)\right]}{\exp\left[\mu_m(x)t\right]} = \mu_m(x)\exp\Big[-\mu_m(x)\Big(\frac{\Omega_m(x)}{\eta_m(x)} + \tau\Big)\Big], \tag{20}$$

where $\mu_m(x)$ denotes the apparent growth rate due to growth and migration for any given x and any given set of tunable environmental parameters C_E , N_E, P_E, κ and τ. As the duration of the growth phase is $\frac{\Omega}{\eta}$, a fraction $\exp[-\mu\frac{\Omega}{\eta}]$ of the colonised volume no longer supports growth. Hence the apparent growth rate due to growth and migration must satisfy Supplementary Equation 21

$$\mu_m(x) = \eta_m(x)\Big(1 - \exp\Big[-\mu_m(x)\frac{\Omega_m(x)}{\eta_m(x)}\Big]\Big) + \mu_m(x)\exp\Big[-\mu_m(x)\Big(\frac{\Omega_m(x)}{\eta_m(x)} + \tau\Big)\Big]. \tag{21}$$

It follows that for motile cells, the apparent growth rate $\mu_m(x)$ must satisfy the implicit Supplementary Equation 22

$$\mu_m(x) = \eta_m(x)\frac{\exp\left[\mu_m(x)\frac{\Omega(x)}{\eta(x)}\right] - 1}{\exp\left[\mu_m(x)\frac{\Omega(x)}{\eta(x)}\right] - \exp[-\mu_m(x)\tau]}, \tag{22}$$

for any given x and any given set of tunable environmental parameters C_E , N_E, P_E, κ and τ. The apparent growth rate $\mu_m(x)$ also depends on the parameter α, but results are qualitatively similar if we vary this parameter. As the flagella of *Salmonella enterica* is reported to account for 2% of biosynthetic energy expenditure [19], it is reasonable to restrict our attention to values of α close to 0.02.

Supplementary Equation 22 is an implicit equation for the apparent growth rate of a colony, and it can be solved computationally by finding an approximate solution for μ_m and iteratively refining it (Supplementary Figure 1(c)). Note that μ_m is close to the specific growth rate of individual cells η_m when Ω_m is significantly larger than one, or τ is small compared to the doubling time of each cell. In the former case the bulk of colonisation is achieved by growth, as many cell duplications can occur before cells need to migrate. In

the latter case exoenzymes will account for a small fraction of the biomass, and the need to spend time and resource synthesising exoenzymes prior to migration is a small burden. Also note that as in the case of immobile cells, the maximal rate of colonisation will always occur when $x \leq \frac{\lambda\tau}{C_T+N_I}$, because it cannot be efficient to synthesis so many exoenzymes that the supply rate of resource exceeds the cell's maximal synthetic capacity.

Supplementary Note 6 Apparent growth rate of hyphal colonies

Hyphal organisms have the same costs for core biosynthetic machinery as immobile cells, but face additional costs of C and N as the exoenzymes and cell wall materials that they synthesise must be transported to the growth front, and when those materials are in transit, they are not yet helping liberate new food resource. We assume that exoenzymes are predominantly secreted at the hyphal tips [20], which has been demonstrated experimentally for a number of exoenzymes [21, 22]. Furthermore, spatially explicit gene expression studies support this view, with genes involved in protein synthesis and exoenzyme production expressed at the colony periphery, whilst genes coding for transporters and permeases are located at the colony centre [23]. Nevertheless, if exoenzymes were secreted all over the mycelium, the amount in transit would be lower and the cost associated with hyphal transport (β) would be reduced. However, each enzyme would have access to less total resource over its lifespan, as colonised resource would already be partially digested, so secreting exoenzymes at locations distal to the tip would be less efficient.

We assume that the C and N being transported weighs β, relative to the weight of C and N in both exoenzymes and the fungal core (see Main Text Fig. 3c). Thus growing a fungus (or other hyphal organism) requires a mass $(1 + x)(1 + \beta)(C_T + N_I) + P_I$ for each unit volume, where x is the relative density of exoenzymes. Also note that although we account for the cost of transport by referring to the mass of material contained within vesicles, we do not need to make any assumptions about whether the transported material is membrane bound, or indeed whether it comprises exoenzymes per se, or amino acid precursors. It is sufficient to note that mycelial organisms transport material over longer distances than unicellular organisms, and long distance transport carries a cost reflected by the parameter β.

One interesting aspect of transport that we have not yet modelled is the transport pathway itself. The ancestral state still present in lower fungi like the Zygomycota is thought to comprise aseptate coenocytic hyphae, that would conform well to the model simulation. However, cell compartments in ascomycetes and basidiomycetes are separated by septal

pores, which provide cytoplasmic continuity when open, but can be rapidly blocked to prevent leakage if hyphae are damaged [24]. Numerous studies have also shown that there is an increasing probability of septal pore closure during normal development moving distal from the hyphal tips through the peripheral growth zone [25, 26, 24, 27, 28, 29], which would impact on the long-distance transport modelled here. Equally, there is some evidence that pore closure is dynamic and dependent on the resource environment [29], and that small radiolabelled solute molecules may still move selectively through hyphae even with closed septa [28]. There is also strong evidence from radiotracer studies for extensive, rapid long-distance transport through mycelial networks over centimetres [30, 31, 32, 33, 34, 35], although the precise pathway is unknown [36].

We let Γ_f denote the total mass used for each unit time for each unit volume of fungus, and in the case of hyphal organisms, this is the rate of resource use for each unit volume for the whole colony, not the rate of resource use for each unit volume for only the growing cells. As we assume that the density of daughter hyphae is equal to the density of the parents, it follows that for fungi, the specific growth rate (rate of synthesis of fungal biomass for each unit of fungal biomass) and the apparent growth rate (rate of colonisation for each unit volume colonised) are equal to one another. Hence for any given relative rate of resource use Γ_f, the apparent growth rate of a fungal colony is

$$\mu_f(x) = \frac{\Gamma_f}{(\mathrm{C_T} + \mathrm{N_I})(1+\beta)(1+x) + \mathrm{P_I}}. \tag{23}$$

In the case of fungi, the relationship between the rate of resource use, Γ_f, and the relative density of exoenzymes, x, is complicated by the fact that we need to keep track of the rate of digestion, and the time to exhaust the local supply, of each of the different elements. This contrasts with the other types of organism, which are simply constrained by a single, limiting resource. We let x_C, x_N and x_P denote the mass of C, N and P digesting exoenzymes for each unit volume of fungus, relative to $\mathrm{C_T}+\mathrm{N_I}$, so we have $x_\mathrm{C}+x_\mathrm{N}+x_\mathrm{P} = x$. Given any relative density of exoenzymes x, the fraction of resource used that is C, N or P is

$$\bar{\mathrm{C}} = \frac{\mathrm{C_T}(1+x)(1+\beta)}{(\mathrm{C_T} + \mathrm{N_I})(1+x)(1+\beta) + \mathrm{P_I}} \tag{24}$$

$$\bar{\mathrm{N}} = \frac{\mathrm{N_I}(1+x)(1+\beta)}{(\mathrm{C_T} + \mathrm{N_I})(1+x)(1+\beta) + \mathrm{P_I}} \quad \text{and} \tag{25}$$

$$\bar{\mathrm{P}} = \frac{\mathrm{P_I}}{(\mathrm{C_T} + \mathrm{N_I})(1+x)(1+\beta) + \mathrm{P_I}} \quad \text{respectively.} \tag{26}$$

If the local resource did not become exhausted, a rate of resource use for each unit volume Γ_f requires a relative density of exoenzymes $x = \frac{\tau\Gamma_f}{\mathrm{C_T+N_I}}$, with

$$x_{\mathrm{C}} = \frac{\tau\Gamma_f\bar{\mathrm{C}}}{\mathrm{C_T + N_I}}, \quad x_{\mathrm{N}} = \frac{\tau\Gamma_f\bar{\mathrm{N}}}{\mathrm{C_T + N_I}} \quad \text{and} \quad x_{\mathrm{P}} = \frac{\tau\Gamma_f\bar{\mathrm{P}}}{\mathrm{C_T + N_I}}. \tag{27}$$

However, because resource does become exhausted, we require additional exoenzymes to compensate for the fact that some parts of the colony have exhausted their local supplies. Hence to calculate the values of x_{C}, x_{N} and x_{P} needed to maintain a given rate of resource use Γ_f, we need to know the time taken to exhaust the local resource. Since the mass of C available is $\kappa^2\mathrm{C_E}$ for each unit volume, and the mass digested for each unit time and unit volume is $\frac{x_{\mathrm{C}}(\mathrm{C_T+N_I})}{\tau}$, it follows that the time taken to exhaust the local supply of C is

$$T_{\mathrm{C}} = \frac{\kappa^2\mathrm{C_E}\tau}{x_{\mathrm{C}}(\mathrm{C_T + N_I})}, \tag{28}$$

and a similar argument holds for N and P.

When the apparent growth rate of the colony is μ, only a fraction $1 - e^{-\mu T_{\mathrm{C}}}$ of the colony is able to supply C. It follows that when the relative rate of resource use for each unit volume is Γ_f,

$$x_{\mathrm{C}} = \frac{\tau\bar{\mathrm{C}}\Gamma_f}{(1 - \exp[-\mu_f T_{\mathrm{C}}])(\mathrm{C_T + N_I})}, \tag{29}$$

and likewise for N and P. We can solve the system of Supplementary Equations 23, 24, 28 and 29 for any given value of Γ_f and any given set of tunable environmental parameters $\mathrm{C_E}$, $\mathrm{N_E}$, $\mathrm{P_E}$, κ and τ. We do this by estimating the corresponding values for μ_f, x_{C}, x_{N} and x_{P}, and iteratively refining those solutions. We can therefore find the value of Γ_f, and the values of x_{C}, x_{N} and x_{P}, which maximise the apparent growth rate μ_f (Supplementary Figure 1(d)).

Supplementary Note 7 Appropriate parameter values

The parameter ϵ represents the fraction of resource within nutrient deprived cells that can be recouped by the colony through the process of autolysis. In the absence of data we have chosen to use a large value $\epsilon = 0.5$, but our results are qualitatively very similar if we vary ϵ. In particular, there are regions of parameter space where fungi outperform autolytic cells even when $\epsilon = 1$ (see Supplementary Figure 2).

The parameter α represents the amount C and N required to synthesise the apparatus

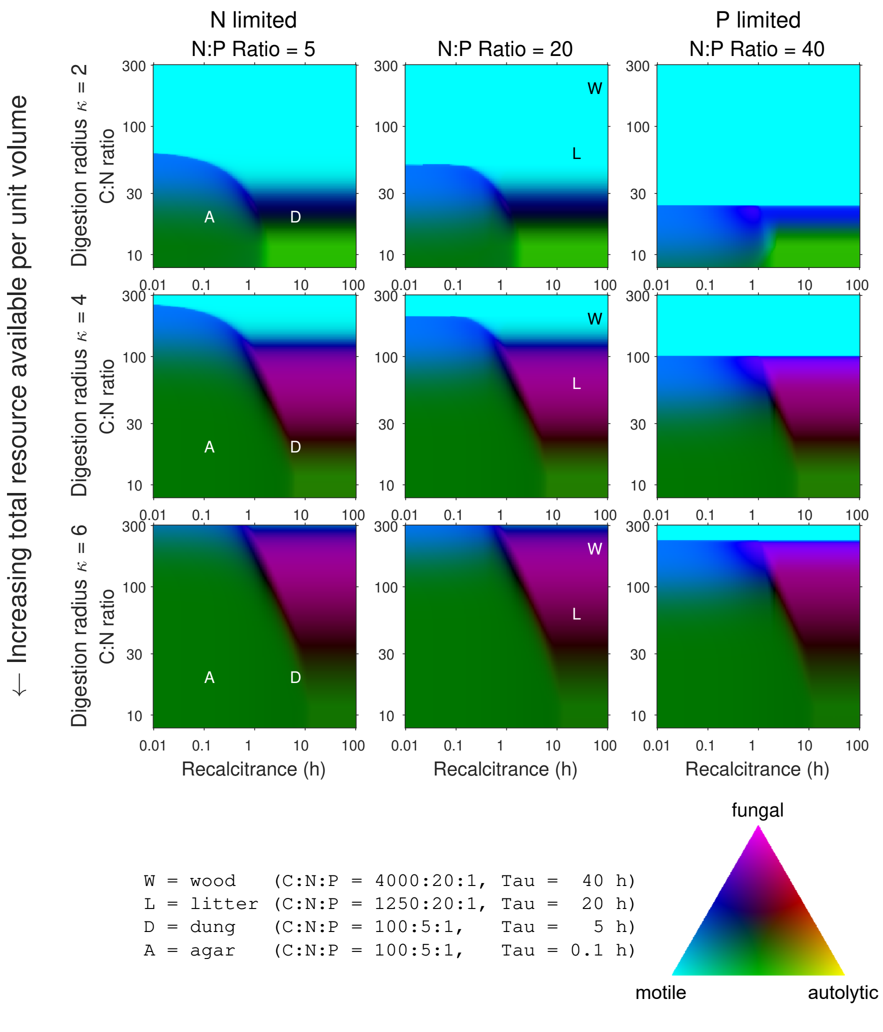

Supplementary Figure 2: Relative growth rates of different classes of organisms when only fungi incur additional costs. In some environments, fungi can grow faster than motile or autolytic cells even under the extreme case where there is no cost to synthesising motile apparatus, and autolysis recoups 100% of the cost of synthesis. Each panel indicates relative performance of each category of organism as the recalcitrance (x-axis) and C:N ratio (y-axis) are varied over a wide but physiologically relevant range, shown on log scales. Results are shown after the fastest growing colony increased in size by a factor of 1000, and the colour in each pixel is proportional to the increase in size of a colony of fungi (magenta), motile cells (cyan) and autolytic cells (yellow). To produce these plots we assume there is no cost to synthesising motile apparatus ($\alpha = 0$) and autolysis recoups 100% of the cost of cell synthesis ($\epsilon = 1$), though the cost of synthesising exoenzymes cannot be recouped. Rows correspond to increasing amount of resource availability as κ is varied over 2, 4 to 6 cell radii (see Main Text Fig. 3a). Columns correspond to varying N:P ratios set to 5:1, 20:1, and 40:1. The substrate has a total dry mass of 0.5 g ml^{-1} in all cases.

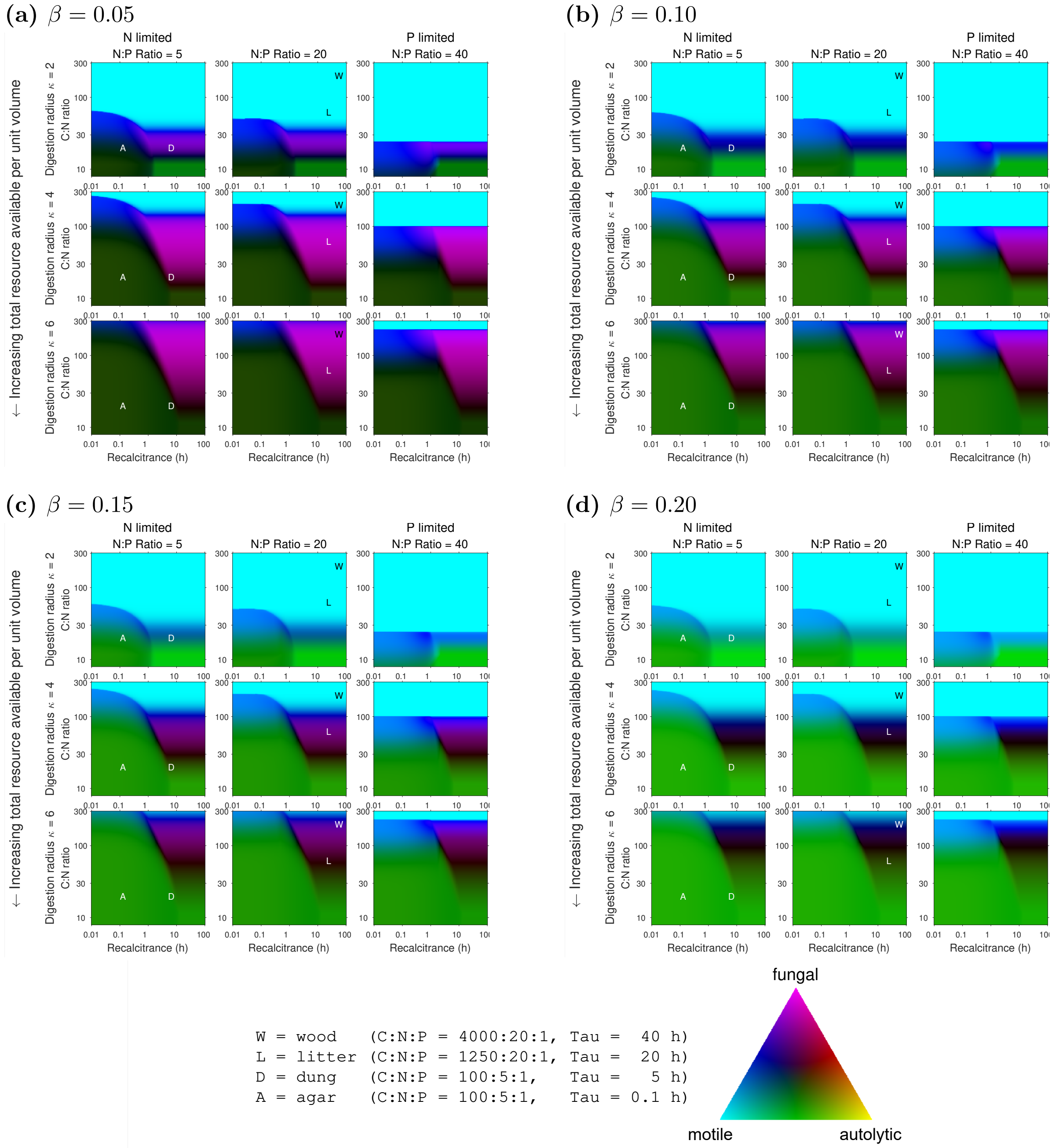

Supplementary Figure 3: The impact of varying internal transport costs on hyphal competitiveness. The scale of hyphal advantage over mobile or autolytic cells depends on resource availability, recalcitrance and internal transport cost β. Each panel indicates relative performance of each category of organism as the recalcitrance (x-axis) and C:N ratio (y-axis) are varied over a wide but physiologically relevant range, shown on log scales. Rows correspond to increasing amount of resource availability as κ is varied over 2, 4 and 6 cell radii. Columns correspond to varying N:P ratios set to 5:1, 20:1 and 40:1. The effect of varying the cost of internal transport β can be seen by comparing panels **(a)**-**(d)**, where $\beta = 0.05$, 0.1, 0.15 and 0.2 respectively. Note that the hyphal advantage over motile cells almost disappears when $\beta = 0.2$, when motile cells dominate. However, cell migration is only possible when the environment is sufficiently water saturated [37, 38].

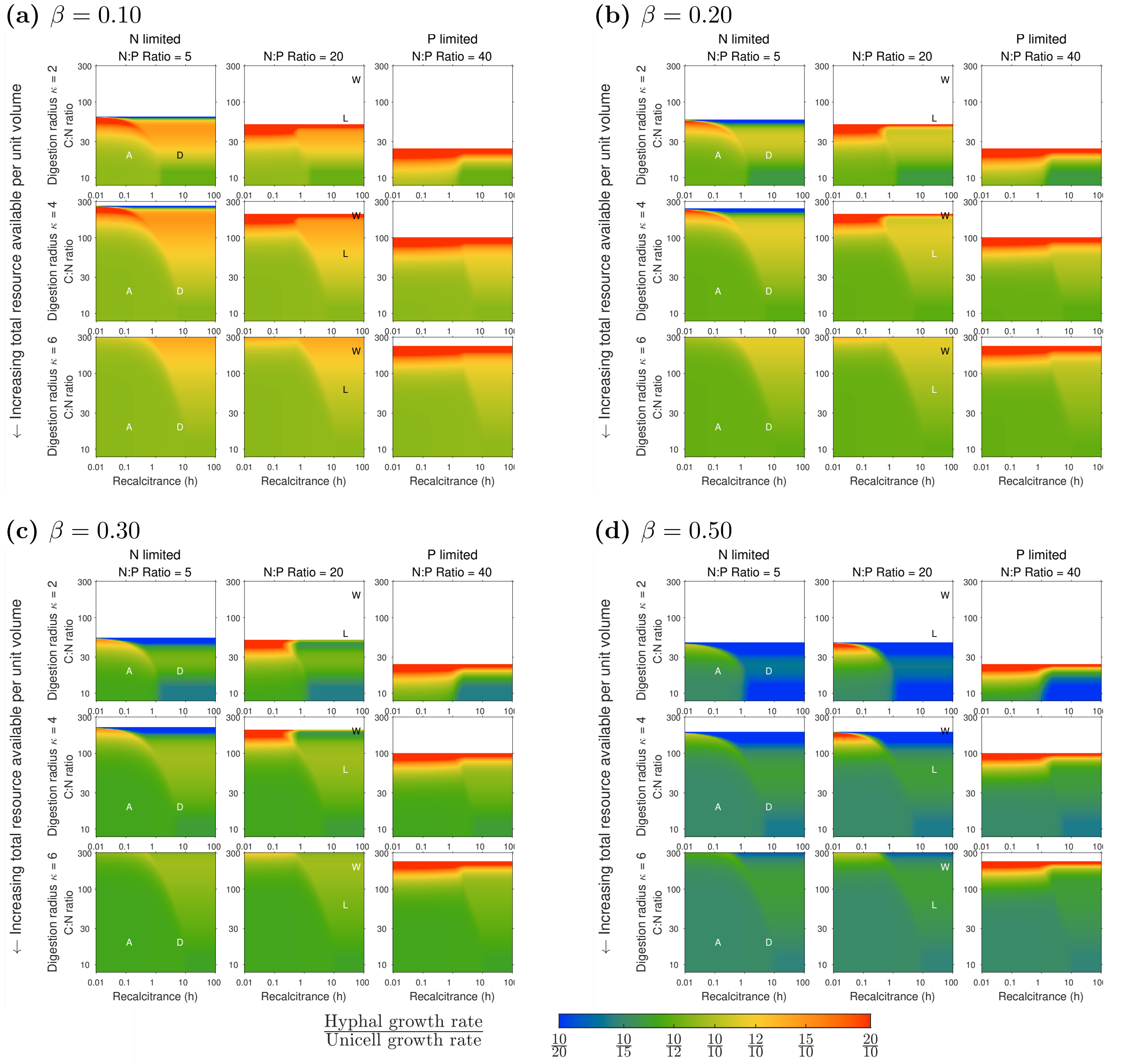


```
W = wood    (C:N:P = 4000:20:1, Tau =  40 h)
L = litter  (C:N:P = 1250:20:1, Tau =  20 h)
D = dung    (C:N:P = 100:5:1,   Tau =   5 h)
A = agar    (C:N:P = 100:5:1,   Tau = 0.1 h)
```

Supplementary Figure 4: Ratio of hyphal growth to unicellular growth rate for varying costs of internal transport. The scale of the hyphal advantage over immobile cells depends on resource availability, recalcitrance and internal transport cost β. Pixel colour indicates the ratio between the apparent growth rate of a hyphal colony and a colony of autolytic cells. White pixels indicate that neither kind of organism can grow in the given environment. In panels **(a-d)** we let $\beta = 0.1$, 0.2, 0.3 and 0.5 respectively, in the rows resource can be consumed over 2, 4 and 6 cell radii respectively, and the N:P ratio in each column is 5:1, 20:1 and 40:1 respectively. Also note that there are P limited environments where fungi grow faster than immobile or autolytic cells even in the extreme case where hyphal colonies need 50% more C and N for each unit volume, compared to the unicellular case ($\beta = 0.5$).

needed for motility, relative to the amount of C and N required to synthesise the rest of the cell. The flagella of *Salmonella enterica* is reported to account for 2% of biosynthetic energy expenditure [19], so we have used a value $\alpha = 0.02$. Results are qualitatively similar if we vary the value of α, and there are regions of parameter space where fungi outperform motile cells even when $\alpha = 0$ (see Supplementary Figure 2).

The parameter which has the largest effect on our results is the internal transport cost β (see Supplementary Figures 3 and 4), which represents the cost of internal transport, or the mass of C and N within vesicles, relative to the mass of C and N in both exoenzymes and the core, biosynthetic machinery. In fungi, cell wall materials and exoenzymes are delivered by vesicles [39, 40], and the mass of vesicles relative to other biomass depends on the rate of vesicle delivery, and the fraction of synthesised material that is delivered by vesicle. In yeasts, cell wall accounts for 26-32% of dry weight [41], and for fungi in general, we assume that a similar fraction of material is delivered via vesicles, though the figure may be larger in cases where exoenzymes account for a large fraction of synthesised material. The rate of delivery of vesicles is more difficult to estimate, as the velocity of vesicles varies by several orders of magnitude across the fungal kingdom [32, 34, 35, 42, 43, 44], and there is a non-trivial relationship between velocity measurements, and the mean time that elapses between vesicle formation and vesicle delivery (that is, the time scale corresponding to the rate of vesicle delivery).

We also note that unicellular fungi also contain vesicles, but in our model the cost of those vesicles is accounted for by the core costs for each unit volume, $\mathrm{C_T}+\mathrm{N_I}$. It follows that strictly speaking, the parameter β represents the additional mass of C and N that needs to be transported, due to the fact that if growth and the release of exoenzymes only occur at the hyphal tips, but the necessary materials are taken up and processed elsewhere, there must be material in transit that is not yet helping the organism acquire additional food resource. The distance travelled by materials in hyphal organisms is much greater than the distance travelled by materials in unicellular organisms, which suggests that a larger fraction of materials will be in transit. That is why we assume that internal transport imposes a cost in terms of both C and N. In the absence of data we have chosen to use a value $\beta = 0.1$, but predict that this overestimates the cost of transport in fungi that have highly adapted transport systems. Nevertheless, internal transport would presumably have been far less efficient when hyphal morphology first arose. However, even in the extreme case, where internal transport is so inefficient that hyphal organisms require 50% more C and N for each unit volume than the unicellular case, we still find that hyphal organisms

can grow faster than a colony of immobile or autolytic cells on nutrient poor, P limited substrates (see Supplementary Figure 4).

Supplementary Note 8 Temporal variation in apparent growth rate due to crowding

Our focus is the competition to capture resource, so we model the early stages of colonisation, before crowding effects limit growth. Thus, as a simplifying assumption, we suppose that all organisms capture the same quantity of resource for each unit volume of growth, with daughter cells capturing the same quantity of resource as their parents. The simplifying assumption that crowding is not an issue is generous to unicellular organisms, as unicellular growth and division leads to considerable overlap in the regions that cells can digest. Conversely, one of the key benefits of the polar growth of filamentous organisms is that it enables a more efficient search for space in which to grow [45, 46].

In reality, the specific growth rate of a colony will vary over time [47], and when a colony first starts to grow, none of the cells will have exhausted the local supply of resource. We also note that as a colony grows it is inevitable that at some point, daughter cells will become more crowded than the parent cells, with less resource available for consumption for each unit volume of cell. In this section we consider the time varying case for a colony of immobile cells with a fixed specific growth rate η, to help justify the simplifying assumption of exponential growth, in which a fixed fraction of the colony has exhausted the local supply of resource.

If the total limiting supply is Ω, and individual cells have a growth rate η, the time to exhaust the local supply is $\frac{\Omega}{\eta}$. At time t, any cells that were grown by time $t - \frac{\Omega}{\eta}$ will have exhausted the local resource, and only cells that have not exhausted the local resource can grow. Hence the growth rate at time t is

$$\frac{dV}{dt} = \mu(t)V(t) = \begin{cases} \eta V(t) & \text{if } t \leq \frac{\Omega}{\eta} \\ \eta[V(t) - V(t - \frac{\Omega}{\eta})] & \text{if } t > \frac{\Omega}{\eta} \end{cases} \tag{30}$$

where $V(t)$ is the volume of colonised resource at time t, and $\mu(t)$ is the apparent growth rate at time t.

It follows that

$$\mu(t) = \eta \text{ for } t \leq \frac{\Omega}{\eta} \quad \text{and} \tag{31}$$

$$\lim_{t \to \infty} \mu(t) \quad = \quad \eta(x)\Big[1 + \frac{W_0(- \Omega \exp(-\Omega))}{\Omega}\Big], \tag{32}$$

where W_0 is the principle branch of the Lambert W functional. The value of $\mu(t)$ over time can be found computationally, and three illustrative examples are shown in Supplementary Figure 5(a).

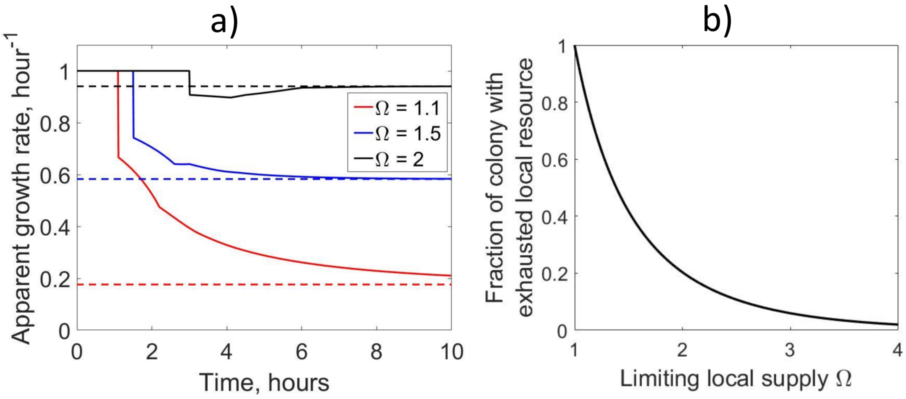

Supplementary Figure 5: Time dependent changes in growth rate for immobile cells in non-crowded conditions. The apparent growth rate of a colony of uncrowded, immobile cells will vary over time, but tends towards a constant. (a) Solid lines indicate the apparent growth rate of the colony over time, when the specific growth rate of each cell is 1 hour^{-1} and the local resource is sufficient to enable $\Omega = 1.1$, 1.5 or 2 cell duplications. Dashed lines indicate the limit case, where the fraction of the colony has exhausted the local resource remains constant over time. **(b)** Fraction of the colony that has exhausted the local resource in the limit case, as a function of the limiting local supply Ω.

Whatever the volume of the colony at time t, the fraction of the colony that has exhausted the local supply of any given resource will be $\frac{V(t-T)}{V(t)}$, where T is the time taken to exhaust the resource in question. When growth is inhibited due to crowding, the apparent growth rate starts to drop, and $V(t)$ is smaller than it would have been in the absence of crowding. Thus the fraction of the colony that has exhausted the local resource $\frac{V(t-T)}{V(t)}$ will be larger than it would have been.

The portion of the colony that has exhausted some but not all essential nutrients is the part of the colony that hyphal architecture is distinctively able to exploit. If an increasing fraction of the colony has exhausted the local supply of some but not all essential nutrients (as will happen when crowding inhibits growth), the growth rate for all categories of organism will be reduced, but the disadvantage is greatest for colonies of unicellular organism.

Fungi can continue to receive non-growth limiting nutrients from the fraction of the colony that has exhausted the local supply of growth limiting nutrient, but if exponential growth is no longer possible, fungi need to adjust the relative proportions of C, N and P digesting exoenzymes they release into the environment, or their internal C:N:P ratio will start to move towards the C:N:P ratio of the substrate.

The best case scenario is that colonies grow exponentially until the volume colonised is limited by the velocity at which the growth front can advance. In that case, the apparent growth rate will tend to become inversely proportional to the time t. For example, a spherical colony whose radius increases with velocity v will have a volume $V(t) = \frac{4\pi}{3}v^3t^3$, a growth rate $\frac{dV}{dt} = 4\pi v^3 t^2$ and an apparent growth rate $\frac{3}{t}$. In such a case, the fraction of the colony that has exhausted the local resource at time t will be $\frac{V(t-T)}{V(t)} = (1 - \frac{T}{t})^3$, where T is the time taken to exhaust the local resource.

As organisms with a hyphal morphology are uniquely capable of benefiting from parts of the colony that have exhausted the local supply of some but not all essential nutrients, we believe that crowding tends to increase the advantage obtained from hyphal morphology. This advantage is further heightened by the polar growth of hyphal organisms, which effectively increases the distance between the hyphal tips and the initial location of the colony [45, 46]. We also note that fungi are uniquely capable of vacuolating mature hyphae whilst translocating the cytoplasm into newly grown hyphae, and here we argue that it is evolutionarily adaptive for fungi to increase the proportion of vacuolated hyphae under crowded conditions. Our analysis indicates that under crowded conditions, fungi will be supplied with an excess of carbon, relative to the uncrowded case. Furthermore, the growth rate of a crowded colony is not limited by the synthetic capacity of the colony as, by definition, the colony could grow faster if there were more space in which to grow. Hence we suggest that it is evolutionarily adaptive for a crowded fungus to use the excess carbon at its disposal to increases the amount of C rich cell wall that it synthesises, at the expense of reducing the amount of N and P rich cytoplasm that it synthesises [48]. This pattern of growth can be viewed as a means of prioritising short term exploratory growth at the expense of increasing the colony's synthetic capacity, and because a crowded colony does not need to increase its synthetic capacity, our analysis suggests that fungi should increase the rate of vacuolation under crowded conditions.

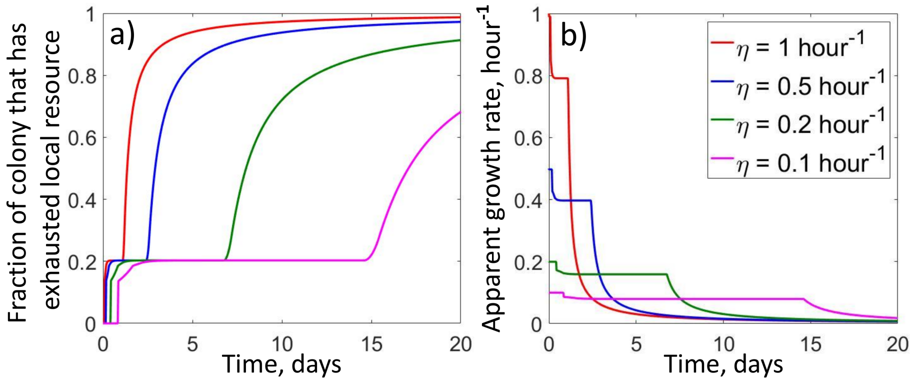

Supplementary Figure 6: Time dependent changes in growth rate for immobile cells if daughter cells become crowded. If the growth rate of a colony of immobile, unicellular organisms is limited by crowding, the fraction of the colony with a locally exhausted supply of resource will increase over time. To produce these figures we assume that a colony of initial size $100\mu\text{m}^3$ grows with a specific growth rate $\eta = 1$, 0.5, 0.2 or 0.1 hour^{-1}, forming a sphere whose radius increases at a maximum rate of 1 mm hour^{-1}. We also assume that the total limiting supply $\Omega = 2$, so after time $T = \frac{2}{\eta}$, the local supply of resource is exhausted. **(a)** Shows the fraction of the colony $\frac{V(t-T)}{V(t)}$ that has exhausted the local supply of nutrient at time t. **(b)** Shows the apparent growth rate of the colony at time t. Note that the flat portions of these graphs are the regions where growth is exponential, and the colony satisfies Supplementary Equations 1-7.

Supplementary Note 9 Constraints on the relative rate of C and N digestion due to substrate accessibility

Organisms can prioritise the digestion, uptake and utilization of particular elements in the substrate by varying the relative proportions of different kinds of digestive enzyme, or by varying the composition or activity of the uptake transport system. However, rates of C and N digestion are not fully independent, as amino acids in proteins contain both C and N, and when an organism grows on recalcitrant substrates such as wood, extracellular polymers such as lignin and cellulose may need to be broken down before the local proteins can be accessed. These considerations motivate further refinement of our model by including an additional accessibility parameter δ, to reflect the fact that some fraction of the available C must be digested in order to access N, irrespective of the category of organism. In other words, we can impose the additional constraint that for all organisms,

$$\frac{x_{\mathrm{C}}}{\mathrm{C}_{\mathrm{E}}} \geq \frac{x_{\mathrm{N}}}{\mathrm{N}_{\mathrm{E}}}\delta, \tag{33}$$

where $x_{\rm C}$ and $x_{\rm N}$ denote the relative densities of C and N digesting exoenzymes respectively, and δ denotes the minimum fraction of local C that must be degraded in order to digest the local supply of N.

For example, if the C:N ratio of the substrate is 200:1, imposing a value of $\delta = 0.1$ forces organisms to digest at least 20 C for every N they acquire. If organisms only require a C:N ratio of 10:1, imposing this additional constraint would necessitate a two-fold increase in the number of C digesting exoenzymes for unicellular organisms. In the case of fungi, imposing the constraint described by Supplementary Equation 33 inhibits the growth rate at lower C:N ratios than is the case for unicellular organisms. For example, if the colony as a whole requires a C:N ratio of 10:1, and half the colony has exhausted the local supply of N but can still acquire C, it would be optimal for the growing margin to acquire resource at a C:N ratio of 5:1. In that case, insisting that in all parts of the colony at least 20 C are digested for every N acquired would necessitate an four-fold increase in the number of C digesting exoenzymes, compared to the two-fold increase for unicellular organisms.

Thus we find that imposing a constraint on the ratio between the rate of C and N digestion results in fungi generating a greater proportion of excess carbon, compared to other organisms (see Supplementary Figure 7). We also note that in our model, when fungi are obliged to digest more C than they need in order to obtain N, they do not obtain any benefit from the parts of the colony that have exhausted the local supply of nutrients other than C, precisely because the fungus is already supplied with more C than it can use. In reality, it is unlikely that the excess carbon will simply be wasted. It is more likely that fungi will find a way to use that carbon in an adaptive manner, such as using it to increase the rate of vesicle delivery, increase the rate of protein turnover, or increase the thickness of the cell wall. In other words, it is reasonable to assume that all else being equal, an organism that burns more carbon obtains a higher fitness than an organism that burns less carbon. However, in our model, where CUE and demand for C, N and P are fixed, we find that imposing the additional constraint described by Supplementary Equation 33 reduces the growth rate of fungi more than other organisms (see Supplementary Figure 8).

To revise the model with the additional constraint described by Supplementary Equation 33, we note that if $\rm C_T \leq \frac{\delta N_I C_E}{N_E}$, cells need to digest more C than they require in order to access N. We let

$$\hat{\rm C} = \max\left[\frac{\delta {\rm N_I C_E}}{\rm N_E}, {\rm C_T}\right], \tag{34}$$

and note that $\hat{\rm C}(1+x)$ replaces ${\rm C_T}(1+x)$ as the mass of C that must be digested for each unit volume of immobile cell. Consequently, the limiting local supply for immobile or

autolytic cells becomes

$$\Omega_\iota(x) = \Omega_a(x) = \min\left[\frac{\kappa^2 \mathrm{C_E}}{\hat{\mathrm{C}}(1+x)}, \frac{\kappa^2 \mathrm{N_E}}{\mathrm{N_I}(1+x)}, \frac{\kappa^2 \mathrm{P_E}}{\mathrm{P_I}}\right]. \tag{35}$$

Similarly, for motile cells the limiting local supply becomes

$$\Omega_m(x) = \min\left[\frac{\kappa^2 \mathrm{C_E} - \hat{\mathrm{C}}x}{\hat{\mathrm{C}}(1+\alpha+x)}, \frac{\kappa^2 \mathrm{N_E} - \mathrm{N_I}x}{\mathrm{N_I}(1+\alpha+x)}, \frac{\kappa^2 \mathrm{P_E}}{\mathrm{P_I}}\right]. \tag{36}$$

These changes have a minor effect, as in general, local supply of C will not be the limiting resource. The principle effect of imposing the constraint described by Supplementary Equation 33 is a potentially dramatic reduction in the rate of resource use Γ for any given relative density of exoenzymes x, as now, many of the exoenzymes generate C that is not actually used. For example, to generate the same useful supply of resource, an immobile cell requires a density of exoenzymes proportional to $(\hat{\mathrm{C}} + \mathrm{N_I})(1 + x) + \mathrm{P_I}$ instead of $(\mathrm{C_T} + \mathrm{N_I})(1 + x) + \mathrm{P_I}$, and $\hat{\mathrm{C}}$ may be significantly larger than $\mathrm{C_T}$.

In the case of immobile cells, Supplementary Equation 1 is superseded by the equation

$$\Gamma_\iota(x) = \min\left[\frac{[(\mathrm{C_T} + \mathrm{N_I})(1+x) + \mathrm{P_I}]x(\mathrm{C_T} + \mathrm{N_I})}{[(\hat{\mathrm{C}} + \mathrm{N_I})(1+x) + \mathrm{P_I}]\tau}, \quad \lambda\right] \tag{37}$$

$$= \min\left[\frac{\theta_\iota(\mathrm{C_T} + \mathrm{N_I})x}{\tau}, \quad \lambda\right]. \tag{38}$$

Note that

$$\theta_\iota = \frac{(\mathrm{C_T} + \mathrm{N_I})(1+x) + \mathrm{P_I}}{(\hat{\mathrm{C}} + \mathrm{N_I})(1+x) + \mathrm{P_I}} \tag{39}$$

can be interpreted as the fraction of mass digested that is actually needed for growth, and imposing the additional constraint described by Supplementary Equation 33 effectively rescales τ to $\frac{\tau}{\theta_\iota}$. However, unlike the parameter τ, the fraction θ_ι depends on the relative demand for C, N and P, and this varies with the relative density of exoenzymes x. Similarly, in the case of autolytic cells we have

$$\Gamma_a(x) = \min\left[\frac{[(\mathrm{C_T} + \mathrm{N_I})(1+x) + \mathrm{P_I}](\mathrm{C_T} + \mathrm{N_I})x}{[(\hat{\mathrm{C}} + \mathrm{N_I})(1+x) + \mathrm{P_I}]\tau} + \frac{\epsilon\mu(\mathrm{C_T} + \mathrm{N_I} + \mathrm{P_I})}{e^{\mu T} - 1}, \lambda\right] \tag{40}$$

$$= \min\left[\frac{\theta_a(\mathrm{C_T} + \mathrm{N_I})x}{\tau} + \frac{\epsilon\mu(\mathrm{C_T} + \mathrm{N_I} + \mathrm{P_I})}{e^{\mu T} - 1}, \lambda\right]. \tag{41}$$

For motile cells we have

$$\Gamma_m(x) = \min\left[\frac{[(\mathrm{C_T}+\mathrm{N_I})(1+\alpha+x)+\mathrm{P_I}](\mathrm{C_T}+\mathrm{N_I})x}{[(\hat{\mathrm{C}}+\mathrm{N_I})(1+\alpha+x)+\mathrm{P_I}]\tau}, \quad \lambda\right] \tag{42}$$

$$= \min\left[\frac{\theta_m(\mathrm{C_T}+\mathrm{N_I})x}{\tau}, \lambda\right]. \tag{43}$$

Given these new functions for Γ and Ω, the specific growth rates η, time to exhaust the local supply T, and apparent growth rate μ are calculated as before. However, in the case of motile cells, the time taken to synthesise the exoenzymes that are needed to replace the exoenzymes that will be left behind is now $\frac{\tau}{\theta_a}$, instead of τ, so the apparent growth rate of motile cells μ_m satisfies the equation

$$\mu_m = \eta_m(x)\frac{\exp\left[\mu_m\frac{\Omega(x)}{\eta(x)}\right]-1}{\exp\left[\mu_m\frac{\Omega(x)}{\eta(x)}\right]-\exp[-\mu_m\frac{\tau}{\theta}]}. \tag{44}$$

To apply the additional constraint to the case of fungal colonies, we simply note that the relative density of C digesting exoenzymes is no longer determined by Supplementary Equation 29 alone, as we also impose the constraint

$$x_{\mathrm{C}} \geq \frac{\delta x_{\mathrm{N}}\mathrm{C_E}}{\mathrm{N_E}}. \tag{45}$$

Supplementary Note 10 Varying demand for C, N and P

Cellular responses to changes in nutrient availability are highly complex [49, 9, 50], but for the sake of simplicity we assume that all organisms in all environments have the same metabolic core, with a fixed demand for C, N and P for each unit volume of growth. In reality, there will be a trade-off between the costs and benefits of maintaining a different concentration of C, N or P within an organism [4], and the optimal concentration of each kind of resource will vary between organisms and environments. Elements that are available in excess of metabolic requirements may either be mineralised or stored [4, 51, 52], and respiration of excess C (overflow metabolism) is a well-documented microbial phenomenon [9, 49]. We also note that the C:N:P ratio and dry mass density of fungi can be altered by changing the fraction of fungal volume that is occupied by vacuoles. Compared to metabolically active hyphae, highly vacuolated hyphae contain far less N and P rich cytoplasm, but require the same quantity of carbon rich cell wall [48]. Organisms are

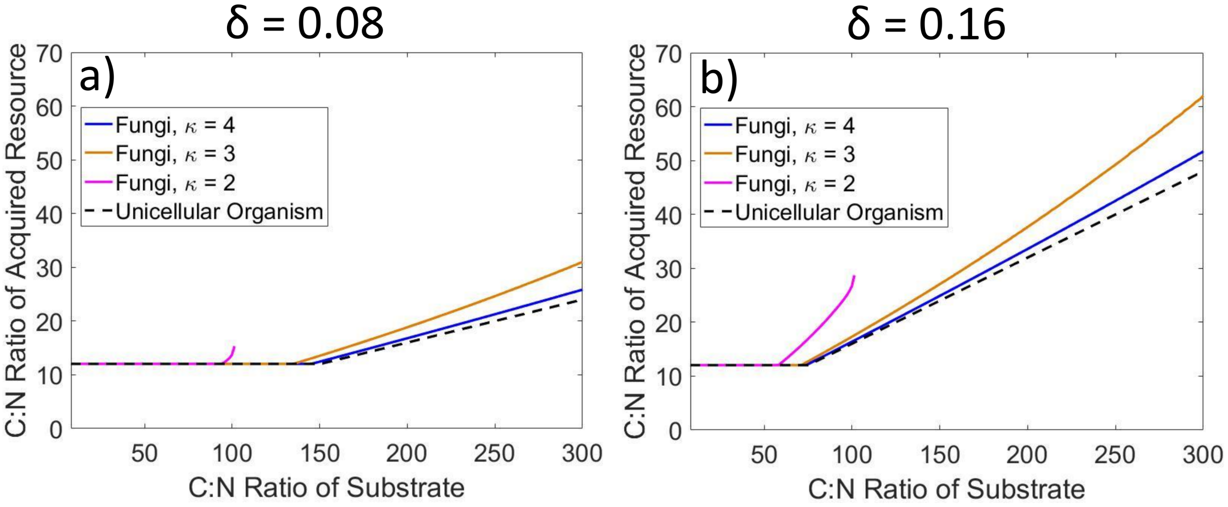

Supplementary Figure 7: The impact of substrate accessibility on the relative amount of C:N released. If the rate of C digestion is determined by the need to access N, the C:N ratio of fungi will be higher than the C:N ratio of unicellular organisms. In panel **(a)** we assume that $\delta = 0.08$, so at least 8% of the local carbon must be digested in order to access N, while in panel **(b)** we let $\delta = 0.16$. In both plots we assume that organisms require a C:N ratio of 12:1, and the N:P ratio and density of the substrate are 40:1 and 0.5 g ml^{-1} respectively. Note that the C:N ratio of resource acquired by unicellular organisms (dotted black line) is imposed by Supplementary Equation 33, and because fungi continue to obtain C after the local N has been exhausted, they acquire resource at a higher C:N ratio than unicellular organisms. If a significant fraction of the colonised resource is N but not C depleted, as will be the case when the C:N ratio is high and κ is small, the difference between the C:N ratio acquired by fungi and the C:N ratio acquired by unicellular organisms can be considerable. Changing the recalcitrance parameter τ does not alter the scale of this effect.

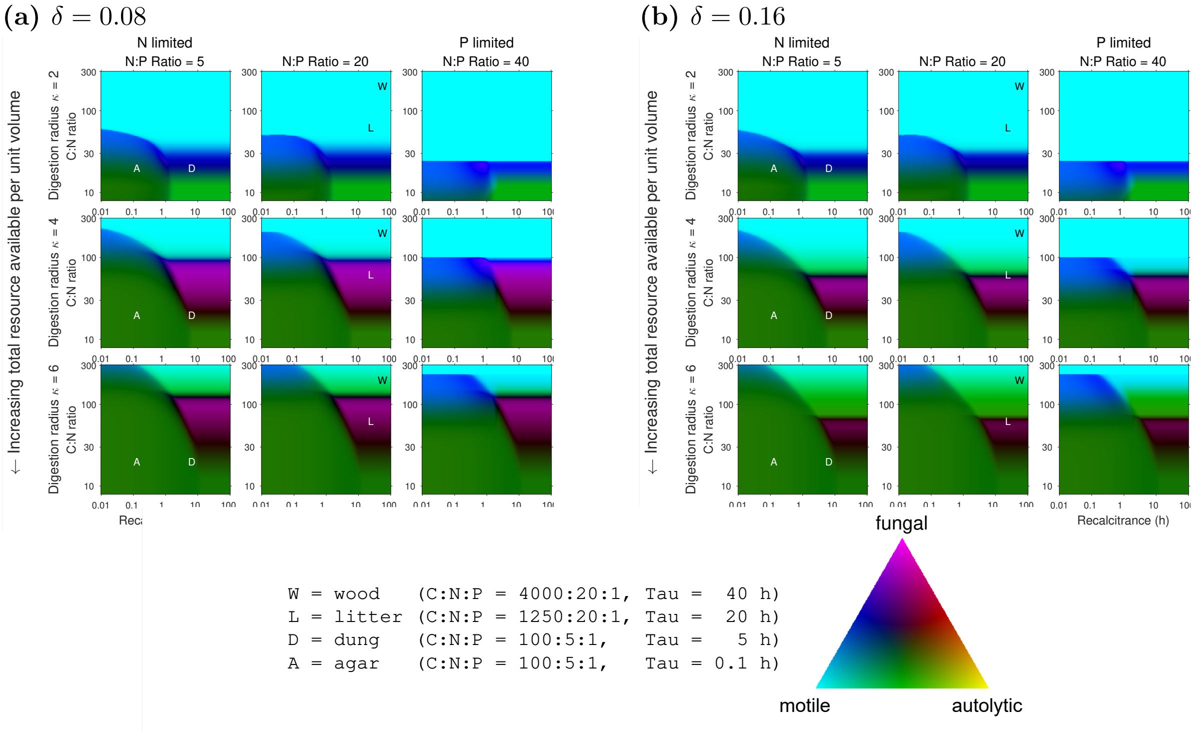

Supplementary Figure 8: The impact of substrate accessibility on the competitiveness of different classes of organism. Relative growth rates of fungi and cells depends on resource availability, the recalcitrance of the substrate, and the quantity of C that must be digested in order to access N. Each panel indicates relative performance as the C:N ratio and recalcitrance are varied over a wide but physiologically relevant range, shown on log scales. In panel**(a)** we assume that $\delta = 0.08$, so at least 8% of the local carbon must be digested in order to access N, while in panel **(b)** $\delta = 0.16$. Rows correspond to increasing amount of resource availability as κ is varied over 2, 4 and 6 cell radii (see Main Text Fig. 3). Columns correspond to varying N:P ratios set to 5:1, 20:1, and 40:1. The substrate has a dry mass of 0.5 g ml^{-1} in all cases.

therefore capable of changing the amount of C, N and P that is used for each unit volume of growth. Nevertheless, the C:N:P ratios of micro-organisms are relatively constrained compared to the C:N:P ratios of the substrates they consume [3, 4, 6]. For example, when *Escherichia coli* is grown in a chemostat, the C:P ratio of the cells varies by a factor of two when the C:P ratio of the growth medium is varied by a factor of 100 [53]. This suggests that broadly speaking, the elemental composition of organisms is homeostatically regulated, but there are large differences in the C:N:P ratios and dry mass densities of different species, reflecting differences in the environmental stoichiometry of the niches that they occupy [2, 3, 4, 6].

If organisms can support a higher concentration of nucleotides, amino acids or proteins, or if they can respire more rapidly, they should be able to synthesise materials more rapidly (all else being equal). For example, bacteria which are typically found in environments that enable rapid growth tend to contain higher concentrations of RNA, and have lower C:P ratios [54]. On the other hand, synthesising a daughter cell that also contains a high concentration of synthesised components takes longer than synthesising a daughter cell with a low concentration of synthesised components (all else being equal). Furthermore, if the materials needed for growth come from a limited local supply, fewer daughter cells can be synthesised if those daughter cells contain a relatively high internal concentration of resource. Thus a model prediction of how optimal dry mass densities and optimal C:N:P ratios vary across environments and categories of organism requires a relatively detailed analysis of the costs and benefits of maintaining higher or lower concentrations of various cellular components. Nevertheless, our simple model indicates that the cost of obtaining a higher concentration of non-growth limiting resource is higher for unicellular organisms than for hyphal organisms, because exoenzymes that digest non-growth limiting resource have a longer useful lifespan in the case of hyphal organisms. Thus we expect that the C:N:P ratios of fungi will tend to be closer to the C:N:P ratios of their substrates than is the case for unicellular organisms. This is in broad agreement with the available data, which shows that fungi typically have significantly higher C:N and C:P ratios than bacteria [3, 6]. Our analysis suggests that this is because fungi continue to acquire the C produced by C digesting exoenzymes after the local supply of N or P has been exhausted.

Supplementary References

[1] Keiblinger, K. M. *et al.* The effect of resource quantity and resource stoichiometry on microbial carbon-use-efficiency. *FEMS Microbiol. Ecol.* **73**, 430–440 (2010).

[2] Soares, M. & Rousk, J. Microbial growth and carbon use efficiency in soil: Links to fungal-bacterial dominance, SOC-quality and stoichiometry. *Soil Biol. Biochem.* **131**, 195–205 (2019).

[3] Cleveland, C. C. & Liptzin, D. C: N: P stoichiometry in soil: is there a "Redfield ratio" for the microbial biomass? *Biogeochemistry* **85**, 235–252 (2007).

[4] Elser, J. J. *et al.* Nutritional constraints in terrestrial and freshwater food webs. *Nature* **408**, 578 (2000).

[5] Zhang, J. & Elser, J. J. Carbon: nitrogen: phosphorus stoichiometry in fungi: a meta-analysis. *Front. Microbiol.* **8**, 1281 (2017).

[6] Mouginot, C. *et al.* Elemental stoichiometry of fungi and bacteria strains from grassland leaf litter. *Soil Biol. .Biochem.* **76**, 278–285 (2014).

[7] Bakken, L. R. & Olsen, R. A. Buoyant densities and dry-matter contents of microorganisms: conversion of a measured biovolume into biomass. *Appl. Environ. Microbiol.* **45**, 1188–1195 (1983).

[8] Zimmerman, S. B. & Trach, S. O. Estimation of macromolecule concentrations and excluded volume effects for the cytoplasm of *Escherichia coli*. *J. Mol. Biol.* **222**, 599–620 (1991).

[9] Schimel, J. P. & Weintraub, M. N. The implications of exoenzyme activity on microbial carbon and nitrogen limitation in soil: a theoretical model. *Soil Biol. Biochem.* **35**, 549–563 (2003).

[10] Gibson, B., Wilson, D. J., Feil, E. & Eyre-Walker, A. The distribution of bacterial doubling times in the wild. *Proc. Roy. Soc. B* **285**, 20180789 (2018).

[11] Zakhartsev, M. & Reuss, M. Cell size and morphological properties of yeast *Saccharomyces cerevisiae* in relation to growth temperature. *FEMS Yeast Res.* **18**, foy052 (2018).

[12] Rousk, J. & Bååth, E. Growth of saprotrophic fungi and bacteria in soil. *FEMS Microbiol. Ecol.* **78**, 17–30 (2011).

[13] Corless, R. M., Gonnet, G. H., Hare, D. E., Jeffrey, D. J. & Knuth, D. E. On the Lambert W function. *Adv. Comp. Math.* **5**, 329–359 (1996).

[14] Durand, P. M., Sym, S. & Michod, R. E. Programmed cell death and complexity in microbial systems. *Curr. Biol.* **26**, R587–R593 (2016).

[15] Allocati, N., Masulli, M., Di Ilio, C. & De Laurenzi, V. Die for the community: an overview of programmed cell death in bacteria. *Cell Death & Disease* **6**, e1609 (2015).

[16] Benomar, S. *et al.* Nutritional stress induces exchange of cell material and energetic coupling between bacterial species. *Nat. Comms.* **6**, 6283 (2015).

[17] Vostinar, A. E., Goldsby, H. J. & Ofria, C. Suicidal selection: Programmed cell death can evolve in unicellular organisms due solely to kin selection. *Ecol. Evol.* **9**, 9129–9136 (2019).

[18] Nedelcu, A. M., Driscoll, W. W., Durand, P. M., Herron, M. D. & Rashidi, A. On the paradigm of altruistic suicide in the unicellular world. *Evolution* **65**, 3–20 (2011).

[19] Macnab, R. *Escherichia coli* and *Salmonella*: Cellular and molecular biology. In: Flagella and motility, 123–145 (ASM Press, Washington, D.C., 1996).

[20] Archer, D. B. & Peberdy, J. F. The molecular biology of secreted enzyme production by fungi. *Crit. Rev. Biotech.* **17**, 273–306 (1997).

[21] Wösten, H. A., Moukha, S. M., Sietsma, J. H. & Wessels, J. G. Localization of growth and secretion of proteins in *Aspergillus niger*. *Microbiology* **137**, 2017–2023 (1991).

[22] Cai, Y. J., Chapman, S. J., Buswell, J. A. & Chang, S.-T. Production and distribution of endoglucanase, cellobiohydrolase, and β-glucosidase components of the cellulolytic system of *Volvariella volvacea*, the edible straw mushroom. *Appl. Environ. Microbiol.* **65**, 553–559 (1999).

[23] Masai, K. *et al.* Square-plate culture method allows detection of differential gene expression and screening of novel, region-specific genes in *Aspergillus oryzae*. *App. Microbiol. Biotech.* **71**, 881–891 (2006).

[24] Jedd, G. Fungal evo-devo: organelles and multicellular complexity. *TICB* **21**, 12–9 (2011).

[25] Trinci, A. P. J. Influence of width of peripheral growth zone on radial growth rate of fungal colonies on solid media. *J. Gen. Microbiol.* **67**, 325–344 (1971).

[26] Markham, P. Occlusions of septal pores in filamentous fungi. *Mycol. Res.* **98**, 1089–1106 (1994).

[27] Tegelaar, M. & Wösten, H. A. Functional distinction of hyphal compartments. *Scientific Reports* **7**, 1–6 (2017).

[28] Bleichrodt, R.-J., Hulsman, M., Wösten, H. A. B. & Reinders, M. J. T. Switching from a unicellular to multicellular organization in an *Aspergillus niger* hypha. *mBio* **6**, e00111–15 (2015).

[29] Van Peer, A. F., Müller, W. H., Boekhout, T., Lugones, L. G. & Wösten, H. A. Cytoplasmic continuity revisited: closure of septa of the filamentous fungus *Schizophyllum commune* in response to environmental conditions. *PLoS One* **4**, e5977 (2009).

[30] Tlalka, M., Watkinson, S. C., Darrah, P. R. & Fricker, M. D. Continuous imaging of amino-acid translocation in intact mycelia of *Phanerochaete velutina* reveals rapid, pulsatile fluxes. *New Phytol.* **153**, 173–184 (2002).

[31] Tlalka, M., Fricker, M. D. & Watkinson, S. C. Imaging of long-distance α-aminoisobutyric acid translocation dynamics during resource capture by *Serpula lacrymans. Appl. Environ. Microbiol.* **74**, 2700–2708 (2008).

[32] Olsson, S. & Gray, S. N. Patterns and dynamics of ^{32}P-phosphate and labelled 2-aminoisobutyric acid (^{14}C-AIB) translocation in intact basidiomycete mycelia. *FEMS Microbiol. Ecol.* **26**, 109–120 (1998).

[33] Lindahl, B., Finlay, R. & Olsson, S. Simultaneous, bidirectional translocation of ^{32}P and ^{33}P between wood blocks connected by mycelial cords of *Hypholoma fasciculare. New Phytol.* **150**, 189–194 (2001).

[34] Jennings, D. H. Translocation of solutes in fungi. *Biol. Rev. Cam. Phil. Soc.* **62**, 215–243 (1987).

[35] Cairney, J. W. G. Translocation of solutes in ectomycorrhizal and saprotrophic rhizomorphs. *Mycol. Res.* **96**, 135–141 (1992).

[36] Fricker, M. D., Heaton, L. L., Jones, N. S. & Boddy, L. The mycelium as a network. *Microbiol. Spectrum* **5** (2017).

[37] Dechesne, A., Wang, G., Gülez, G., Or, D. & Smets, B. F. Hydration-controlled bacterial motility and dispersal on surfaces. *Proc. Natl. Acad. Sci. USA* **107**, 14369–14372 (2010).

[38] Pion, M. *et al.* Gains of bacterial flagellar motility in a fungal world. *Appl. Environ. Microbiol.* **79**, 6862–6867 (2013).

[39] Riquelme, M. *et al.* Fungal morphogenesis, from the polarized growth of hyphae to complex reproduction and infection structures. *Microbiol. Mol. Biol. Rev.* **82**, e00068–17 (2018).

[40] Money, N. P. Insights on the mechanics of hyphal growth. *Fung. Biol. Rev.* **22**, 71–76 (2008).

[41] Nguyen, T., Fleet, G. & Rogers, P. Composition of the cell walls of several yeast species. *App. Microbiol. Biotech.* **50**, 206–212 (1998).

[42] Lew, R. R. How does a hypha grow? the biophysics of pressurized growth in fungi. *Nature Rev Microbiol* **9**, 509–518 (2011).

[43] Heaton, L. L. M., López, E., Maini, P. K., Fricker, M. D. & Jones, N. S. Growth-induced mass flows in fungal networks. *Proc. R. Soc. B* **277**, 3265–3274 (2010).

[44] Heaton, L. L., Lopez, E., Maini, P. K., Fricker, M. D. & Jones, N. S. Advection, diffusion, and delivery over a network. *Phys. Rev. E* **86**, 021905 (2012).

[45] Held, M., Edwards, C. & Nicolau, D. V. Probing the growth dynamics of neurospora crassa with microfluidic structures. *Fung. Biol.* **115**, 493–505 (2011).

[46] Molin, P., Gervais, P., Lemiere, J. & Davet, T. Direction of hyphal growth: a relevant parameter in the development of filamentous fungi. *Res. Microbiol.* **143**, 777–784 (1992).

[47] Prosser, J. I. Growth-kinetics of mycelial colonies and aggregates of ascomycetes. *Mycol. Res.* **97**, 513–528 (1993).

[48] Paustian, K. & Schnurer, J. Fungal growth-response to carbon and nitrogen limitation - a theoretical-model. *Soil Biol. Biochem.* **19**, 613–620 (1987).

[49] Russell, J. B. & Cook, G. M. Energetics of bacterial growth: balance of anabolic and catabolic reactions. *Microbiol. Mol. Biol. Rev.* **59**, 48–62 (1995).

[50] Gutteridge, A. *et al.* Nutrient control of eukaryote cell growth: a systems biology study in yeast. *BMC biology* **8**, 68 (2010).

[51] Manzoni, S., Jackson, R. B., Trofymow, J. A. & Porporato, A. The global stoichiometry of litter nitrogen mineralization. *Science* **321**, 684–686 (2008).

[52] Manzoni, S. Flexible carbon-use efficiency across litter types and during decomposition partly compensates nutrient imbalances—results from analytical stoichiometric models. *Front. Microbiol.* **8**, 661 (2017).

[53] Makino, W., Cotner, J. B., Sterner, R. W. & Elser, J. Are bacteria more like plants or animals? growth rate and resource dependence of bacterial C: N: P stoichiometry. *Func. Ecol.* **17**, 121–130 (2003).

[54] Elser, J. *et al.* Biological stoichiometry from genes to ecosystems. *Ecol. lett.* **3**, 540–550 (2000).
