# Supplementary material for: A mechanistic explanation of the transition to simple multicellularity in fungi
Source: Nat Commun. 2020 May 22;11:2594. doi: 10.1038/s41467-020-16072-4 (PMC7244713; doi:10.1038/s41467-020-16072-4)
Supplement: Supplementary file 4 — Description of Additional Supplementary Files [file 41467_2020_16072_MOESM4_ESM.pdf]

Description of Additional Supplementary Files

File name: Supplementary Software 1

Description: The Supplementary Software ZIP file includes three alternative routes to run the Fungal Transition to Multicellularity (T2M) simulation, along with a user manual (Fungal_T2M_Manual.pdf) that describes Installation and Operation of the software. A GUI version is provided as either a standalone executable file for Windows 10 (Fungal_T2M_v1.exe), or a self-installing App for MATLAB (The MathWorks, Natick, MA) 2019b or later (Fungal_T2M_v1.mlappinstall), that is platform independent. In addition, a set of MATLAB command-line scripts are provided that can be run in MATLAB, that are also platform independent.
