# Supplementary material for: A mechanistic explanation of the transition to simple multicellularity in fungi
Source: Nat Commun. 2020 May 22;11:2594. doi: 10.1038/s41467-020-16072-4 (PMC7244713; doi:10.1038/s41467-020-16072-4)
Supplement: Supplementary file 5 — Supplementary Software 1 [file 41467_2020_16072_MOESM5_ESM.zip › Fungal_T2M_Manual.pdf]

LUKE HEATON - NICK JONES - MARK FRICKER

# FUNGAL T2M - SOFTWARE MANUAL

PLANT SCIENCES, OXFORD
FIRST EDITION

Copyright © 2019 Luke Heaton - Nick Jones - Mark Fricker

All rights reserved.

Redistribution of this manual and the associated software and use in source and binary forms, with or without modification, are permitted provided that the following conditions are met:

- Redistributions of source code must retain the above copyright notice, this list of conditions and the following disclaimer.
- Redistributions in binary form must reproduce the above copyright notice, this list of conditions and the following disclaimer in the documentation and/or other materials provided with the distribution.
- Neither the name of Plant Sciences, University of Oxford nor the names of its contributors may be used to endorse or promote products derived from this software without specific prior written permission.

This software is provided by the copyright holders and contributors "as is" and any express or implied warranties, including, but not limited to, the implied warranties of merchantability and fitness for a particular purpose are disclaimed. In no event shall Mark Fricker be liable for any direct, indirect, incidental, special, exemplary, or consequential damages (including, but not limited to, procurement of substitute goods or services; loss of use, data, or profits; or business interruption) however caused and on any theory of liability, whether in contract, strict liability, or tort (including negligence or otherwise) arising in any way out of the use of this software, even if advised of the possibility of such damage.

PUBLISHED BY PLANT SCIENCES, OXFORD

FIRST EDITION

*First printing, November 2019*

*Current version, March 2020*

# *Contents*


**1 Installation** **5**
1.1 Overview . . . . . . . . . . . . . . . . . . . . . . . . . . . . . . . . . . . . . . . . . . 5
1.2 Download all files from the github repository . . . . . . . . . . . . . . . . . . . . . . 6
1.3 Installation of the MATLAB app . . . . . . . . . . . . . . . . . . . . . . . . . . . . 6
1.4 Installation of the stand-alone program . . . . . . . . . . . . . . . . . . . . . . . . . 6
1.5 Installation of additional program files needed . . . . . . . . . . . . . . . . . . . . . 8

**2 Running the simulation using the GUI** **9**
2.1 Introduction . . . . . . . . . . . . . . . . . . . . . . . . . . . . . . . . . . . . . . . . 9
2.2 Tuneable environmental parameters . . . . . . . . . . . . . . . . . . . . . . . . . . . 11
2.3 Tuneable organism parameters . . . . . . . . . . . . . . . . . . . . . . . . . . . . . . 11
2.4 Running the simulation . . . . . . . . . . . . . . . . . . . . . . . . . . . . . . . . . . 11
2.5 Displaying the colour-coded results . . . . . . . . . . . . . . . . . . . . . . . . . . . 12
2.6 Performance of different classes of organism on specific resources . . . . . . . . . . 15
2.7 Inclusion of user defined resources . . . . . . . . . . . . . . . . . . . . . . . . . . . . 16

**3 Command Line Version** **17**
3.1 Overview . . . . . . . . . . . . . . . . . . . . . . . . . . . . . . . . . . . . . . . . . . 17
3.2 Running the simulation for varying resource quality and recalcitrance . . . . . . . . 18
3.3 Running the simulation for a specific resource quality in varying abundance . . . . . 20

*Acknowledgements*

This work was supported by a Visiting Fellowship at The Institute of Advanced Studies in Durham, The Leverhulme Trust (RPG-2015-437) and The Human Frontier Science Program (RGP0053/2012).

The export_fig package was written by Oliver Woodford and Yair Altman and downloaded from:
https://uk.mathworks.com/matlabcentral/fileexchange/23629-export-fig

The screen capture package was written by Yair Altman (2020) and downloaded from:.
https://www.mathworks.com/matlabcentral/fileexchange/24323-screencapture-get-a-screen-capture-of-a-figure-frame-or-component

The colorcet.m package was written by Peter Kovesi and downloaded from:
https://peterkovesi.com/projects/colourmaps/

Peter Kovesi. Good Colour Maps: How to Design Them. arXiv:1509.03700 [cs.GR] 2015
https://arxiv.org/abs/1509.03700

# 1
# Installation

## 1.1 Overview

The code to run the simulation is provided as open source under a GNU General Public License v3.0 from:

https://github.com/markfricker/Fungal_multicellularity

The code is provided in three alternative formats:

- A set of script files and functions that will run in MATLAB® on any platform;
- A MATLAB® app with a GUI interface that can be installed in MATLAB® on any platform (requires MATLAB® release 2019b or later, the Image Processing Toolbox™ and a screen resolution of 1600 x 900 or greater);
- A compiled standalone version that can be installed and run on Windows 10, 64 bit platforms and requires a screen resolution of 1600 x 900 or greater;

On the home page of the github repository, click on the green **Clone or download** button (Figure 1.1) and then the blue **Download ZIP** button.

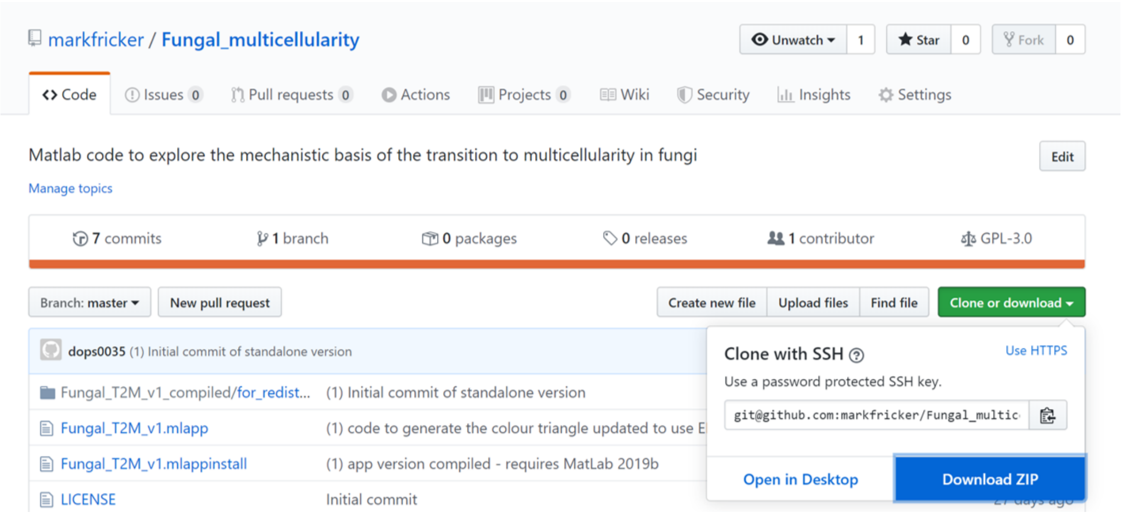


*Figure 1.1: Download the software in a zip file from the github repository*

## 1.2 *Download all files from the github repository*

Once the file has downloaded, extract all the files. If you intend to run the script versions, the extracted folder needs to be on the MATLAB® path.

## 1.3 *Installation of the MATLAB app*

The MATLAB® app installer file, *Fungal_T2M_v1.mlappinstall*, contains everything necessary to install and run the App within the MATLAB® environment, including the source code, supporting data, information (such as product dependencies), and the app icon (Figure 1.2). The installation time is less than a minute.

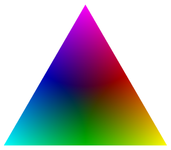

*Figure 1.2: The program icon*

Double-clicking on the *Fungal_T2M_v1.mlappinstall* file should launch MATLAB® if it is not already running, and install the app in the app toolbar (Figure 1.3). The program can be run, by clicking on the icon in the toolbar.

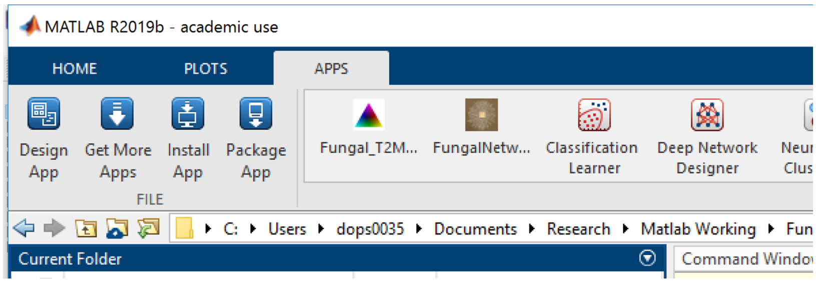


*Figure 1.3: Location of the Fungal_T2M app in the APPS menu within MATLAB®*

The app version of the program also requires the MATLAB® Image Processing Toolbox™ to be installed.

## 1.4 *Installation of the stand-alone program*

The software has been tested on Windows 10, and requires a minimum screen resolution of 1600 x 900. In addition, an appropriate version of the MATLAB® Compiler Runtime (MCR) is required to install the set of shared libraries that enable execution of the compiled MATLAB® application. The MCR should automatically download from the MathWorks website when the program is installed for the first time. Alternatively MCR can be downloaded from:

`http://www.mathworks.com/products/compiler/mcr`.

To install the MCR and standalone package, double-click the compiled MATLAB self-extracting *Fungal_T2M_v1.exe* file located in the subfolder:

`\Fungal_T2M_v1_compiled\for_redistribution`

This extracts the MATLAB® Runtime Installer from the archive, along with all the files that make up the deployed MATLAB® en-

vironment. Once all the files have been extracted, the MATLAB® Runtime Installer starts automatically. When the MATLAB® Runtime Installer starts, it displays the following dialog box:

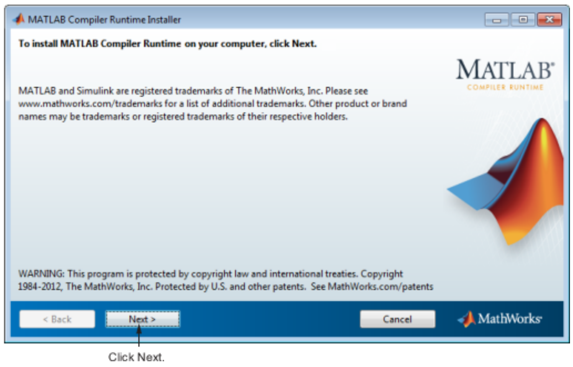


Read the information and then click **Next** to proceed with the installation.

Specify the folder in which you want to install the MATLAB® runtime in the Folder Selection dialog box and click **Next**.[1] It is recommended to keep the default settings as this ensures the path to other program files is set automatically.

[1] On Windows systems, you can have multiple versions of the MATLAB® runtime on your computer, but only one installation for any particular version. If you already have an existing installation, the MATLAB® runtime Installer does not display the Folder Selection dialog box because you can only overwrite the existing installation in the same folder.

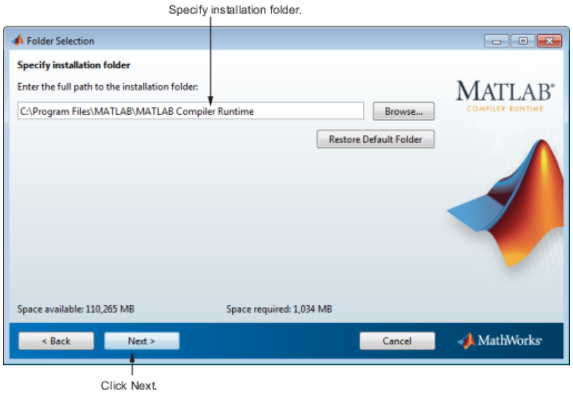


Confirm your choices and click **Install**. The MATLAB® Runtime Installer starts copying files into the installation folder. Installation takes about 10 minutes.

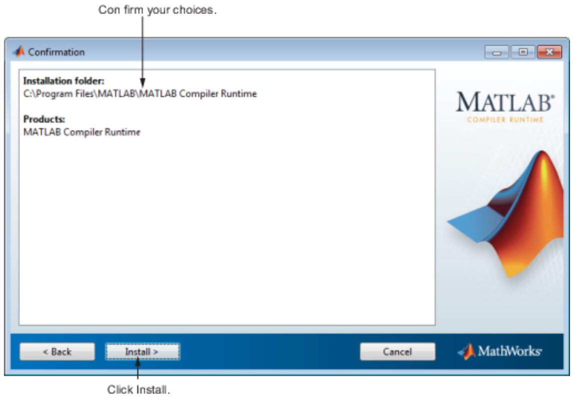


Click **Finish** to exit the installer.

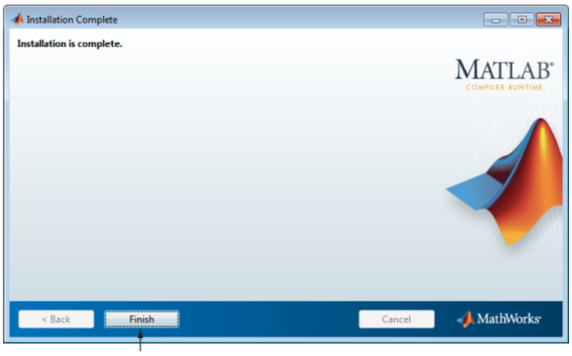


**MATLAB® Runtime Installer Readme File:** A readme.txt file is included with the MATLAB Runtime Installer. This file, visible when the MATLAB Runtime Installer is expanded, provides more detailed information about the installer and the switches that can be used with it.

## 1.5 Installation of additional program files needed

A number of additional files needed to provide high quality output need to be installed at the same time as the main program. The latest version of Java needs to be installed, and is available from:

`http://www.java.com/en/`

Output of images at full resolution uses *export_fig.m* originally written by Oliver Woodford (2008-2014) and now maintained by Yair Altman (2015-). This is included in the installation. However, when exporting to vector format (PDF or EPS) this function requires that ghostscript is installed on your system. Ghostscript can be downloaded from:

`http://www.ghostscript.com`.

When exporting images to eps and pdf formats, *export_fig* additionally requires pdftops, from the Xpdf suite of functions. This is included in the xpdf tools package and can be downloaded from:

`https://www.xpdfreader.com/download.html`

# 2

# Running the simulation using the GUI

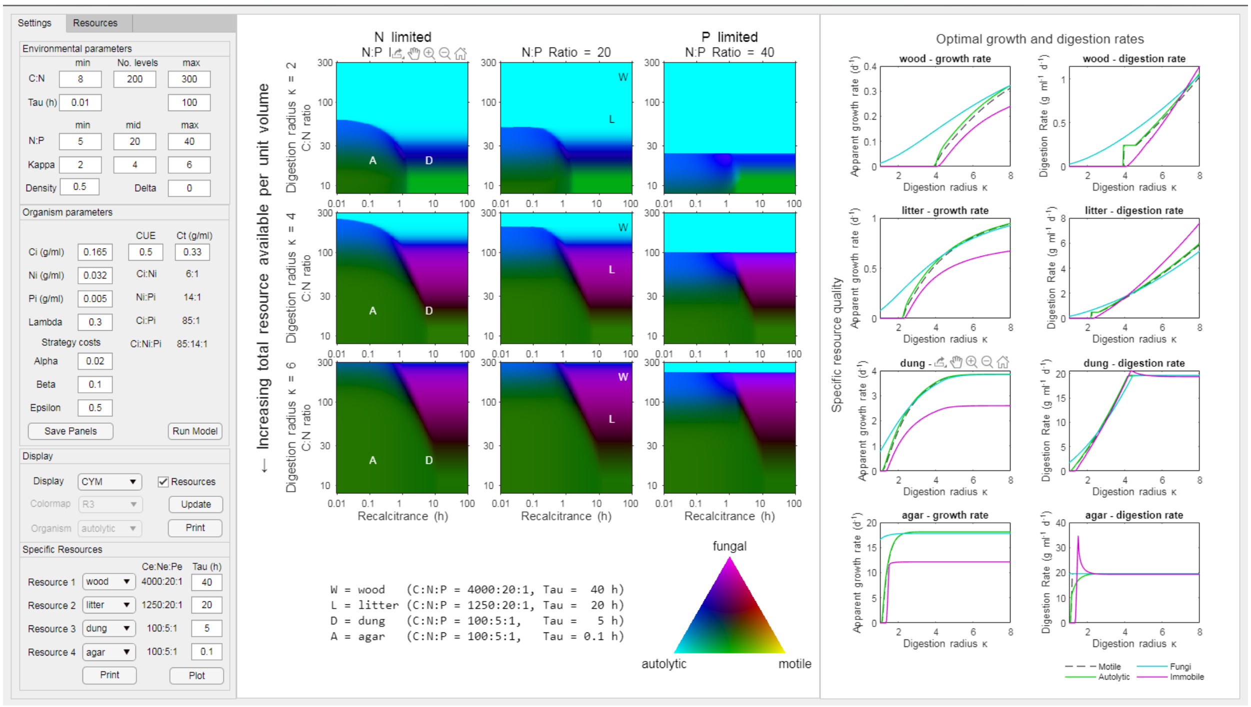


*Figure 2.1: The GUI interface for the fungal multicellularity simulation*

## 2.1 Introduction

The MATLAB app version and the standalone version both provide the same interface to run the simulation (Figure 2.1). The set of left-hand panels allow the user to change the parameters controlling the external resource environment and the internal organism parameters, including the cost terms for each of the different strategies. The definition of the parameters is given in Table 2.1.

*Table 2.1: Definition and values of modelling parameters*

| **Tunable Environmental Parameters** | **Symbol and Units** | **Interpretation** |
| --- | --- | --- |
| Supply of C, N and P | $C_E, N_E, P_E$ | Grams of C, N and P per millilitre of substrate, set by the C:N and N:P ratio of the resource, and the resource density in g $ml^{-1}$ |
| Recalcitrance | $\tau$ hours | Time required for an exoenzyme to supply a mass of C, N or P equal to the total mass required to synthesise the exoenzyme in question |
| Relative digestion radius | $\kappa$ | Cylindrical cells and hyphae digest resource over a distance times the cell radius |
| Resource accessibility | $\delta$ | Ratio of C that has to be digested to release each N, to reflect that N is embedded within C-rich polymers |

| **Tuneable Organism Parameters** | **Symbol and Units** | **Interpretation** |
| --- | --- | --- |
| Core demand for C, N and P | $C_I = 0.165$ g $ml^{-1}$<br>$N_I = 0.032$ g $ml^{-1}$<br>$P_I = 0.005$ g $ml^{-1}$ | Core demand for C, N and P per unit volume of any organism. |
| Carbon Use Efficiency | CUE = 0.5 | Ratio of $C_I$ for growth to total C required ($C_T$), including respiration |
| Motility cost | $\alpha = 0.02$ | Mass of C and N required to synthesise motile apparatus, relative to core demand for C and N |
| Vesicle transport cost | $\beta = 0.1$ | Mass of C and N in vesicles, relative to the total C and N in exoenzymes and the fungal core |
| Recycling efficiency | $\epsilon = 0.5$ | Fraction of C, N and P that is recouped by autolysis |
| Maximal rate of resource use | $\lambda = 0.3$ g $ml^{-1}$ $hour^{-1}$ | Maximal mass of resource any cell can use per unit time and volume |

| **Model Variables** | **Symbol and Units** | **Interpretation** |
| --- | --- | --- |
| Relative density of exoenzymes | $x$ | Mass of C and N used for exoenzymes for unicellular organisms relative to the core demand for C and N in unicells |
| Relative density of C, N or P exoenzymes | $x_C, x_N, x_P$ | Mass of C and N used for C, N or P digesting exoenzymes for fungal organisms relative to the core demand for C and N |

| **Functions of $x$** | **Symbol and Units** | **Interpretation** |
| --- | --- | --- |
| Time to exhaust local resource | $T$ hours | Time until one of C, N or P is locally exhausted |
| Time to exhaust C, N or P | $T_C, T_N, T_P$ hours | Time until relevant element is locally exhausted |
| Specific growth rate of growing cell | $\eta$ $h^{-1}$ | Rate of synthesis of cell biomass, per unit of cell biomass, in the growing cells |
| Apparent growth rate | $\mu$ $h^{-1}$ | Volumetric rate of colonisation, per unit volume colonised |
| Total limiting supply | $\Omega$ | Maximum number of daughter cells and their exoenzymes that can be synthesised, given the local supply of resource |
| Rate of resource use | $\Gamma$ g $ml^{-1}$ $h^{-1}$ | Total mass of resource used per unit time and volume, in the growing cells |

## 2.2 *Tuneable environmental parameters*

The C:N ratio of the resource ranges from the minimum value (*min*), typically around 5, to the maximum value (*max*), typically around 300, with the number of intermediate levels set by *levels* (Figure 2.2). The C:N ratio forms the y-axis for each of the resultant plots (on a $log_{10}$ scale). The x-axis is set by the recalcitrance ($\tau$), and ranges from *min*, typically around 0.01, to *max*, typically around 100, with the same number of intermediate levels. Results are plotted for three values of the N:P ratio, set by *min*, *mid* and *max* (default 5, 20 and 40, respectively) and three values for the overall resource availability, given by the radius of digestion ($\kappa$), again set by *min*, *mid* and *max* (default 2, 4 and 6, respectively).

The overall resource density in g ml$^{-1}$ is used to convert the resource C:N and C:P ratios into grams ml$^{-1}$ of $C_E$, $N_E$, and $P_E$.

The value of the additional accessibility parameter $\delta$, reflects the fact that some fraction of the available C must be digested in order to access N, irrespective of the category of organism. For example, if the C:N ratio of the substrate is 200:1, imposing a value of $\delta = 0.1$ forces organisms to digest at least 20 C for every N they acquire.

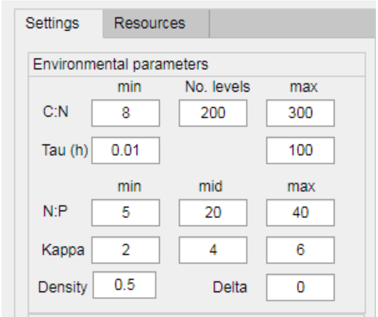


*Figure 2.2: Controls for the environmental parameters that define the resource quantity and quality*

## 2.3 *Tuneable organism parameters*

The internal C, N and P required by all organisms are set by $C_i$, $N_i$ and $P_i$, respectively, in g ml$^{-1}$. These values are also displayed as the molar C:N, N:P, C:P and C:N:P ratio in the adjacent text boxes (Figure 2.3). The default values are typical of fungal and microbial cells, although there is considerable variation in both the absolute amounts and the relative ratios.

The total amount of C required ($C_t$) also includes that used in respiration. This is set by the carbon use efficiency (*CUE*), defined as the fraction of growth to total assimilation. The default value of CUE is set as 50%, but values can be much lower than this[1].

The maximum rate that resource that can be used (by any organism) is set by *Lambda*, with a default value of 0.3 g ml$^{-1}$ h$^{-1}$. This would equate to a doubling time of just under 1h for the default resource density, and represents an upper limit that is unlikely to be achieved for organisms in the wild.

In addition, autolytic, motile and fungal cells have a single organism-specific cost parameter that helps to define their different strategies. Thus, *Alpha* is the additional cost of being motile (default 0.02), *Beta* is the cost associated with internal transport (default 0.1), whilst *Epsilon* is the fraction of an autolytic cell that can be recovered by recycling (default 0.5).

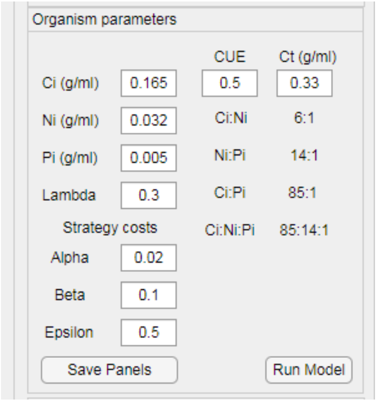


*Figure 2.3: Parameter settings for the biological organisms*

[1] Margarida Soares and Johannes Rousk. Microbial growth and carbon use efficiency in soil: Links to fungal-bacterial dominance, SOC-quality and stoichiometry. *Soil Biol. Biochem.*, 131: 195–205, 2019

## 2.4 *Running the simulation*

The **Run Model** button will iterate through the external resource environmental parameters for each organism, and returns pseudo-

colour coded maps showing which class of organism is predicted to grow most rapidly under each set of conditions, according to the inset colour triangle (Figure 2.4). The default settings are matched to the data reported in the main text. The run time is about 160-180 min on a Windows 10 machine with an Intel® Core™ i7-8750H CPU running at 2.20GHz with 32GB RAM. Reduced run times can be achieved by decreasing the number of *levels* (the minimum suggested is around 20) as run time increases with *levels*$^2$.

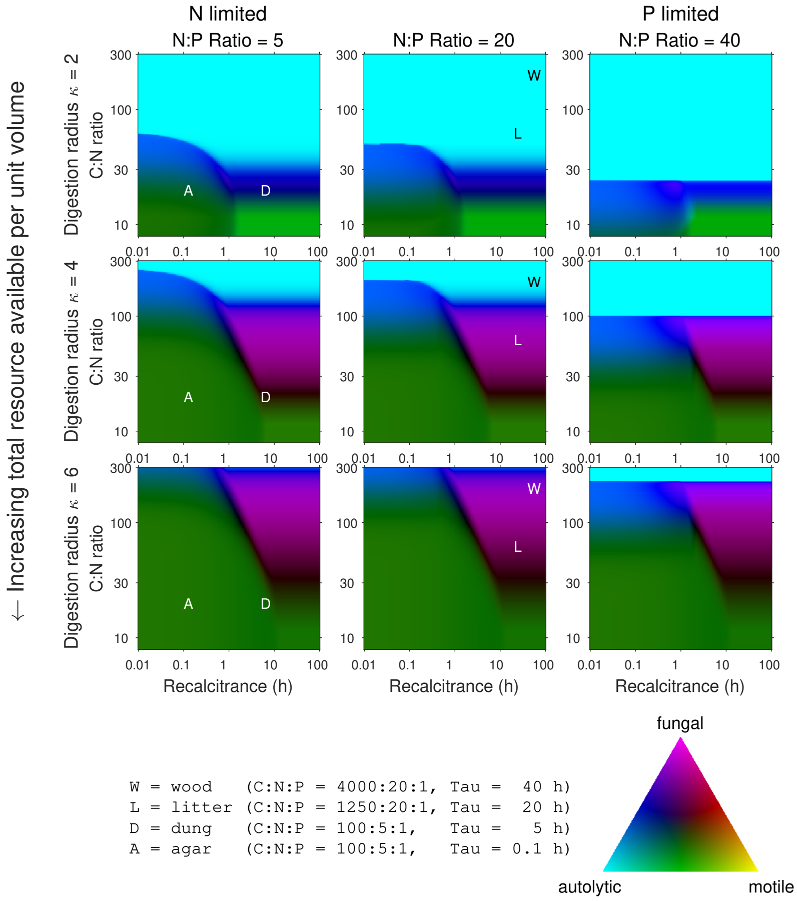


*Figure 2.4: Colour-coded maps showing the relative performance of each type of organism across a wide range of resource quantity and quality.*

Regions of parameter space where motile cells dominate are coloured cyan, regions where fungi dominate are magenta, and regions where autolytic cells dominate are yellow. In many regions, two or more organisms may fare equally well. Thus, there are typically extensive regions where motile cells and fungal organisms both thrive (blue), or motile and autolytic unicells are equivalent (green).

## 2.5 *Displaying the colour-coded results*

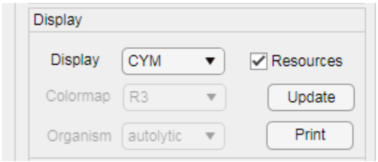


*Figure 2.5: Display options*

The cyan-magenta-yellow maps show the relative performance of each class of organism compared to the others. In addition, in the *Display* panel (Figure 2.5), the *Display* drop down menu can be used

to show how the individual growth rates compare to the fastest growing cell with no resource restriction, by selecting the *Growth* option (Figure 2.6). This provides comparative plots for each N:P Ratio for each digestion radius, whilst the *Organism* for comparison has to be selected from the corresponding drop down menu. The default lookup table (LUT) gives a heatmap scale. Alternative LUTs can be selected from the *Colormap* dropdown menu. the *Resources* checkbox superimposes letter codes on the plots that correspond to the set of four specific resources described in Section 2.6 below, and shown in the figure legend.

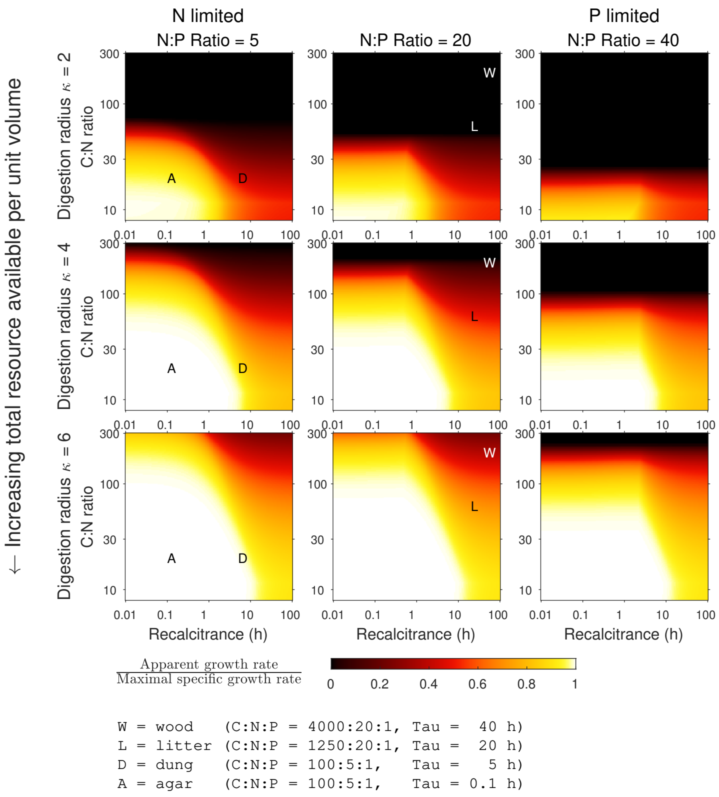


*Figure 2.6: Colour-coded maps showing the relative growth of each type of organism compared to the maximum growth of any type of organism. The letter codes superimposed correspond to the region of parameter space associated with specific resources set out in the figure legend.*

Alternatively, the relative growth rate of a fungal organism can be compared specifically with one of the other classes of organism to visualise directly the magnitude of the hyphal advantage (Figure 2.7). The default colormap is on a perceptually uniform rainbow scale[2], but other options can be selected from the *Colormap* drop-down menu, including a number of diverging LUTs (prefix 'D') that highlight the relative performance quite well. The **Print** button saves a copy of the display as a png or pdf file.

[2] P. Kovesi. Good colour maps: How to design them. *arXiv:1509.03700 [cs.GR]*, 2015

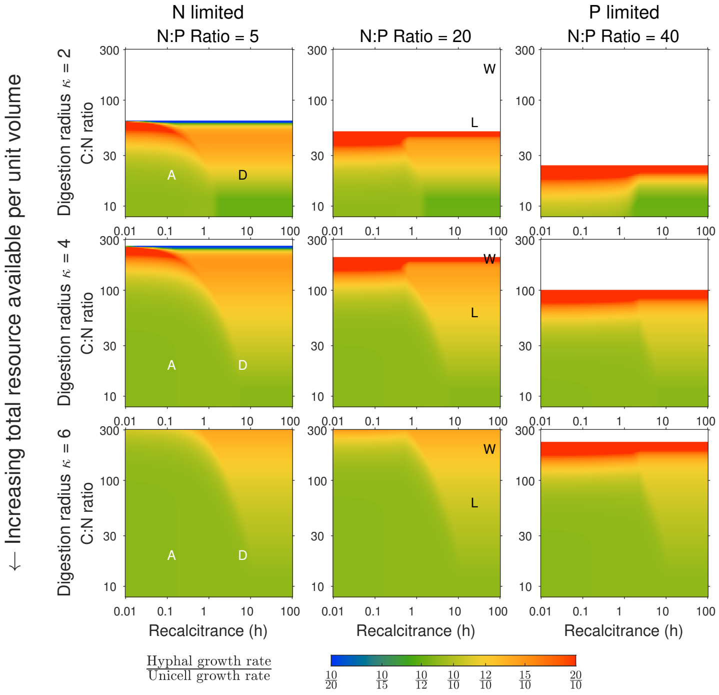


*Figure 2.7: Colour-coded maps showing the relative growth of a hyphal organism compared to a specific class of unicellular organism.*

```
W = wood   (C:N:P = 4000:20:1, Tau =  40 h)
L = litter (C:N:P = 1250:20:1, Tau =  20 h)
D = dung   (C:N:P = 100:5:1,   Tau =   5 h)
A = agar   (C:N:P = 100:5:1,   Tau = 0.1 h)
```

## 2.6 *Performance of different classes of organism on specific resources*

The colour-coded maps display results for a very extensive range of resource environments. The *Specific resources* panel (Figure 2.8) provides options to plot the performance for four specific resources, with the defaults set to wood (C:N:P 4000:20:1, $\tau$ = 40h), leaf litter (C:N:P 1250:20:1, $\tau$ = 20), dung (C:N:P 100:5:1, $\tau$ = 5h) and agar (C:N:P 100:5:1, $\tau$ = 0.1h).

The **Run** button runs the simulation for these specific resource configurations using the other parameters defined for the full model (Figure 2.9)

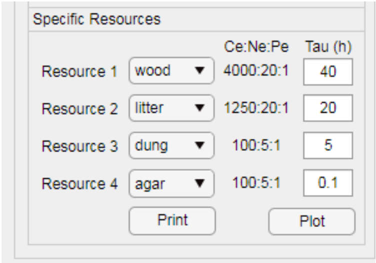


*Figure 2.8: Selection of specific resources*

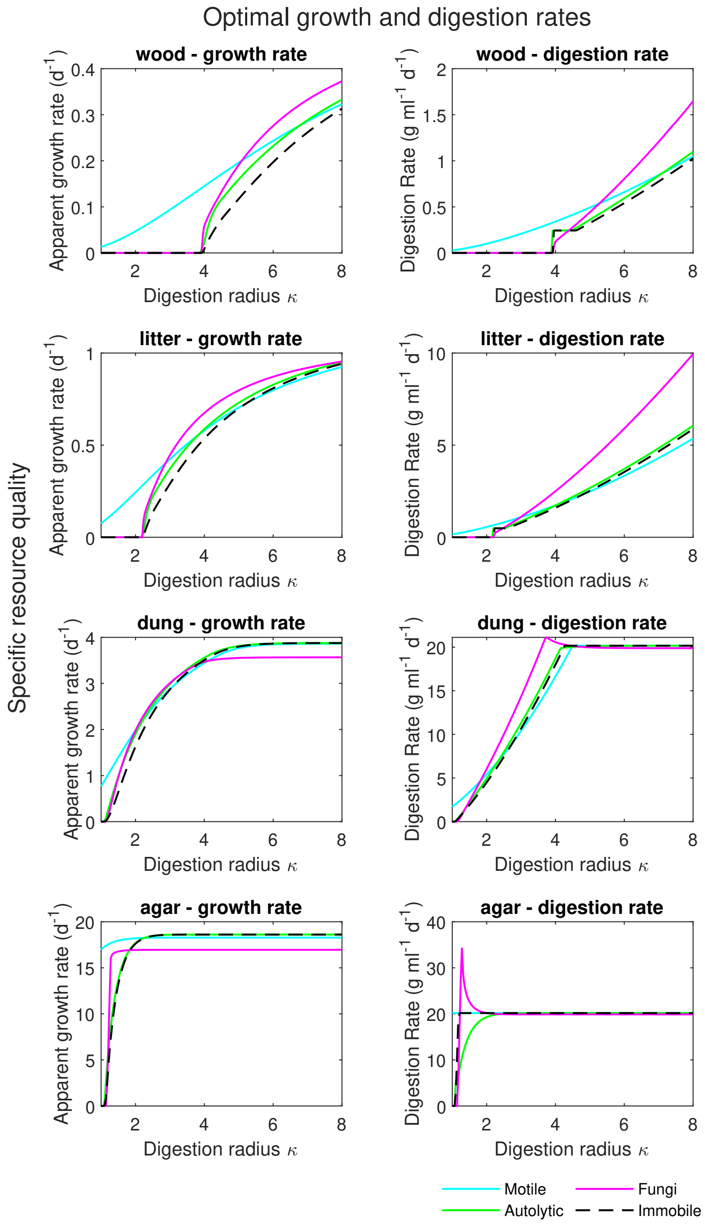


*Figure 2.9: Colour-coded maps showing the relative growth of a hyphal organism to a specific class of unicellular organism.*

Whilst the values for C:N:P are widely reported in the literature, the values for $\tau$ are not defined experimentally for most substrates.

Thus, there is the option to vary $\tau$ for each resource using the adjacent edit box. For direct comparison, the same resource can be selected in all four dropdown menus and the simulation run with different values of $\tau$. Increasing the value of tau significantly decreases the growth rate for all organisms, but in general, changing tau only has a small effect on the relative performance of different classes of organisms.

## 2.7 *Inclusion of user defined resources*

Given that there are many different resource possibilities other than the four defaults used, there is an option to define more substrates using the **Resources** tab at the top of the control panels (Figure 2.10)

The Table includes the four default resource types. Additional resources can be included using the **Add** button, which adds an additional row to the table. Likewise the **Remove** button will delete the currently selected entry. The modified table can be saved using the **Save** button, and re-loaded using the **Load** button. The **Update** button adds the new set of resources to the **Resource** drop down menus in the **Specific Resource** panel in the main **Settings** tab. The effect of changing resources can then be explored using the **Run** button.

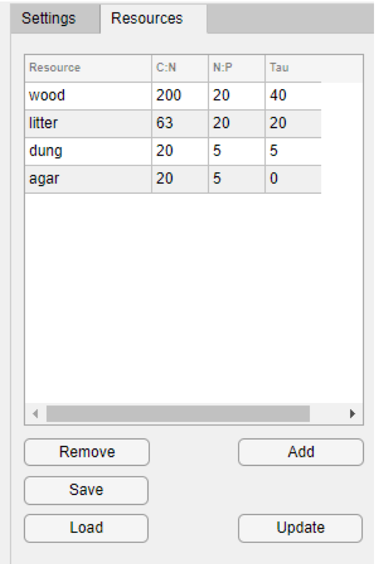


*Figure 2.10: Resource table to allow addition of specific resources with user-defined C:N:P ratios and recalcitrance (Tau)*

# 3
# Command Line Version

## 3.1 Overview

The set of MATLAB® scripts that can be used to run the different components of the simulation are given in Table 3.1. These have been tested on versions 2017a and should work in any subsequent release of MATLAB® on any platform. They are the same functions as called by the GUI, or embedded in the standalone versions of the program.

| **Script Name** | **Description** |
|---|---|
| *vary_CNP_and_tau.m* | The main script to run the simulation and generate colour-coded maps of relative performance |
| *vary_kappa.m* | Script to generate the growth rate and rate of digestion for each class of organism in a specific resource environment |

| **Function Name** | **Description** |
|---|---|
| *find_best_cell.m* | Function to determine numerically the best growth rate ($\mu$) for a given investment in digestive enzymes ($x$) using the parameter values for a cell growing at the maximum possible rate without substrate depletion which implements equations 1-2 of the Supplementary Information. |
| *find_best_immobile.m* | Function to determine numerically the best growth rate ($\mu$) for a given investment in digestive enzymes ($x$) using the parameter values for immobile cells. Calls the function *find_mu_given_x_immobile.m*. Implements equations 1-6 of the Supplementary Information. |
| *find_best_autolytic.m* | Function to determine numerically the best growth rate ($\mu$) for a given investment in digestive enzymes ($x$) using the parameter values for autolytic cells. Calls the function *find_mu_given_x_autolytic.m*. Implements equations 7-10 of the Supplementary Information. |

| | |
|---|---|
| *find_best_motile.m* | Function to determine numerically the best growth rate ($\mu$) for a given investment in digestive enzymes ($x$) using the parameter values for motile cells. Calls the function *find_mu_given_x_motile.m*. Implements equations 11-13 of the Supplementary Information. |
| *find_best_fungi.m* | Function to determine numerically the best growth rate ($\mu$) for a given investment in digestive enzymes ($x$) using the parameter values for fungal cells. Calls the function *find_mu_given_U_fungi.m*. Implements equations 14-17 of the Supplementary Information. |

| **Display functions** | **Description** |
|---|---|
| *generate_CYM_merge.m* | Function to generate the Cyan-Yellow-Magenta maps of relative growth rate. If the fastest growing colony has grown by a factor of n, and a slower growing colony has grown by a factor of m, the channels for the colonies are coloured accordingly with intensity 1 and m/n respectively. Higher n increases the apparent difference in colour, making a lighter CYM merge. |
| *heatmap_comparison.m* | Generates colour-coded ratio heatmap of the relative growth of fungal cells and autolytic cells in the standard set of resource environments. Calls *colorcet.m* to define a perceptually uniform ratio colourmap ('R3') and *applycolourmap.m* to apply it to the ratio growth image. See SI Figure 3. |
| *heatmap_growth_inhibition.m* | Generates a heatmap showing the relative growth of each class of organism compared to the maximum growth possible with unlimited resource. Calls *colorcet.m* to define a cold-hot colourmap ('L3') and *applycolourmap.m* to apply it to the relative growth inhibition image. See SI Figure 4. |

Table 3.1: MATLAB scripts and functions

## 3.2 *Running the simulation for varying resource quality and recalcitrance*

Open the *vary_CNP_and_tau.m* script in the MATLAB® Editor. The first part of the code sets the resource and organism parameters (Listing 3.1), which can be modified manually to explore different environmental resources or impose different costs on the strategy used by each organism. For initial trials, the *res1* parameter can be set to a much lower value (20) to reduce the computation time, whist still titrating the range of C:N and recalcitrance values (Figure 3.2).

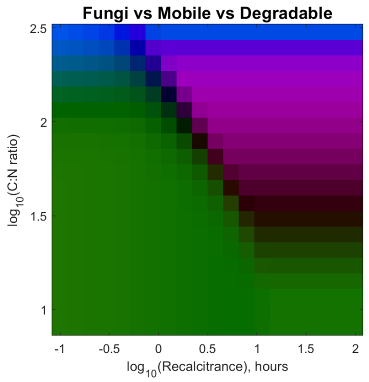


*Figure 3.1: Coarse output for rapid prototyping*

Listing 3.1: Setting parameter values at the start of the vary_CNP_and_tau code

```
%% set up parameters. These can be manually edited.
% res1 is the number of different values for the C:N ratio and recalcitrance
% that are tried
res1 = 200;

% xres determines the number of different values for x that are tried in finding
% the optimal solution. Final value found is accurate to xres^2.
xres = 200;

% sres is the number of iterations used in finding best solution for a given
% environment and a given rate of sythesis.
sres = 60;

% N_to_P set the N:P ratio of the resource.
N_to_P = 20;

% kappa is the radius of digestion, normalised to the radius of the cells/hypha,
% and sets the total amount of resource available.
kappa = 6;

% C_to_N_min and C_to_N_max define the limits of the C:N ratio in the resource.
% The number of intermediate steps is controlled by res1. The C:N ratio is
% plotted on the y-axis of the resultant map.
C_to_N_min = 8;
C_to_N_max = 300;

% tau_min and tau_max define the limits of the resource recalcitrance, where tau
% is the time in hours for hydrolases to digest their own mass. The number of
% intermediate steps is controlled by res1. The recalcitrance is plotted on the
% x-axis of the resultant map.
tau_min = 0.1;
tau_max = 100;

% Ci is the mass of carbon needed per unit volume of growth, in g per ml
Ci = 0.165;
Ni = 0.032;
Pi = 0.005;

% the carbon use efficiency is the fraction of C for growth versus total C
% for respiration and growth
CUE = 0.5;

% Ct is the total C required, including respiratory C set by the carbon use
% efficiency
Ct = Ci/CUE;

% dry weight in grams per ml of the substrate.
density = 0.5;

% epsilon is the efficiency of recycling for senscent autolytic cells.
epsilon = 0.5;

% alpha is the additional mass of machinery needed for cell mobility, relative
% to the mass of essential metabolic machinery.
alpha = 0.02;

% beta is the mass of material in vesicles, relative to the mass of the rest of
% the fungus, including hydrolases.
beta = 0.1;

% lambda is the maximum rate of resource use per unit volume, in g per ml per
% hour.
lambda = 0.3;

% Δ is the ratio of C that has to be digested to release each N, to reflect
% that N is embedded within C-rich polymers.
Δ = 0;
```

The output is a *res*1*xres*1 array of the relative growth (Mu) and the amount of digestive enzymes released (x) for each class of organism for the selected ranges of C:N and tau. The relative growth can be compared using the three different display scripts:

- *generate_CYM_merge.m* gives a three-way comparison using a cyan-yellow-magenta colour-code for motile, autolytic and fungal cells, respectively.
- *heatmap_growth_inhibition.m* compares the relative growth of each organism against a cell with no limitation on resource availability such that the colony continues to grow at the maximum rate under the conditions selected.
- *heatmap_comparison.m* compares the rate of fungal growth explicitly with autolytic cells to provide a more quantitative visual indication of the hyphal advantage in different resource environments.

## 3.3 *Running the simulation for a specific resource quality in varying abundance*

The second main script (Listing 3.2) requires the user to define the resource quality (C:N:P ratios and recalcitrance), but then calculates the relative growth rate for each class of organism and rate of digestion to give the optimal strategy. As with the *vary_CNP_and_tau* script, the first section has to be edited manually to set up different parameter configurations. The output is automatically generated as a plot of growth rate against resource abundance (varying kappa) and rate of digestion for the four classes of organism.

Listing 3.2: Setting parameter values at the start of the vary_kappa code

```
%% set up parameters. These can be manually edited.
% res1 is the number of different values for kappa that we try
res1 = 200;

% xres determines the number of different values for x that are tried in finding
% the optimal solution. Final value found is accurate to xres^2.
xres = 200;

% sres is the number of iterations used in finding best solution for
% a given environment and a given rate of sythesis
sres = 60;

% k_min and k_max are the minimum and maximum values for the resource
% acquisition radius that we try
k_min = 1;
k_max = 8;

% Ci is the mass of carbon needed per unit volume of growth, in g per ml
Ci = 0.165;
Ni = 0.032;
Pi = 0.005;

% the carbon use efficiency is the fraction of C for growth versus total C
% for respiration and growth
```

```
CUE = 0.5;

% Ct is the total C required, including respiratory C set by the carbon use
% efficiency
Ct = Ci/CUE;

% C_to_P and N_to_P set the C:P and N:P ratio of the resource, respectively.
C_to_P = 2000;
N_to_P = 10;

% tau is the time in hours for hydrolases to digest their own mass and reflects
% the recalcitrance of the resource.
tau = 40;

% dry weight in grams per ml of the substrate
density = 0.5;

% Δ is the minimal fraction of C that must be digested in order to
% digest the available N
Δ = 0;

% epsilon is the efficiency of recycling for autolytic cells
epsilon = 0.5;

% alpha is the mass of machinery needed for cell mobility, relative
% to the mass of essential machinery
alpha = 0.02;

% beta is the mass of material in vesicles, relative to the mass of
% the rest of the fungus, including hydrolases
beta = 0.1;

% lambda is the maximum rate of resource use per unit volume,
% in g per ml per hour
lambda = 0.3;
```

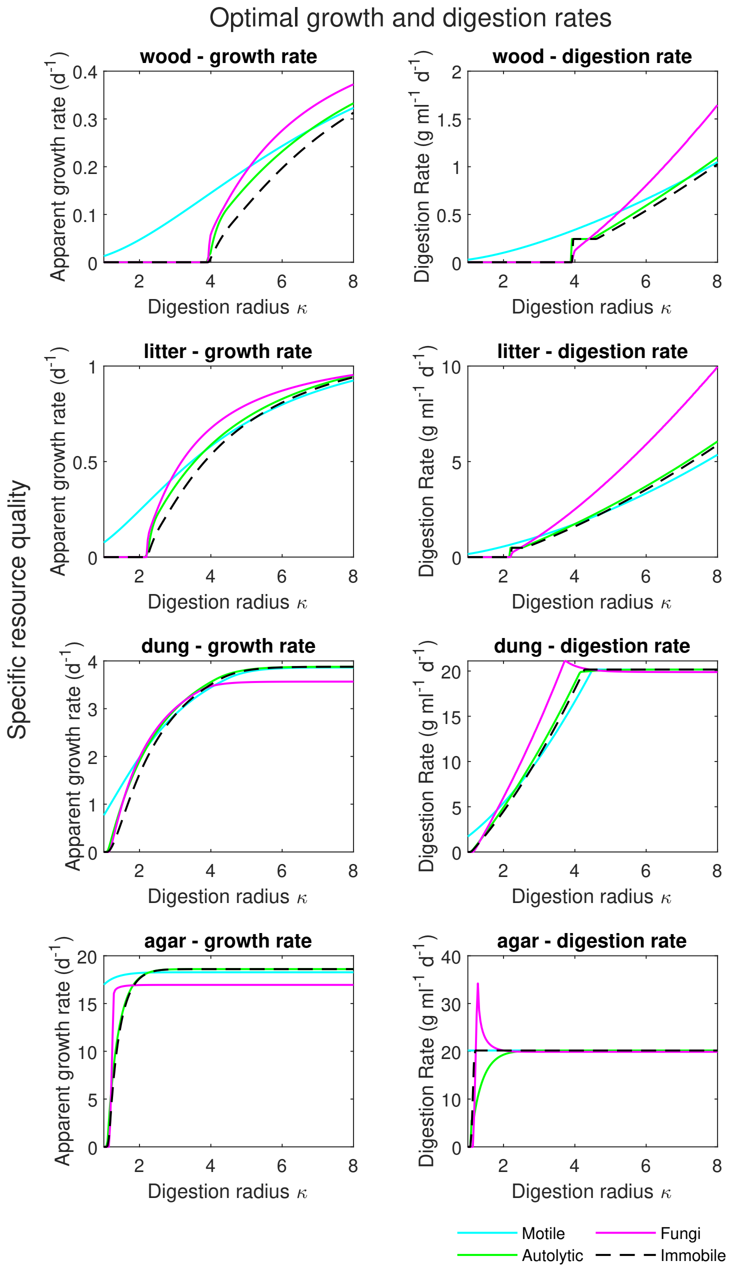


*Figure 3.2: Growth rate and digestion rate for a specific resource quality and quantity*
