# Supplementary material for: A mechanistic explanation of the transition to simple multicellularity in fungi
Source: Nat Commun. 2020 May 22;11:2594. doi: 10.1038/s41467-020-16072-4 (PMC7244713; doi:10.1038/s41467-020-16072-4)
Supplement: Supplementary file 3 — Peer Review [file 41467_2020_16072_MOESM3_ESM.pdf]

Reviewers' comments:

Reviewer #1 (Remarks to the Author):

This is a beautifully written manuscript with excellent diagrams that clarify the text. The hypothesis that fungal hyphae explore and exhaust resources while transporting newly acquired resources (e.g., C, N, and P) to older part of the cytoplasmic community is not new, but it is very nicely extended here to explain (or at least hypothesize) how multicellularity evolved. I would note that the same logic can be applied to coenocytic algae that employ a "search" strategy to "explore" substrate resources.

Reviewer #2 (Remarks to the Author):

My name is Laszlo G. Nagy, a proponent of open review. Using a computational analysis, Heaton et al explain why a hyphal thallus is a best-fit solution in habitats where nutrient sources are spatially heterogeneous. I think the ms provides a much needed explanation for why fungi live where they live and why they are dominant microbes in several key processes, such as wood-decay and mycorrhizal associations. I really enjoyed reading the ms and I only have some smaller, discretionary comments for the authors.

Main comments:
I feel that mixed concepts exist for exoenzyme synthesis and transport in the ms. At some places it is assumed that enzymes are secreted by the tip and remain in place and active (l146), whereas in other places long-range transport of the enzymes themselves are assumed (l250-252). This latter feels problematic to me (see below).

A cost for synthesising exoenzymes - I think this would be an important parameter for such a model, yet it seems like this wasn’t incorporated. See comment below and suggestion for additional analyses incorporating this parameter.

I feel a small disconnect between multicellularity and what the authors are testing. Strictly speaking, their model explains why tubular structures like hyphae thrive in habitats where nutrient sources are spatially heterogeneous. It is clear that a hyphal structure is adaptive in this case, but the model doesn’t explain why a multicellular hypha is so. I realize it is hard to separate the emergence of multicellularity from the emergence of hyphae in fungi, but I feel that the results pertain to the tubular nature of hyphae and not necessarily to multicellularity and so some effort is in order. Maybe this could be mentioned in the discussion (coenocytic vs septate hyphae) and the title/abstract slightly reconsidered. This does not reduce the coolness of the study in my eyes, however.

I feel the abstract is a bit technical and does not highlight the far-reaching implications of this work. I suggest mentioning how hyphae predisposed fungi for being mycorrhizal partner of plants too.

Specific comments:
l48 - might be worth noting that hyphae follow a fractal-like branching pattern, which confers additional advantages in exploring the substrate and avoiding conflict
l55 - or even earlier than hyphal MC, in unicellular chytrids? See Chang et al 2015 GBE on pectinolytic capabilities of early fungi.
l63 - “a carbon use efficiencies”
Fig1 legend: synthesis —> synthesise
l90 - remove ‘specific’
l121-134 - was a cost for synthesising exoenzymes built into the model? This sounds like an important parameter to me, with a potential to take a toll on how advantageous hyphal growth is. This might be worth adding to the model and running some additional analyses. Alternatively, the assumption of enzyme synthesis having a negligible cost should be mentioned here. Also, I suggest you mention the

parameter for the cost of transport here (its only in the supplement now).
l154-155 - I think you don't need to assume that enzymes are only synthesised in the hyphal tip.
l229-230 - note here that its because of the ability to transport assimilates.
l248 - is there evidence for the transport of hydrolases (better written as exoenzymes - btw, please standardise terminology in the ms)
l250 - delete 'eukaryotic'
l250-252 - isn't vesicle-enabled macromolecule transport restricted to cell wall components and the corresponding enzymes? Refs 30-32 point in this direction, rather than a useful long-term transport of assimilates or hydrolytic enzymes.
l255 - 'h yphal morphology inevitably results' this sounds like a case of evolutionary determinism, which is usually suggested for phenotypes that emerged repeatedly several times. Maybe just say 'advantageous/adaptive'

Fig4 suggests that there are specific substrates where fungi have an advantage irrespective of being terrestrial or aquatic. This has two implications:
- did this advantage drive the emergence of filamentous fungi in addition to the availability of terrestrial habitats?
- if this is indeed the case, why dont we see more aquatic fungi dominating submerged complex polymers (wood)?

I like that the presentation of the model precedes the results (even though, strictly speaking that would be the methods section), I hope the Editors will allow this sequence of chapters to be retained.

An interesting experiment with this model would be to assess the extent of conflict between individual hyphae when their assimilation zones overlap. We speculated that the fractal-like filling of the space by the mycelium minimizes this conflict, but we did not test this experimentally. I realize this is out of scope for this paper, just dropping the idea here.

Reviewer #3 (Remarks to the Author):

Heaton et al report on computational modelling of nutrient uptake and growth for different types of cellular organization. The authors compare fungal hyphae with various modes of unicellular organization. The computational modelling supports the conclusion that hyphal organization is advantageous when C/N ratio is high and the substrate is recalcitrant. They argue that the hyphal advantage is based on a continuous cytoplasm which enables transport and efficient mining of resources over time. The idea that hyphal organization represents an optimal solution to breaking down recalcitrant substrates is a long held view in mycology and is not in itself conceptually original. However, the extent to which the results of modelling can be considered definitive make this a potentially important study.

Major points.
1. The forms of cellular organization analysed by modelling correspond to existing organisms. However, the origin of "autolytic cell" is unclear. Do such cell types exist, or is this form of organization included as a theoretical alternative? If this type of organization is absent in nature, it surely suggests that it is a poor strategy. Thus, it is difficult to understand how motile and autolytic strategies appear to perform similarly well under many conditions (Figure 4). The authors should address this and provide more context on these issues.
2. The model of hyphae allows for vesicle transport throughout the entire mycelium. However, it is long known that multicellular fungal colonies possess a "peripheral growth zone" where hyphal compartments actively contribute to tip growth (Journal of General Microbiology (1971),67,325-344). Septa in the peripheral growth zone are unplugged, while older septa are plugged. Moreover, the proteins associated with the formation of these plugs have been identified

(www.pnas.org/cgi/doi/10.1073/pnas.1207467109). Plugs preclude vesicle trafficking, and this leads to the question of how the results from modelling would be affected if the authors imposed a peripheral growth zone. The authors may wish to argue that this consideration is excluded for simplicity. In this event, they should still make reference to the peripheral growth zone and this form of pore plugging since they are wel-established aspects of hyphal colony organization.
3. Page 10, lines 186-192: when "a unit volume captures less resource than is needed to grow a unit volume", it would be reasonable to imagine that the connectivity of a hyphal network would give fungi an advantage. However, the model predicts that motile cells will dominate. Is this because the cost of vesicle trafficking is too high? Along similar lines, different costs of vesicle trafficking (β) are examined in Fig. 4 and Supplementary Fig. 2. However, κ is also changed (in Fig. 4: κ = 2, 4, 6 cell radii, and in Supplementary Fig. 2, κ = 3, 5 cell radii). If there is no reason for using different sets of values, please standardize the κ values to make it easier to compare these figures.
4. Page 13, Fig. 5c: Why is digestion rate of motile cells > 0.3 g ml-1 hr-1 even when κ = 1? According to Fig. 3a, when κ = 1, the extracellular volume from where cell can obtain nutrients is 0. Given this, how is a digestion rate greater than 0 possible? Related to this point, it would be helpful if the authors explain why the digestion rate of motile cells is always greater than that of other cell types when κ=1.
5. The discussion lacks specific references to the author's figures, so it is difficult to follow and understand how the conclusions are drawn from their data.
6. In the discussion, the distinction between "metabolic theory" and the "information-centric perspective" theories of evolutionary transitions are not likely to be familiar to a broad audience, so their relevance to the present study should be explained in greater depth.

Minor points.
1. Supplementary information, page 1, lines 12 to 17: the authors conclude that the overall C:N ratio required for growth is 6:1. However, if half of C is allocated to biosynthesis and half is lost through respiration and the cells C:N ratio is 12:1, then should not the overall C:N requirement for growth be 24:1?

2. Page 6, lines 112-113: apparent growth rate is defined as "volume colonised per unit time per unit volume colonised". This is a bit confusing. By contrast the full definition from the supplements is better. I suggest using the definition from the supplement in the main text.

A mechanistic explanation for the transition to simple multicellularity

Heaton, L.L.M., Jones, N.S. and Fricker, M.D.

Response to reviewers

Reviewer #1 (Remarks to the Author):

This is a beautifully written manuscript with excellent diagrams that clarify the text. The hypothesis that fungal hyphae explore and exhaust resources while transporting newly acquired resources (e.g., C, N, and P) to older part of the cytoplasmic community is not new, but it is very nicely extended here to explain (or at least hypothesize) how multicellularity evolved. I would note that the same logic can be applied to coenocytic algae that employ a "search" strategy to "explore" substrate resources.

> We thank the reviewer, and note that we have added the following comment to the second paragraph of the discussion (line 292-295), with a reference to Niklas et al, 2013, Evolution and Development:
>
> "Our argument may also explain why algae that need to search for substrate resources have evolved a siphonous body plan, with multiple nuclei in a common cytoplasmic pool, and a capacity for indeterminate growth in size."

Reviewer #2 (Remarks to the Author):

My name is Laszlo G. Nagy, a proponent of open review. Using a computational analysis, Heaton et al explain why a hyphal thallus is a best-fit solution in habitats where nutrient sources are spatially heterogeneous. I think the ms provides a much needed explanation for why fungi live where they live and why they are dominant microbes in several key processes, such as wood-decay and mycorrhizal associations. I really enjoyed reading the ms and I only have some smaller, discretionary comments for the authors.

We thank the reviewer for the detailed and helpful comments on the manuscript. We have responded to each comment below, and made alterations to the manuscript and SI as recommended.

Main comments:

I feel that mixed concepts exist for exoenzyme synthesis and transport in the ms. At some places it is assumed that enzymes are secreted by the tip and remain in place and active (l146), whereas in other places long-range transport of the enzymes themselves are assumed (l250-252). This latter feels problematic to me (see below).

> We assume that nutrients are pooled across the fungal colony, and assume that fungi face additional costs due to the transport of exoenzymes and other materials. However, strictly speaking, we do not need to assume long-range transport of exoenzymes. To clarify this point, we have changed the description of the cost of transport, so it now reads as follows (lines 173-176):
>
> "The additional costs associated with internal transport are unknown, but are represented by a tunable parameter $\beta = 0.1$ i.e. 10% of the total C and N budget of the cell (see Fig. 3c). The fraction β can be visualised as the fraction of C and N that is contained in exoenzymes or cell wall material moving within transport vesicles, although in modelling terms, the

transported material could equally well be precursors moving cytoplasmically. The critical point is that they are transported over distance."

A cost for synthesising exoenzymes - I think this would be an important parameter for such a model, yet it seems like this wasn't incorporated. See comment below and suggestion for additional analyses incorporating this parameter.

The cost for synthesising the exoenzymes is included in the model, but we have also extensively clarified the text (and the programs) to make this point far more explicit. We give a full explanation in response to the more detailed comment below.

I feel a small disconnect between multicellularity and what the authors are testing. Strictly speaking, their model explains why tubular structures like hyphae thrive in habitats where nutrient sources are spatially heterogeneous. It is clear that a hyphal structure is adaptive in this case, but the model doesn't explain why a multicellular hypha is so. I realize it is hard to separate the emergence of multicellularity from the emergence of hyphae in fungi, but I feel that the results pertain to the tubular nature of hyphae and not necessarily to multicellularity and so some effort is in order. Maybe this could be mentioned in the discussion (coenocytic vs septate hyphae) and the title/abstract slightly reconsidered. This does not reduce the coolness of the study in my eyes, however.

We agree that strictly speaking our model is concerned with why hyphal structures are adaptive, not multicellularity per se. Hence we have changed the discussion so that the first lines (l263-268) read as follows:

"Our model suggests that when there is a small, exhaustible local supply of some essential nutrient, and a larger supply of other nutrients, it is evolutionarily adaptive for cells to grow as an interconnected network with a common cytoplasmic pool. As growing hyphal organisms contain an expanding volume of cytoplasm, they can only maintain a constant nuclear density by containing multiple nuclei. Hence a transition to hyphal morphologies also entails a transition to either coenocytic, or fully multicellular, modes of life."

We have also clarified that we are only considering *simple* multicellularity in the title and text, rather than complex multicellularity involved in fruiting body formation.

I feel the abstract is a bit technical and does not highlight the far-reaching implications of this work. I suggest mentioning how hyphae predisposed fungi for being mycorrhizal partner of plants too.

We would like to include a reference to mycorrhizae in the abstract, but were not able to due to the word limit of 150 words. Indeed, we have had to delete a sentence to keep within the 150 word limit.

Specific comments:

l48 - might be worth noting that hyphae follow a fractal-like branching pattern, which confers additional advantages in exploring the substrate and avoiding conflict

We have added a reference to the space-searching efficiency of fungi (Hanson et al., 2006 [35]), and added the following mention of fractal like branching:

(L52-54) "The polarised growth and fractal like branching of mycelial fungi constitute an efficient space-searching strategy[35], and large interconnected mycelial networks can span soil gaps and patches of nutrients."

l55 - or even earlier than hyphal MC, in unicellular chytrids? See Chang et al 2015 GBE on pectinolytic capabilities of early fungi.

We thank the referee for pointing out this very interesting reference. We have added references to Chang et al 2015 ([28]), and changed the text to read as follows:

(L38-39) " … with subsequent loss of flagella and the development of multicellular hyphal growth linked to adaptation to land environments[8,20-22,28]."

L46-49 " …recalcitrant polymers such as pectins, chitin and lignin[20,24,26]. Indeed, genetic evidence suggests that the production of exoenzymes to digest plant or algal cell walls is an ancestral trait, found in unicellular chytrids[28] that predate the earliest forms of hyphal multicellularity, or adaptation to terrestrial environments[20,21].

l63 - "a carbon use efficiencies"
Fig1 legend: synthesis —> synthesise
l90 - remove 'specific'

The text has been changed to correct these typos

l121-134 - was a cost for synthesising exoenzymes built into the model? This sounds like an important parameter to me, with a potential to take a toll on how advantageous hyphal growth is. This might be worth adding to the model and running some additional analyses. Alternatively, the assumption of enzyme synthesis having a negligible cost should be mentioned here. Also, I suggest you mention the parameter for the cost of transport here (its only in the supplement now).

Following this comment and the comments from the other reviewers, we have clarified the significance of carbon use efficiency by using C_I to refer to the mass of carbon contained in a unit volume of organism, while C_T refers to the total mass of C required per unit volume of growth, including any C lost by respiration, defined by the Carbon Use Efficiency, CUE. CUE was previously included implicitly in the model, but has now been made explicit throughout the main text, equations, and Matlab programs.

(L164-167) "Since organisms lose C through respiration, growing a unit volume of immobile cell requires a total mass of carbon $C_T > C_I$, and this total carbon cost of growing a unit volume is specified by including the CUE as a model parameter."

As should now be clear from the text and figure captions, we assume that for unicellular organisms, the cost of synthesising exoenzymes is x $(C_T + N_I)$, where x is the mass of exoenzymes relative to the mass of the cell. For fungi, the corresponding cost is $x(1+ \beta)(C_T +$ x $N_I)$, to account for the fact that a certain amount of costly transport is required when fungi release exoenzymes. Thus, in all cases the cost of synthesizing the exoenzymes is included. For any given value of x, the rate of digestion is proportional to x, and inversely proportional to the recalcitrance parameter τ. The value of x varies from case to case, but it is always chosen to maximise the growth rate of the organism.

l154-155 - I think you don't need to assume that enzymes are only synthesised in the hyphal tip.

We have included an additional paragraph and references in the SI that refers to our current understanding of where exoenzymes are secreted (SI L232-240).

"We assume that exoenzymes are predominantly secreted at the hyphal tips (Archer and Peberdy, 1997), which has been demonstrated experimentally for a number of exoenzymes (Wosten et al., 1991, Cai et al., 1999). Furthermore, spatially explicit gene expression studies support this view, with genes involved in protein synthesis and exoenzyme production

expressed at the colony periphery, whilst genes coding for transporters and permeases are located at the colony centre (Masai et al. 2006). Nevertheless, if exoenzymes were secreted all over the mycelium, the amount in transit would be lower and the cost associated with hyphal transport (β) would be reduced. However, each enzyme would have access to less total resource over its lifespan, as colonised resource would already be partially digested, so secreting exoenzymes at locations distal to the tip would be less efficient."

Strictly speaking our model does not assume anything about where enzymes are synthesised, we simply assume that all materials obtained by the fungus are pooled, and there is a cost associated with internal transport determined by parameter β.

l229-230 - note here that its because of the ability to transport assimilates.

We have altered the text to read as follows (L270-274):

"In general, the exoenzymes of unicellular organisms can contribute to growth for at most time T (that is, the time to exhaust the most growth-limiting resource), but by forming an interconnected network that enables the transport of assimilated materials, multicellular fungi effectively extend the useful lifespan of any exoenzyme that digests a non-growth-limiting resource.

l248 - is there evidence for the transport of hydrolases (better written as exoenzymes - btw, please standardise terminology in the ms)

We have replaced all occurrences of 'hydrolases' with 'exoenzymes'. As for the transport of hydrolases, see previous comments about vesicles representing the cost of transport of exoenzymes or their precursors, not being a literal claim that this is the only mode of transport.

l250 - delete 'eukaryotic'

We have replaced the sentence in question with the following:

(L303-305) "Both fungi and actinomycetes can transport materials by diffusion and growth-induced mass-flow, but as fungi are eukaryotic, they can also use internal vesicles, the cytoskeleton and motor proteins, which enables greater control over the internal distribution of macromolecules."

l250-252 - isn't vesicle-enabled macromolecule transport restricted to cell wall components and the corresponding enzymes? Refs 30-32 point in this direction, rather than a useful long-term transport of assimilates or hydrolytic enzymes.

There is evidence for long-distance transport of solutes in fungi, but the precise transport pathway is not clear. We discuss this in more depth in response to Referee 3 below. Nevertheless, as we comment above, the precise mode of transport is not critical, simply that there is a cost to moving precursors or exoenzymes themselves from the site of nutrient uptake to the site of secretion.

l255 - 'hyphal morphology inevitably results' this sounds like a case of evolutionary determinism, which is usually suggested for phenotypes that emerged repeatedly several times. Maybe just say 'advantageous/adaptive'

We have replaced the sentence in question with the following:

(L308-310) "Our analysis strongly suggests that whenever hyphal colonies emerge, the mature parts will be a source of non-rate limiting resource (C), while the growing margin obtains growth limiting resource (N or P)."

Fig4 suggests that there are specific substrates where fungi have an advantage irrespective of being terrestrial or aquatic. This has two implications:
- did this advantage drive the emergence of filamentous fungi in addition to the availability of terrestrial habitats?
- if this is indeed the case, why dont we see more aquatic fungi dominating submerged complex polymers (wood)?

This is an interesting point. However, we have not considered the aquatic environment in the model further as we would need to explicitly model diffusion. Nevertheless, we infer that the relative ease of diffusion of nutrients when in aqueous media compared to an immobile substrate would decrease the hyphal advantage. Furthermore, extracellular digestion in an aquatic environment with increased diffusion would potentially lead to complex social interactions for accessible public goods, which is beyond the scope of this paper. To clarify the connection between specifically terrestrial habitats and our model assumptions, we have added the following sentences to the introduction:

(L 64-69) "It follows that nutrient supply has a critical impact on growth rates. Colonisation of land presents additional challenges as net movement of soluble nutrients is reduced in terrestrial compared to aquatic environments, as the soil pore tortuosity increases the path length for diffusion. Furthermore, the requirement to breakdown C-rich polymers produces even greater spatial inhomogeneities in nutrient concentration as these do not diffuse at all."

I like that the presentation of the model precedes the results (even though, strictly speaking that would be the methods section), I hope the Editors will allow this sequence of chapters to be retained.

An interesting experiment with this model would be to assess the extent of conflict between individual hyphae when their assimilation zones overlap. We speculated that the fractal-like filling of the space by the mycelium minimizes this conflict, but we did not test this experimentally. I realize this is out of scope for this paper, just dropping the idea here.

We agree with the referee that this is an interesting and biologically relevant point. We did originally present this in part the SI (Section 8), but did not explicitly refer to the result in the main text. We have now included the following paragraph in the results:

(L 247 onwards) "In the early stages of colonisation none of the cells will have exhausted the local supply of resources. However, we can also consider the case where the resources available to daughter cells are diminished due to overlap with parental cells. In this case, there is more complex time-dependent decline in the apparent growth rate for each class of organisms, depending on the total supply of resource available (SI Fig. S5). However, as the fraction of the colony that has exhausted some, but not all, essential nutrients is the part that hyphal architecture is able to exploit more effectively, the apparent growth rate of fungi declines to a lesser extent and the hyphal advantage is increased (SI Section 8)."

Reviewer #3 (Remarks to the Author):

Heaton et al report on computational modelling of nutrient uptake and growth for different types of cellular organization. The authors compare fungal hyphae with various modes of unicellular organization. The computational modelling supports the conclusion that hyphal organization is advantageous when C/N ratio is high and the substrate is recalcitrant. They argue that the hyphal advantage is based on a continuous cytoplasm which enables transport and efficient mining of resources over time. The idea that hyphal organization represents an optimal solution to breaking down recalcitrant substrates is a long held view in mycology and is not in itself conceptually original. However, the extent to which the results of modelling can be considered definitive make this a potentially important study.

We thank the reviewer for their constructive comments and we have provided additional text and references to clarify each of the points raised. We also thank them for pointing out the error in the introduction to the SI, which has been corrected.

Major points.

1. The forms of cellular organization analysed by modelling correspond to existing organisms. However, the origin of "autolytic cell" is unclear. Do such cell types exist, or is this form of organization included as a theoretical alternative? If this type of organization is absent in nature, it surely suggests that it is a poor strategy. Thus, it is difficult to understand how motile and autolytic strategies appear to perform similarly well under many conditions (Figure 4). The authors should address this and provide more context on these issues.

> In the model description in the main text we have added the following sentence, and included a reference (Benomar et al, 2015, Nat. Comms.)[48].
>
> (L147-149) "Data suggests that when nutritionally stressed cells recycle their nutrient contents through autolysis, the growth rate of neighbouring cells is increased[48]."
>
> We have included a more discussion in the SI when first introducing autolytic cells:
>
> (SI L136 onwards) "We assume that once an autolytic cell exhausts the local resource a fraction ε of the resource used to grow the cell becomes instantly and freely available to the remaining cells, though any resource used to synthesise exoenzymes cannot be recouped. This represents a simple model for cells undergoing autolysis or more controlled programmed cell death (PCD). Whilst these destructive processes lower the fitness of individual cells, there is increasing evidence that it provides an advantage to neighbouring cells via recycling resources, reducing stress responses or enhancing biofilm formation (Benomar et al., 2015, Durand et al, 2016; Allocati et al., 2015)[14-16]. Furthermore, computer simulations show that recycling alone can confer a benefit for PCD (Vostinar et al., 2019)[17]. It is noteworthy that in the context of degradation of immobile resources, there is probably a higher probability that neighbours are related kin, which might support adaptation at a group level (Nedelcu et al., 2011)[18]."

2. The model of hyphae allows for vesicle transport throughout the entire mycelium. However, it is long known that multicellular fungal colonies possess a "peripheral growth zone" where hyphal compartments actively contribute to tip growth (Journal of General Microbiology (1971),67,325-344). Septa in the peripheral growth zone are unplugged, while older septa are plugged. Moreover, the proteins associated with the formation of these plugs have been identified (www.pnas.org/cgi/doi/10.1073/pnas.1207467109). Plugs preclude vesicle trafficking, and this leads

to the question of how the results from modelling would be affected if the authors imposed a peripheral growth zone. The authors may wish to argue that this consideration is excluded for simplicity. In this event, they should still make reference to the peripheral growth zone and this form of pore plugging since they are well-established aspects of hyphal colony organization.

This is a very interesting point raised by the referee and warrants more discussion. We have therefore included an additional paragraph and references, including the original Trinci 1971 paper, in the SI:

(SI L248-260) "One interesting aspect of transport that we have not yet modelled is the transport pathway itself. The ancestral state still present in lower fungi like the Zygomycota is thought to comprise a septate coenocytic hyphae, that would conform well to the model simulation. However, cell compartments in ascomycetes and basidiomycetes are separated by septal pores, which provide cytoplasmic continuity when open, but can be rapidly blocked to prevent leakage if hyphae are damaged (Jedd 2011). Numerous studies have also shown that there is an increasing probability of septal pore closure during normal development moving distal from the hyphal tips through the peripheral growth zone (Trinci 1971, Markham 1994, Jedd 2011, Tegelaar 2017, Bleichrodt et al., 2015, vanPeer 2009}, which would impact on the long-distance transport modelled here. Equally, there is some evidence that pore closure is dynamic and dependent on the resource environment (vanPeeret al., 2009), and that small radiolabelled solute molecules may still move selectively through hyphae even with closed septa (Bleichrodt et al. 2015). There is also strong evidence from radiotracer studies for extensive, rapid long-distance transport through mycelial networks over centimetres (Tlalka et al., 2002, Tlalka et al., 2008, Olsson 1998, Lindahl et al., 2001, Jennings 1987, Cairney 1992}, although the precise pathway is unknown (Fricker et al., 2017)."

We have also clarified the manuscript by making clear that the vesicles in our model are a way of including a cost of transport, but do not preclude other methods of transport, as found by Bleichrodt et al., 2015. Specifically, in the main text and the supplementary information, we have replaced the phrase "vesicle transport cost" with "internal transport cost", and have added the following sentences to our description of the model:

(L171-176) "The additional costs associated with internal transport are unknown, but are represented by a tunable parameter $\beta = 0.1$ i.e. 10% of the total C and N budget of the cell (see Fig. 3c). The fraction β can be visualised as the fraction of C and N that is contained in exoenzymes or cell wall material moving within transport vesicles, although in modelling terms, the transported material could equally well be precursors moving cytoplasmically. The critical point is that they are transported over distance."

3. Page 10, lines 186-192: when "a unit volume captures less resource than is needed to grow a unit volume", it would be reasonable to imagine that the connectivity of a hyphal network would give fungi an advantage. However, the model predicts that motile cells will dominate. Is this because the cost of vesicle trafficking is too high?

If the cost of growth is greater than the benefit, organisms cannot survive if they only obtain new resource through growth. To clarify this point, we have altered the relevant sentence. It now reads:

(L211-216) "However, when a unit volume captures less resource than is needed to grow a unit volume (low values of κ, Fig. 5), capturing resource by growth alone is a losing proposition. Under those conditions motile cells are predicted to dominate (cyan regions in Fig. 4), as they are able to move to multiple sites to access the nutrients needed for duplication, provided that the resource is sufficiently water saturated to enable cell migration (Dechesne et al., 2010)."

Along similar lines, different costs of vesicle trafficking (β) are examined in Fig. 4 and Supplementary Fig. 2. However, κ is also changed (in Fig. 4: κ = 2, 4, 6 cell radii, and in Supplementary Fig. 2, κ = 3, 5 cell radii). If there is no reason for using different sets of values, please standardize the κ values to make it easier to compare these figures.

We have re-done all the relevant figures in the SI to span the same N:P ratios and κ as the main text.

4. Page 13, Fig. 5c: Why is digestion rate of motile cells > 0.3 g ml-1 hr-1 even when κ = 1? According to Fig. 3a, when κ = 1, the extracellular volume from where cell can obtain nutrients is 0. Given this, how is a digestion rate greater than 0 possible? Related to this point, it would be helpful if the authors explain why the digestion rate of motile cells is always greater than that of other cell types when κ=1.

When κ = 1, the volume of the source of nutrients is equal to the volume of the organism. Under those circumstances, obtaining nutrients through growth alone is a losing proposition (unless the substrate is exceptionally nutrient rich). Consequently, only motile organisms that capture additional nutrients through movement as well as growth are able to survive. To avoid confusion over the meaning of κ = 1, we have slightly altered Fig. 3.

5. The discussion lacks specific references to the author's figures, so it is difficult to follow and understand how the conclusions are drawn from their data.

More references to figures in the results and discussion sections of the main text, and also the associated figures in SI have been added to the results and discussion to aid cross-referencing. For example, the discussion now includes explicit reference to the SI figures:

(L297-301) "The advantage of a hyphal morphology is remarkably robust to changes in model parameters, most notably reducing the costs associated with motility (α, SI Fig. S2), increasing the benefit for autolytic cells (ε, SI Fig. S2), increasing the costs of transport (β, SI Fig. S3 & S4), imposing time-varying restrictions on resource availability due to crowding (SI Fig. 5 & 6), or increasing the requirement to digest C to render N accessible (δ, SI Fig. 7 & 8)."

6. In the discussion, the distinction between "metabolic theory" and the "information-centric perspective" theories of evolutionary transitions are not likely to be familiar to a broad audience, so their relevance to the present study should be explained in greater depth.

We have provided more explanation in the penultimate paragraph of the discussion as follows:

(L332-339) "In this framework the expansive energy available for growth and reproduction is a good approximation for fitness (van Valen, 1980, van Valen, 1976, Simpson 2011), and circumvents the problem of needing to define and count individuals and their reproductive

output central to other fitness measures, which is both conceptually and practically challenging for indeterminate or colonial organisms (Simpson, 2011, Pringle, 2002). Furthermore, the impact of competition for other resources can also be accommodated in the extent that they constrain control of trophic energy (van Valen, 1980, van Valen, 1976}. It is argued that natural selection locally maximises the amount of expansive energy for the unit under consideration at a given time scale (van Valen, 1980, van Valen, 1976)."

The final paragraph of the discussion now reads as follows:

(L 344-350) "Overall, we suggest that the emergence of hyphal organisms is best explained by changes in the available opportunities for metabolic activity (O'Malley, 2016), instead of viewing hyphal morphology as an evolutionary transition in individuality (Szathmary, 1995, Szathmary, 2015). The absence of a major transition in individuality is not surprising, as clonal hyphal organisms, in a similar manner to siphonous coenocytic organisms (Niklas, 2013b), have minimal cell-cell conflict and essentially bypass the alignment of fitness stage (Nagy, 2018), whilst the benefits of long-distance transport of nutrients through the connected cytoplasm directly leads to export-of-fitness at the level of the mycelial network (Knoll, 2016, Niklas, 2013b)."

Minor points.

1. Supplementary information, page 1, lines 12 to 17: the authors conclude that the overall C:N ratio required for growth is 6:1. However, if half of C is allocated to biosynthesis and half is lost through respiration and the cells C:N ratio is 12:1, then should not the overall C:N requirement for growth be 24:1?

We thank the reviewer for noticing that these two ratios were accidentally switched. The text now reads (SI L22) "... an internal molar C:N ratio of 6:1 and an N:P ratio of 14:1 is fairly typical. Thus, including the respiratory C requirement increases the overall C:N ratio that must be acquired for growth to 12:1."

2. Page 6, lines 112-113: apparent growth rate is defined as "volume colonised per unit time per unit volume colonised". This is a bit confusing. By contrast the full definition from the supplements is better. I suggest using the definition from the supplement in the main text.

We have followed this suggestion, and the first paragraph of 'Model Overview and Assumptions' now includes the following sentences:

(L118 onwards) "The volume colonised per unit time will increase in proportion to the size of the colony, and we define the apparent growth rate of a colony as the volume of resource that is colonised per unit time, per unit volume that has already been colonised. As our model specifies all the relevant quantities, for any given density of exoenzymes, we can calculate the apparent growth rate of the colony, and thereby find the optimal density of exoenzymes and the maximal rate of colonisation for any given category of organism"

REVIEWERS' COMMENTS:

Reviewer #1 (Remarks to the Author):

The authors have done a thorough job in addressing my concerns. In my opinion the manuscript is ready for publication.

Reviewer #2 (Remarks to the Author):

The authors have satisfactorily addressed all of my comments.
